# Supplementary material for: Human gene expression variability and its dependence on methylation and aging
Source: BMC Genomics. 2019 Dec 7;20:941. doi: 10.1186/s12864-019-6308-7 (PMC6898959; doi:10.1186/s12864-019-6308-7)
Supplement: Supplementary file 1 — Additional file 1. Structural analysis of genes as a function of EV [file 12864_2019_6308_MOESM1_ESM.pdf]

Additional File 1. Structural analysis of genes as a function of EV

# Largest Transcript Size Linear Regression Analysis

| | Tissue | Class | Intercept | Slope | P.value |
|---|---|---|---|---|---|
| [1,] | Breast | Hypervariable | 0.323368818 | −1.203087e−04 | 0.39126991 |
| [2,] | Breast | Hypovariable | −0.181547537 | −1.451642e−05 | 0.52287381 |
| [3,] | Breast | Non−Variable | −0.010076728 | 1.895844e−05 | 0.03180602 |
| [4,] | Cerebellum | Hypervariable | 0.150103540 | −3.367391e−05 | 0.23811395 |
| [5,] | Cerebellum | Hypovariable | −0.103916737 | −8.663306e−07 | 0.93910848 |
| [6,] | Cerebellum | Non−Variable | −0.006031851 | 1.015187e−05 | 0.12098736 |
| [7,] | Frontal Cortex | Hypervariable | 0.169934981 | 1.765995e−05 | 0.76494860 |
| [8,] | Frontal Cortex | Hypovariable | −0.114546512 | 1.493340e−05 | 0.39794470 |
| [9,] | Frontal Cortex | Non−Variable | −0.007274652 | 5.529679e−06 | 0.37786772 |

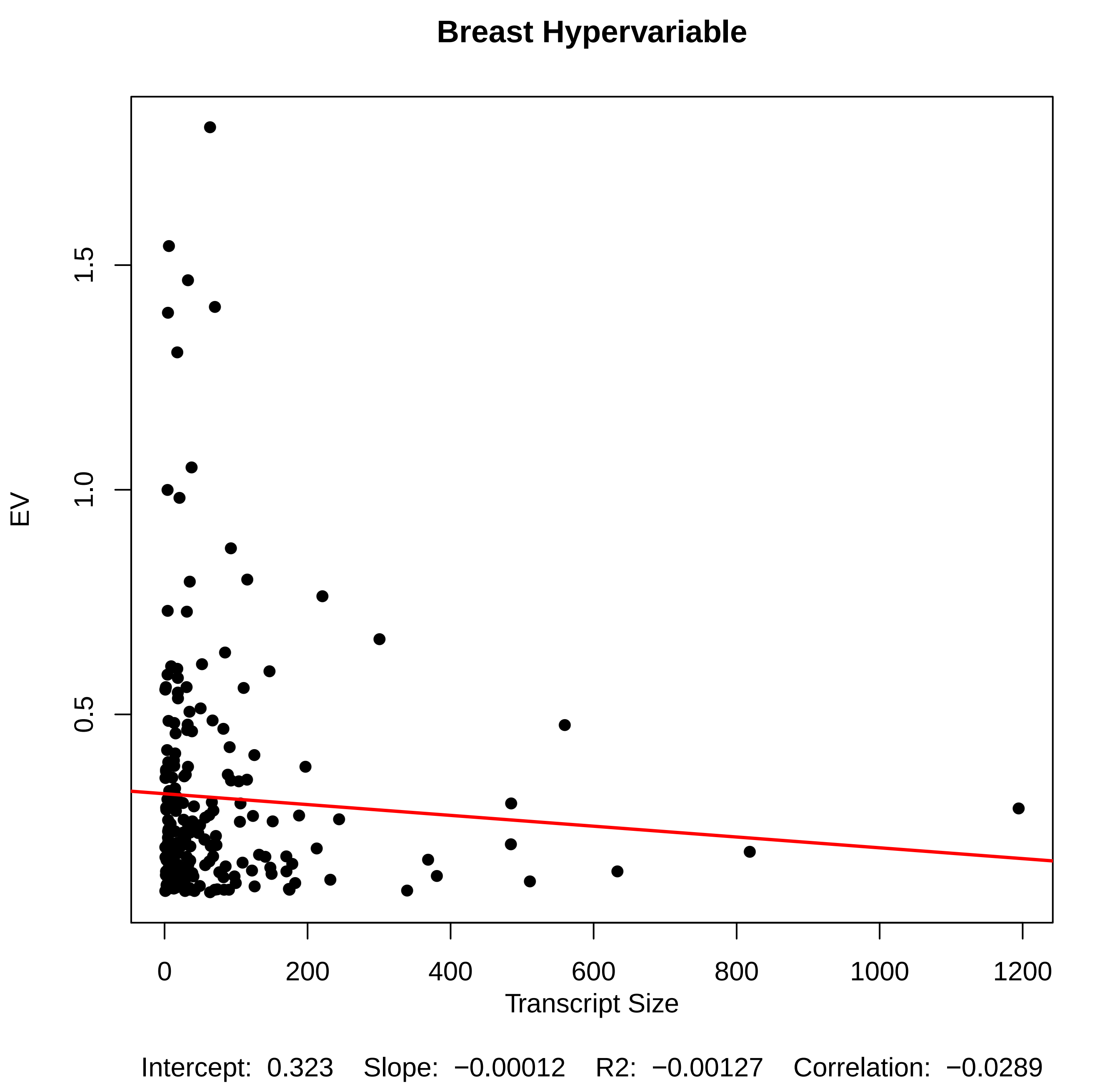

Breast Hypervariable
EV
0.5
1.0
1.5
0
200
400
600
800
1000
1200
Transcript Size
Intercept: 0.323 Slope: −0.00012 R2: −0.00127 Correlation: −0.0289

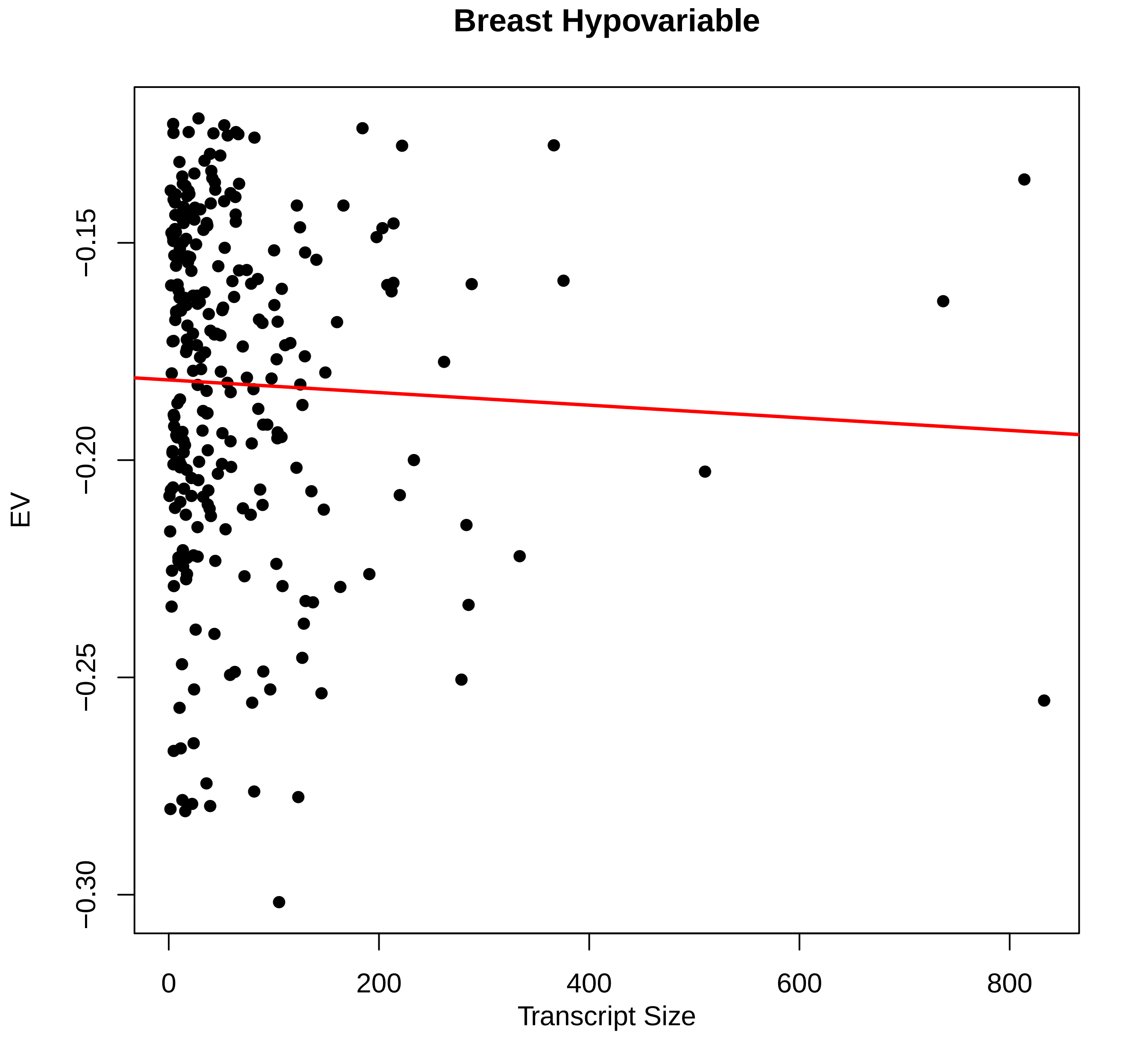


Intercept: −0.182   Slope: −1.45e−05   R2: −0.00223   Correlation: −0.0284

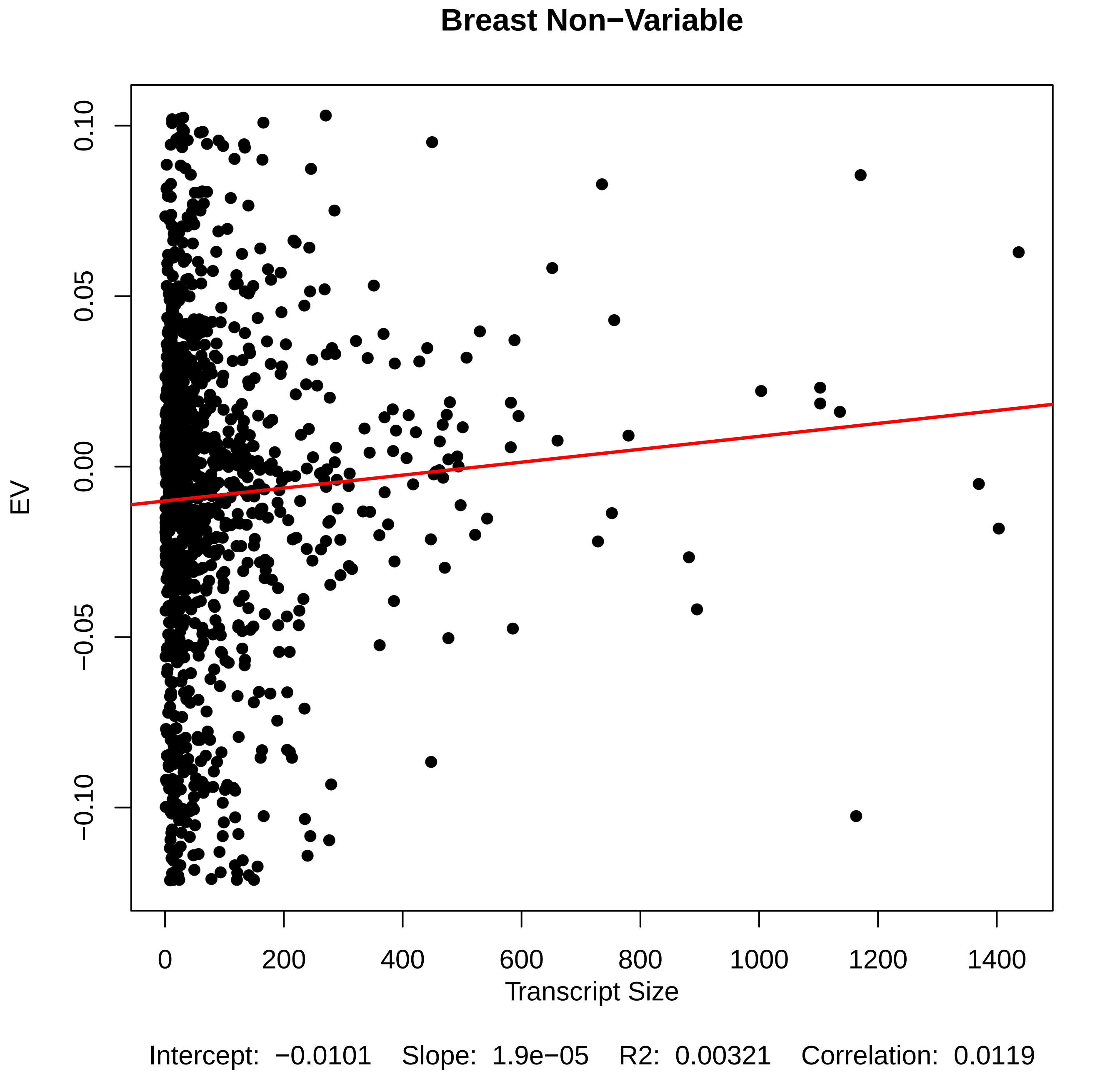
Breast Non−Variable
EV
0.10
0.05
0.00
−0.05
−0.10
0
200
400
600
800
1000
1200
1400
Transcript Size
Intercept: −0.0101 Slope: 1.9e−05 R2: 0.00321 Correlation: 0.0119

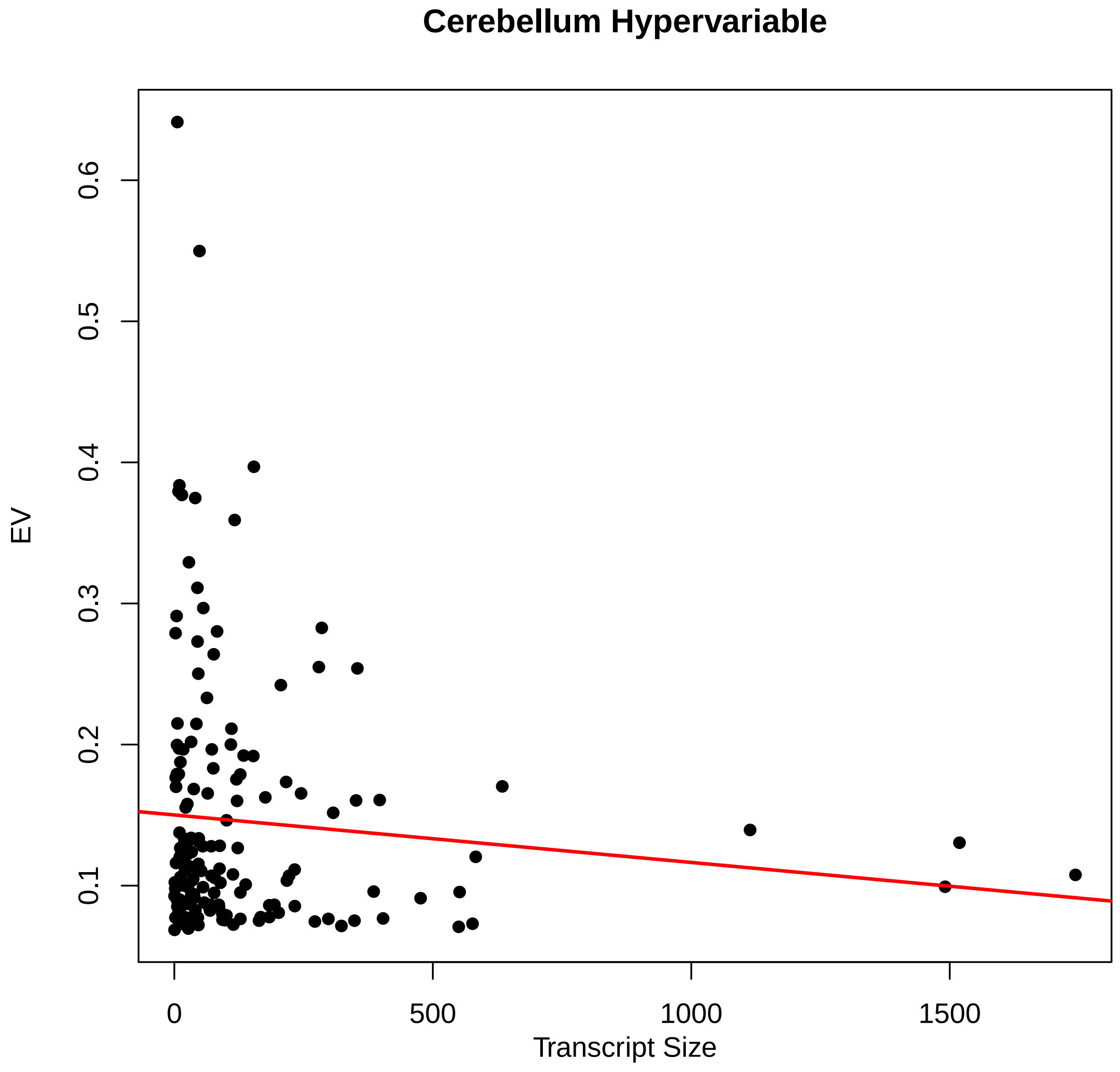


Intercept: 0.15 Slope: −3.37e−05 R2: 0.00251 Correlation: −0.047

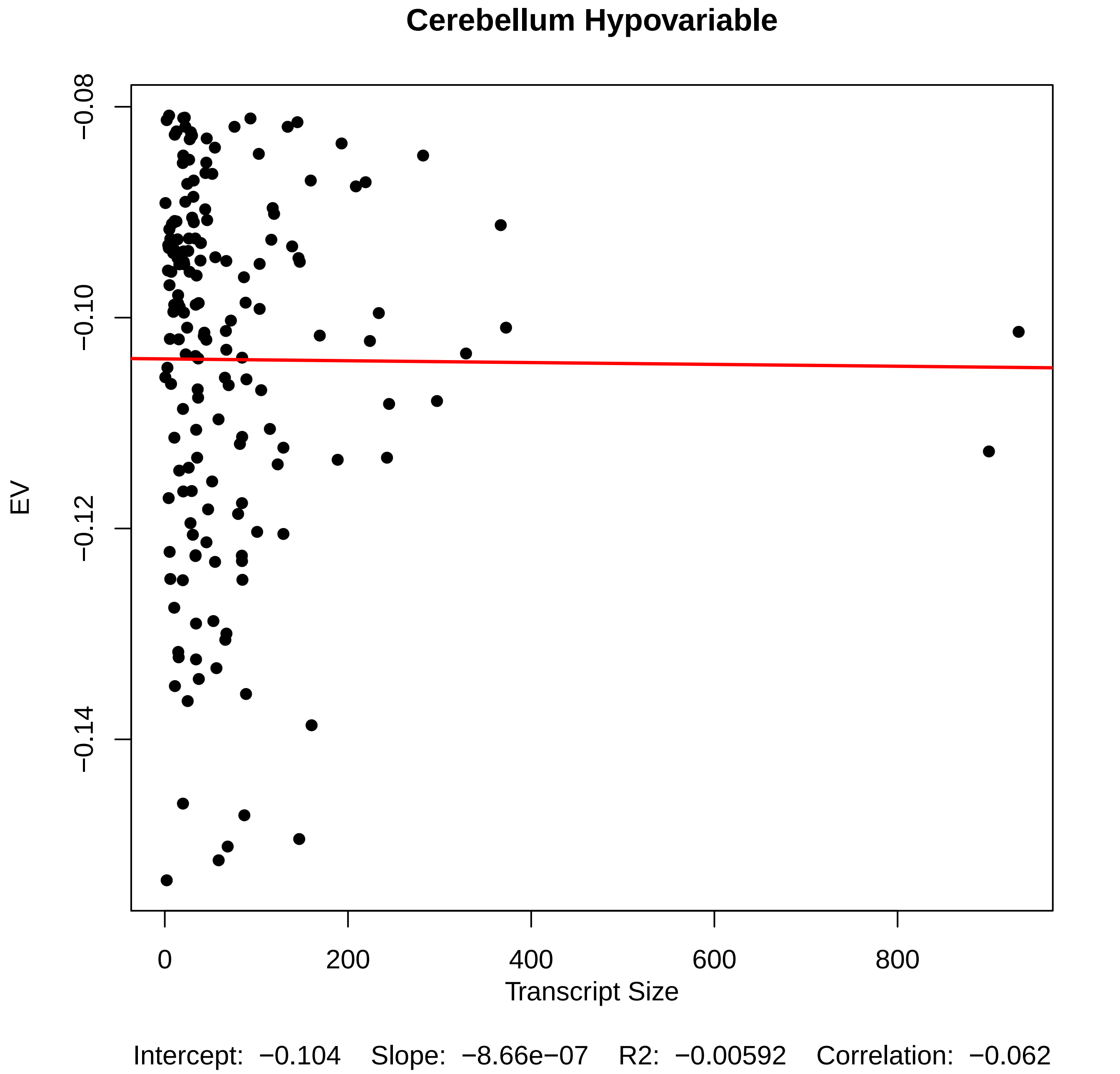
Cerebellum Hypovariable
−0.08
−0.10
−0.12
−0.14
EV
0
200
400
600
800
Transcript Size
Intercept: −0.104 Slope: −8.66e−07 R2: −0.00592 Correlation: −0.062

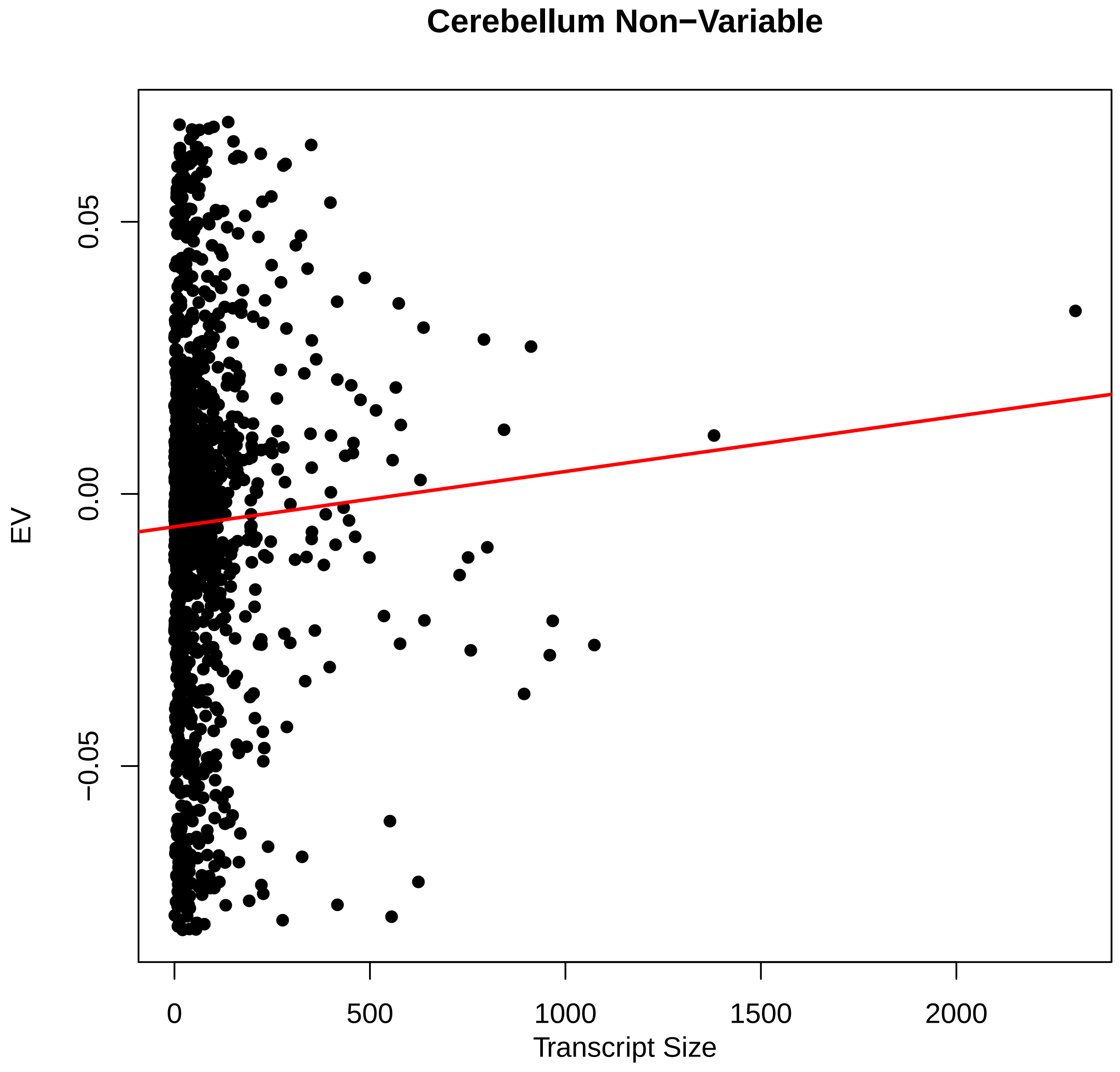


Intercept: −0.00603 Slope: 1.02e−05 R2: 0.00117 Correlation: 0.0309

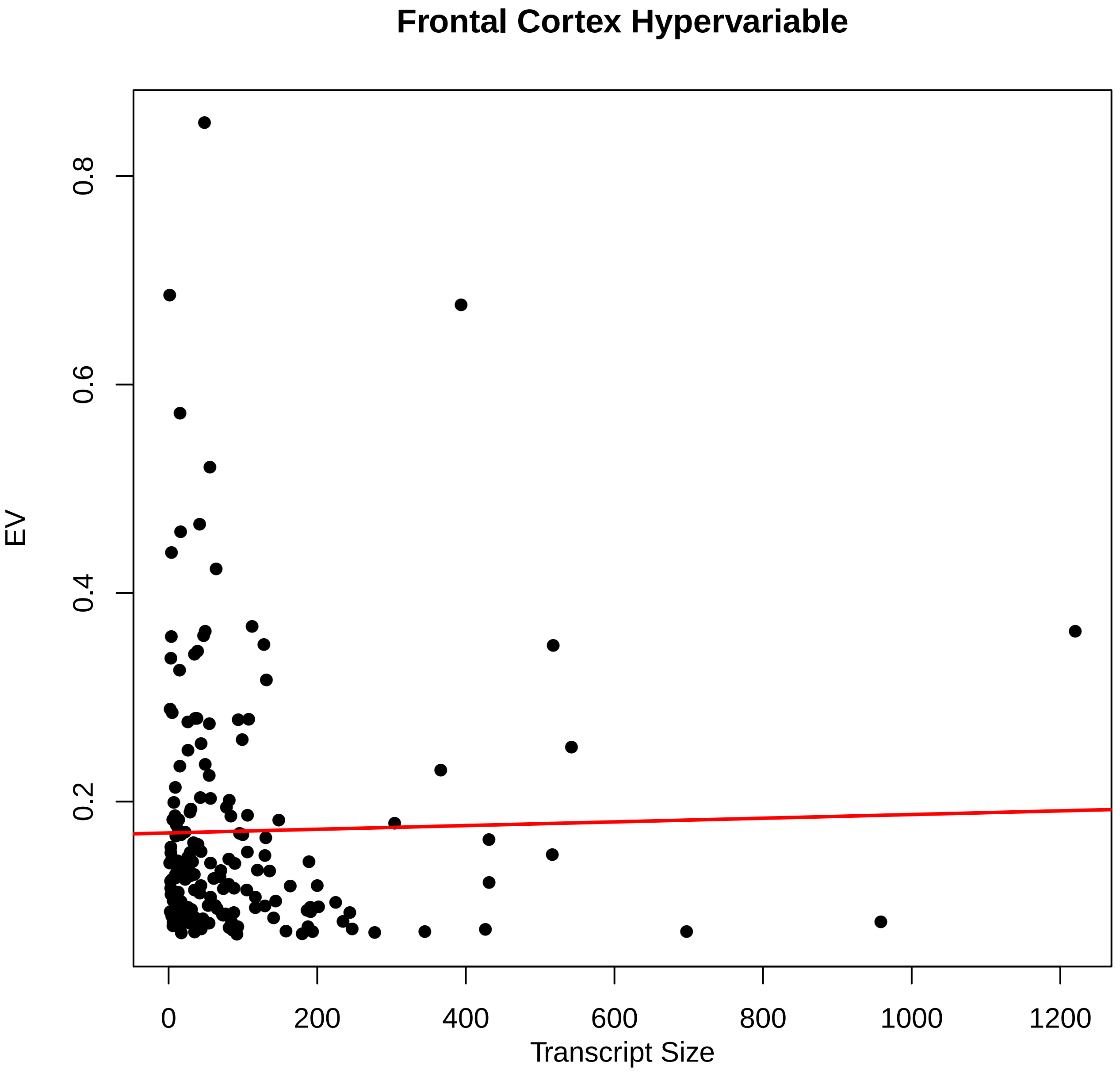


Intercept: 0.17 Slope: 1.77e−05 R2: −0.0052 Correlation: −0.094

## Frontal Cortex Hypovariable

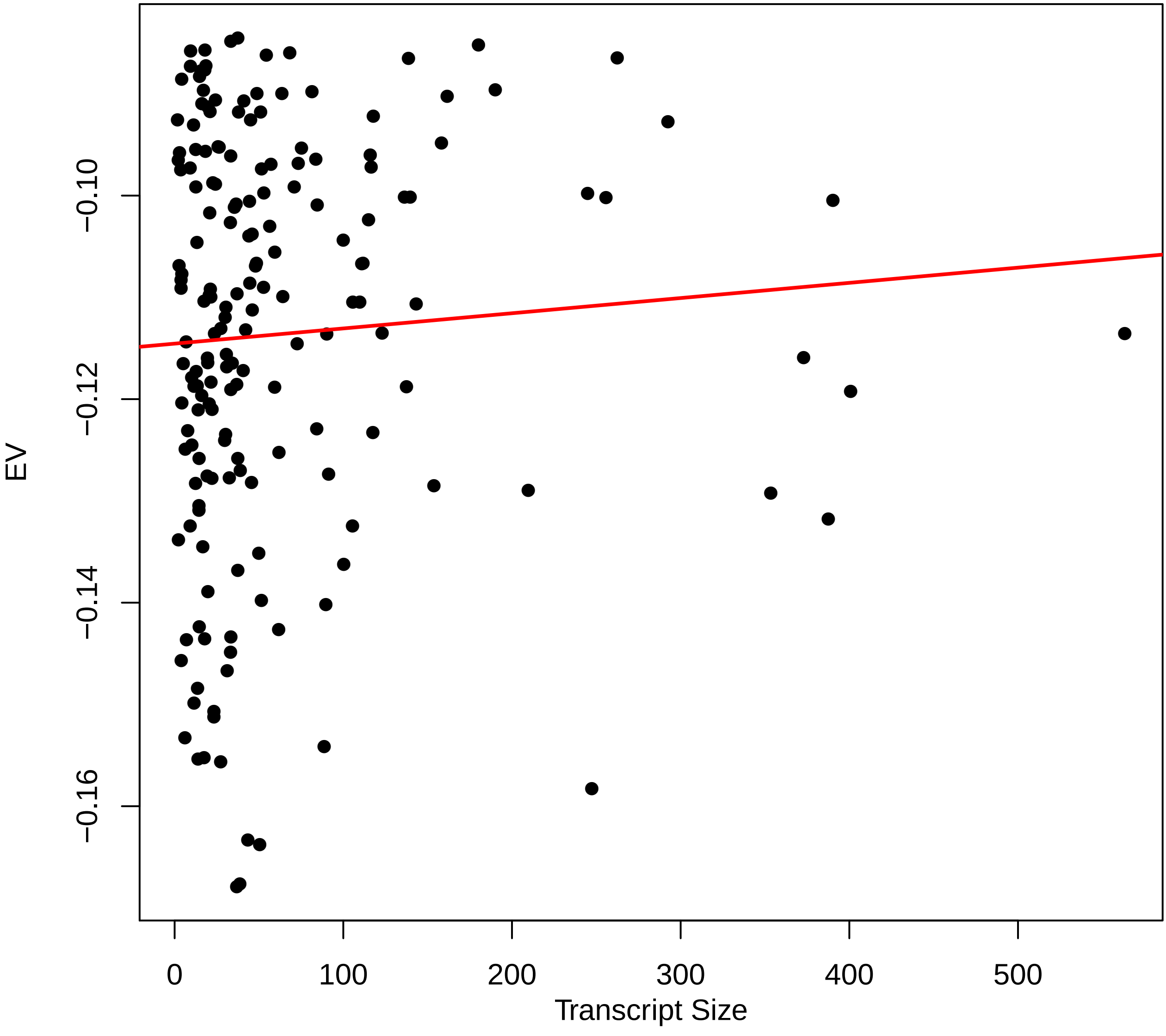


Intercept: −0.115 Slope: 1.49e−05 R2: −0.00157 Correlation: 0.0699

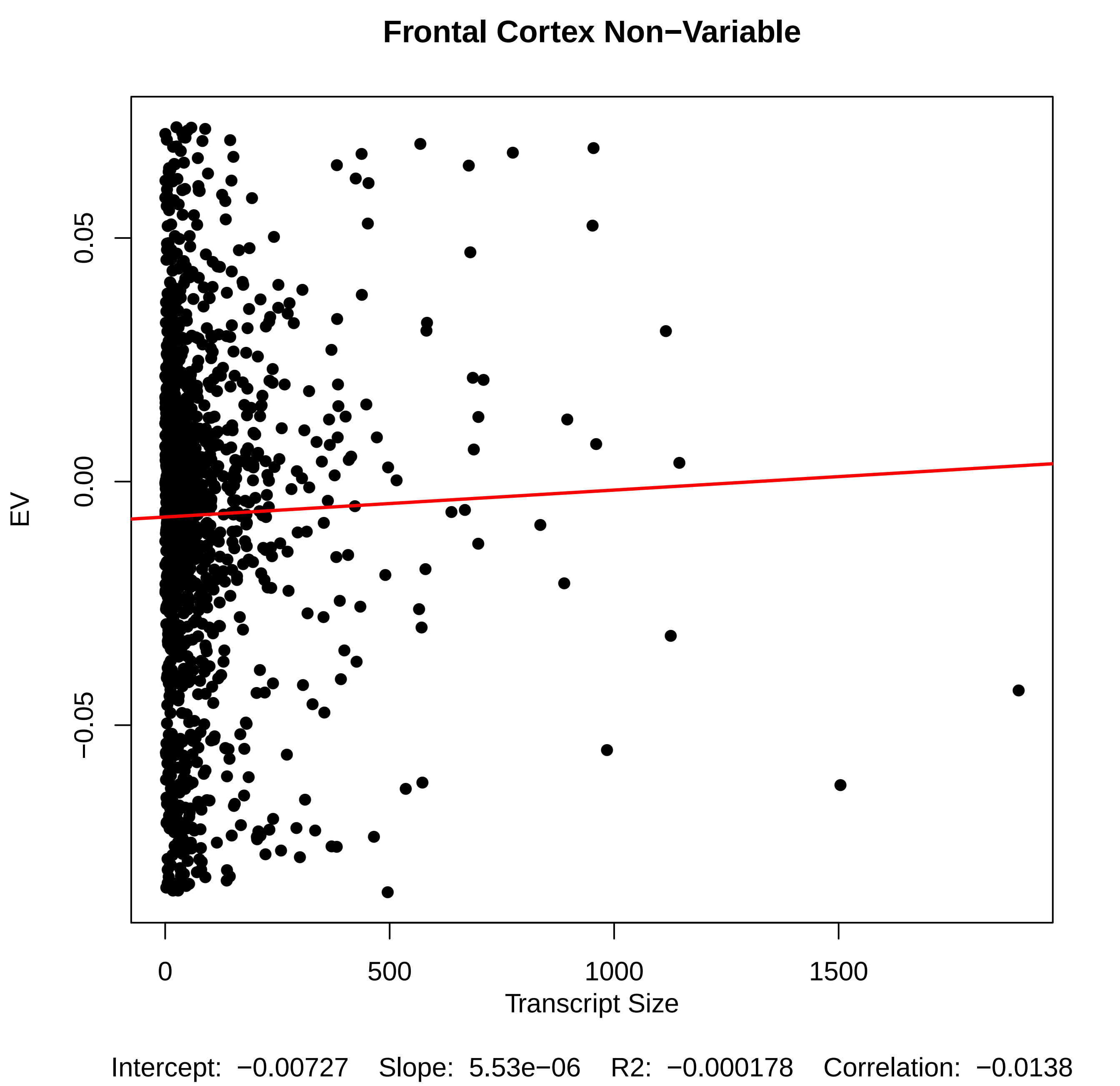
Frontal Cortex Non−Variable
EV
0.05
0.00
−0.05
0
500
1000
1500
Transcript Size
Intercept: −0.00727 Slope: 5.53e−06 R2: −0.000178 Correlation: −0.0138

# Smallest Transcript Size Linear Regression Analysis

| | Tissue | Class | Intercept | Slope | P.value |
|---|---|---|---|---|---|
| **[1,]** | Breast | Hypervariable | 0.325029359 | −1.938604e−04 | 0.33814677 |
| **[2,]** | Breast | Hypovariable | −0.181859677 | −1.289346e−05 | 0.70566715 |
| **[3,]** | Breast | Non−Variable | −0.009954538 | 2.639453e−05 | 0.07407940 |
| **[4,]** | Cerebellum | Hypervariable | 0.151690521 | −6.911264e−05 | 0.16339570 |
| **[5,]** | Cerebellum | Hypovariable | −0.104148398 | 2.934440e−06 | 0.84127610 |
| **[6,]** | Cerebellum | Non−Variable | −0.006073300 | 1.453195e−05 | 0.08455624 |
| **[7,]** | Frontal Cortex | Hypervariable | 0.173723719 | −2.998484e−05 | 0.74679880 |
| **[8,]** | Frontal Cortex | Hypovariable | −0.113632739 | 7.974851e−07 | 0.97164780 |
| **[9,]** | Frontal Cortex | Non−Variable | −0.007163614 | 6.492682e−06 | 0.49712189 |

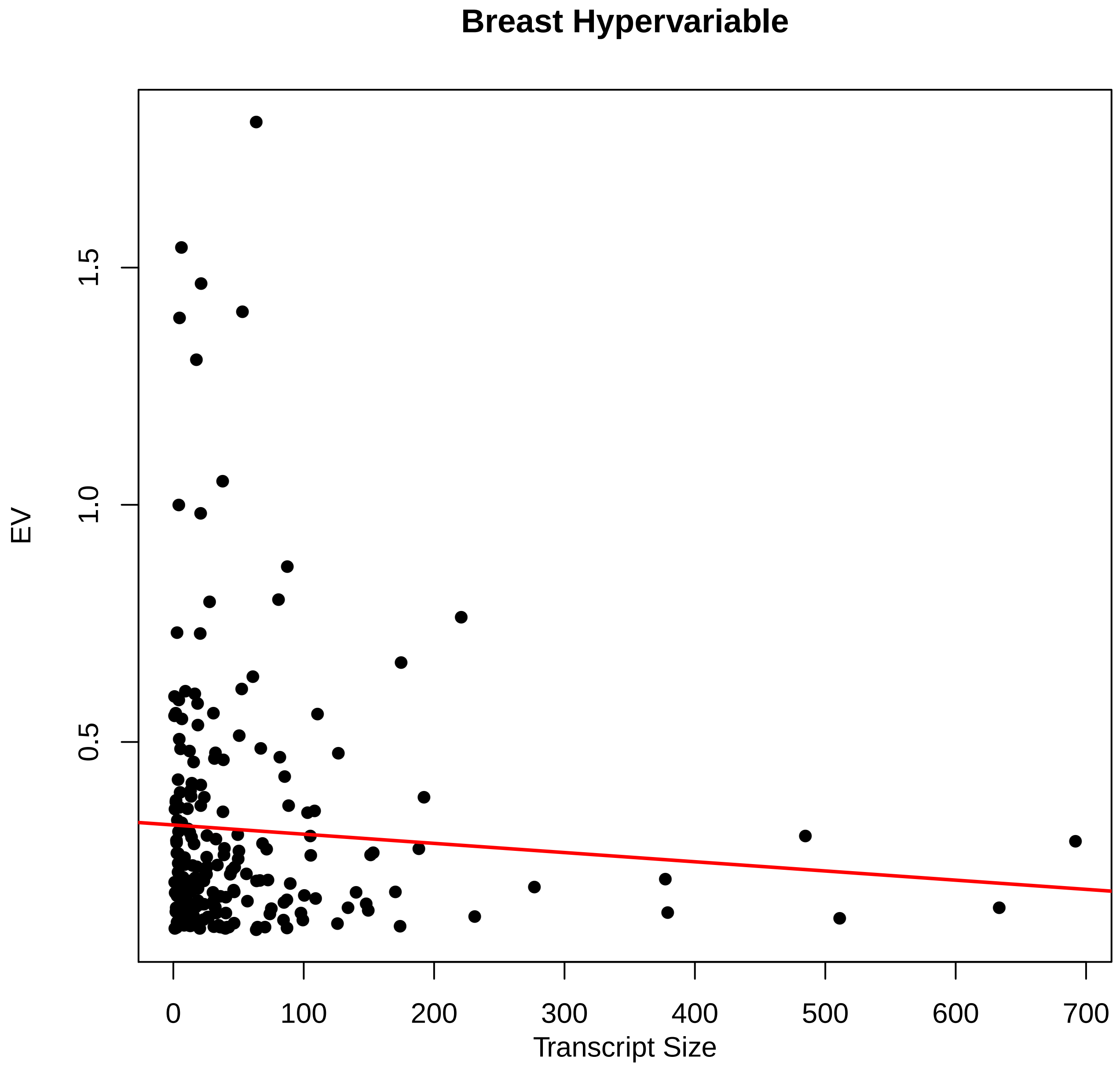


Intercept: 0.325 Slope: −0.000194 R2: −0.00038 Correlation: −0.0446

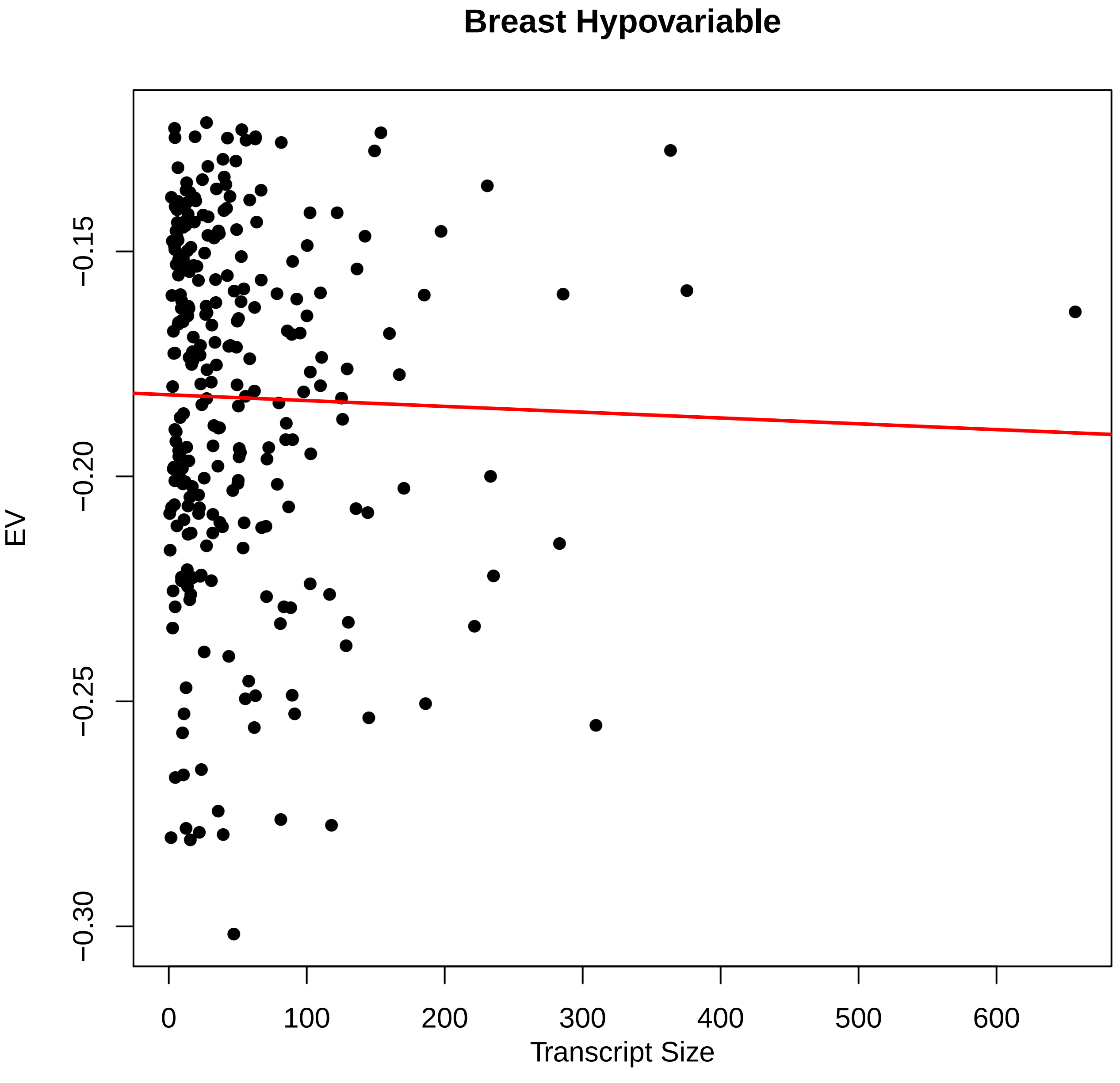


Intercept: −0.182 Slope: −1.29e−05 R2: −0.00324 Correlation: −0.0297

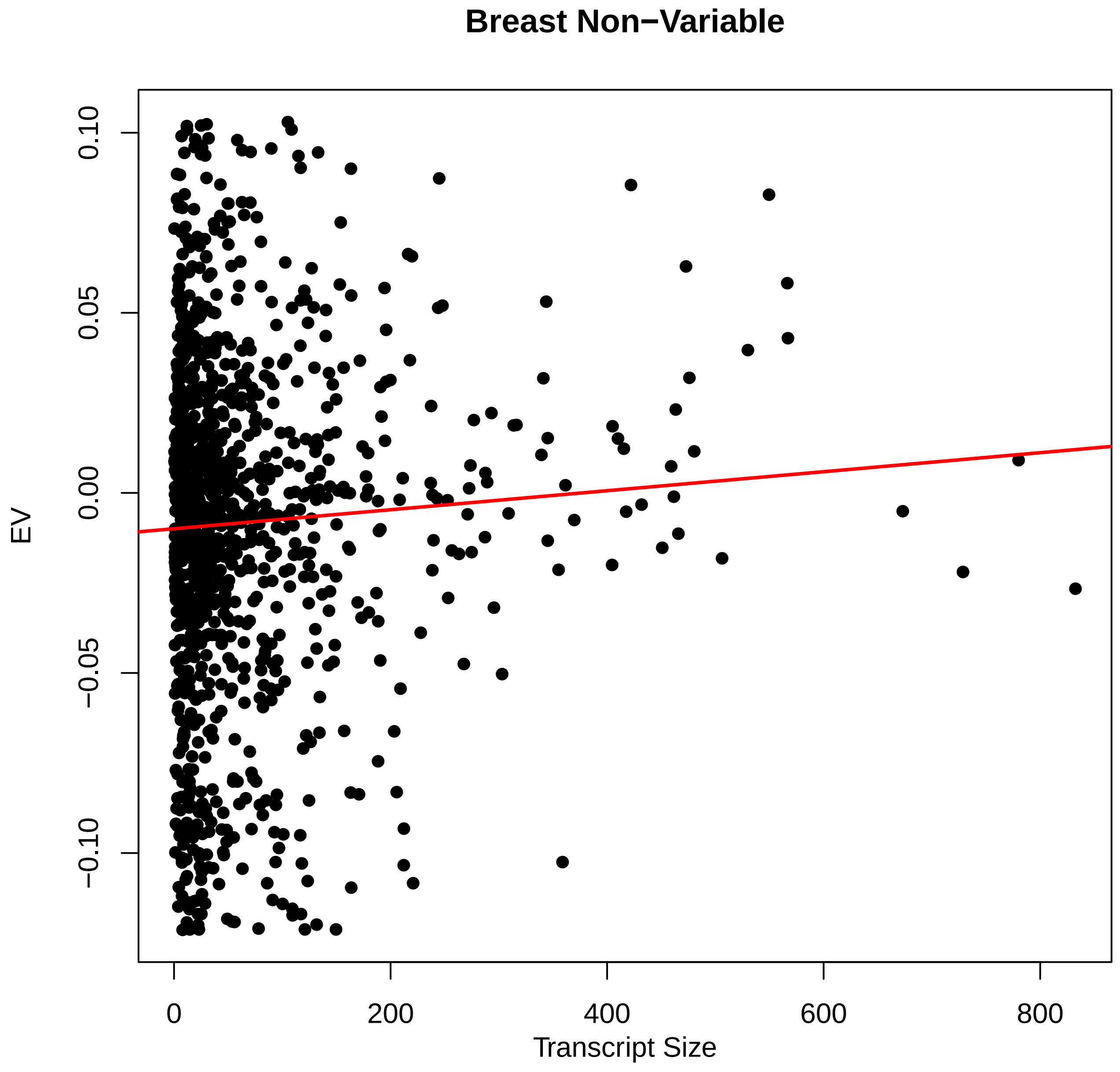


Intercept: −0.00995 Slope: 2.64e−05 R2: 0.00195 Correlation: 0.000842

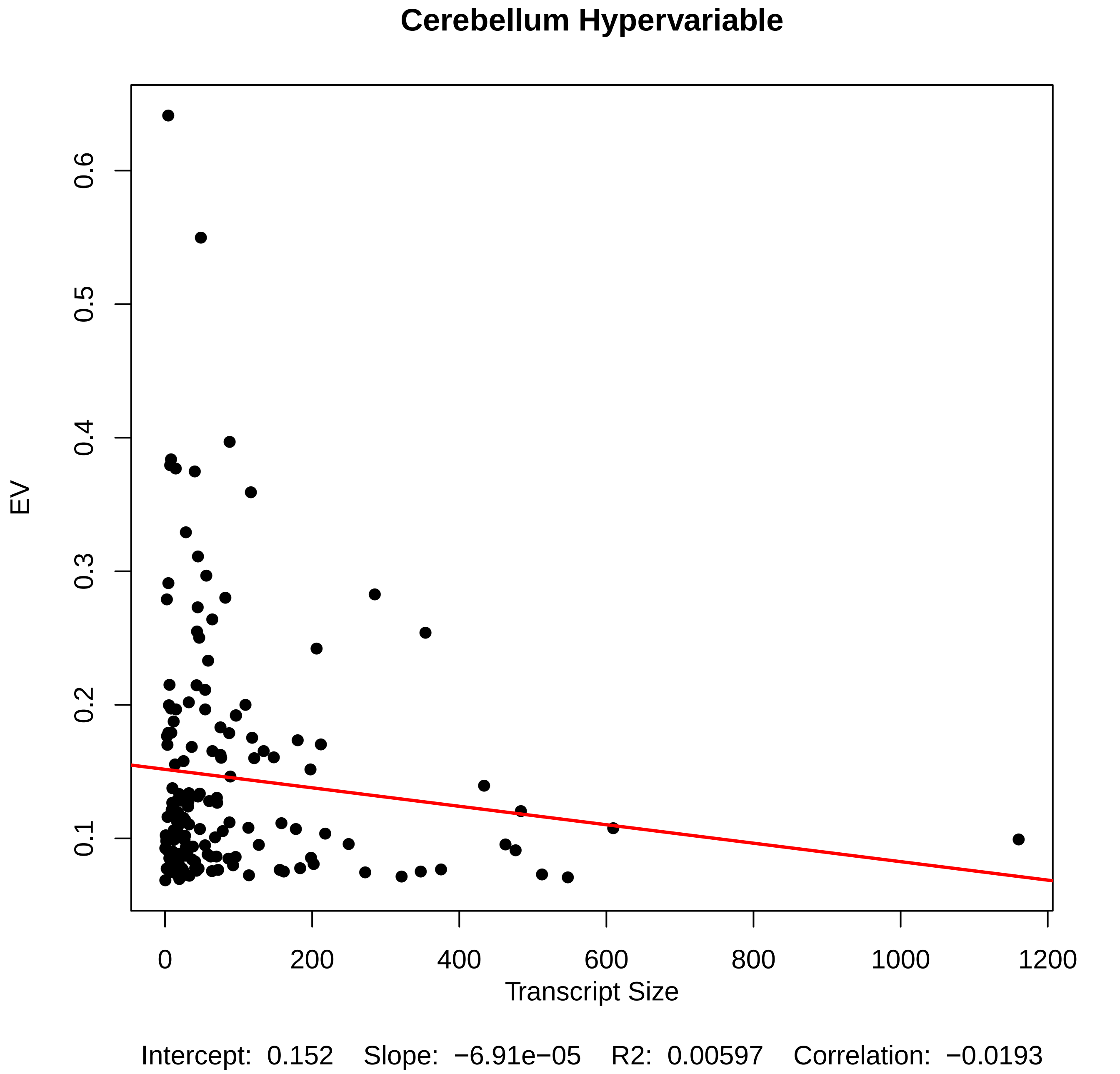
Cerebellum Hypervariable
EV
0.1
0.2
0.3
0.4
0.5
0.6
0
200
400
600
800
1000
1200
Transcript Size
Intercept: 0.152 Slope: −6.91e−05 R2: 0.00597 Correlation: −0.0193

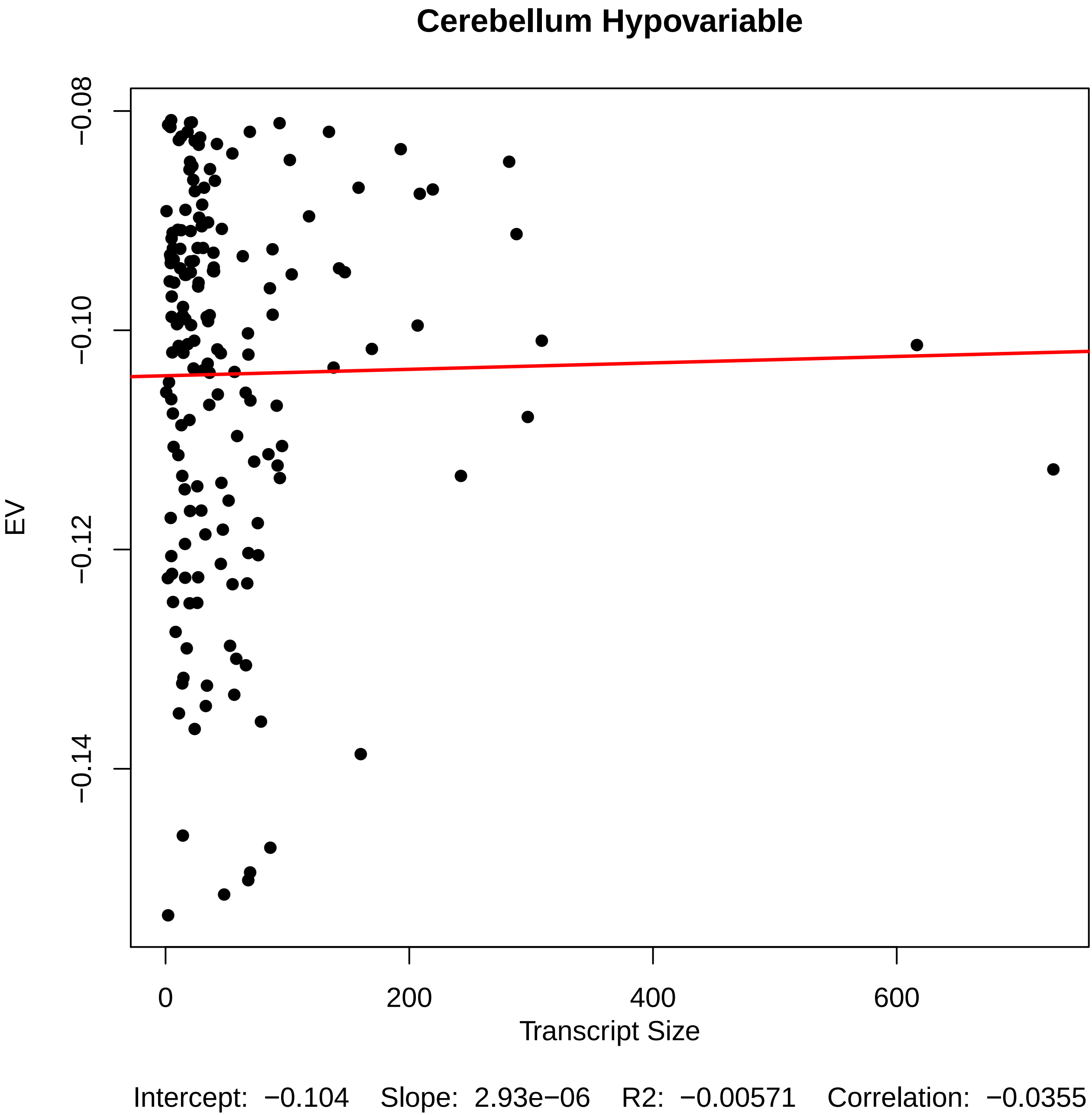

Cerebellum Hypovariable
−0.08
−0.10
−0.12
−0.14
EV
0
200
400
600
Transcript Size
Intercept: −0.104 Slope: 2.93e−06 R2: −0.00571 Correlation: −0.0355

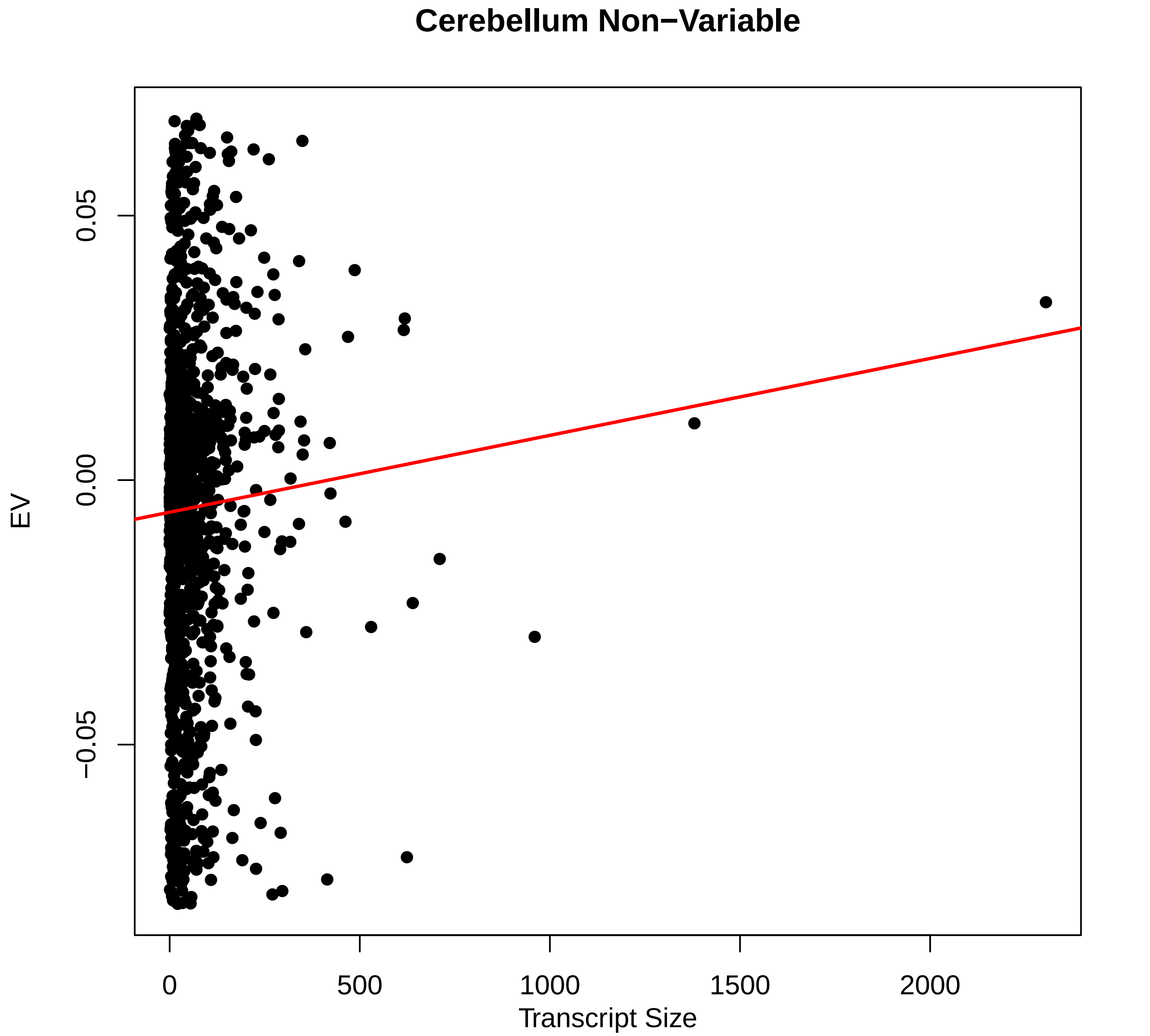


Intercept: −0.00607 Slope: 1.45e−05 R2: 0.00165 Correlation: 0.0308

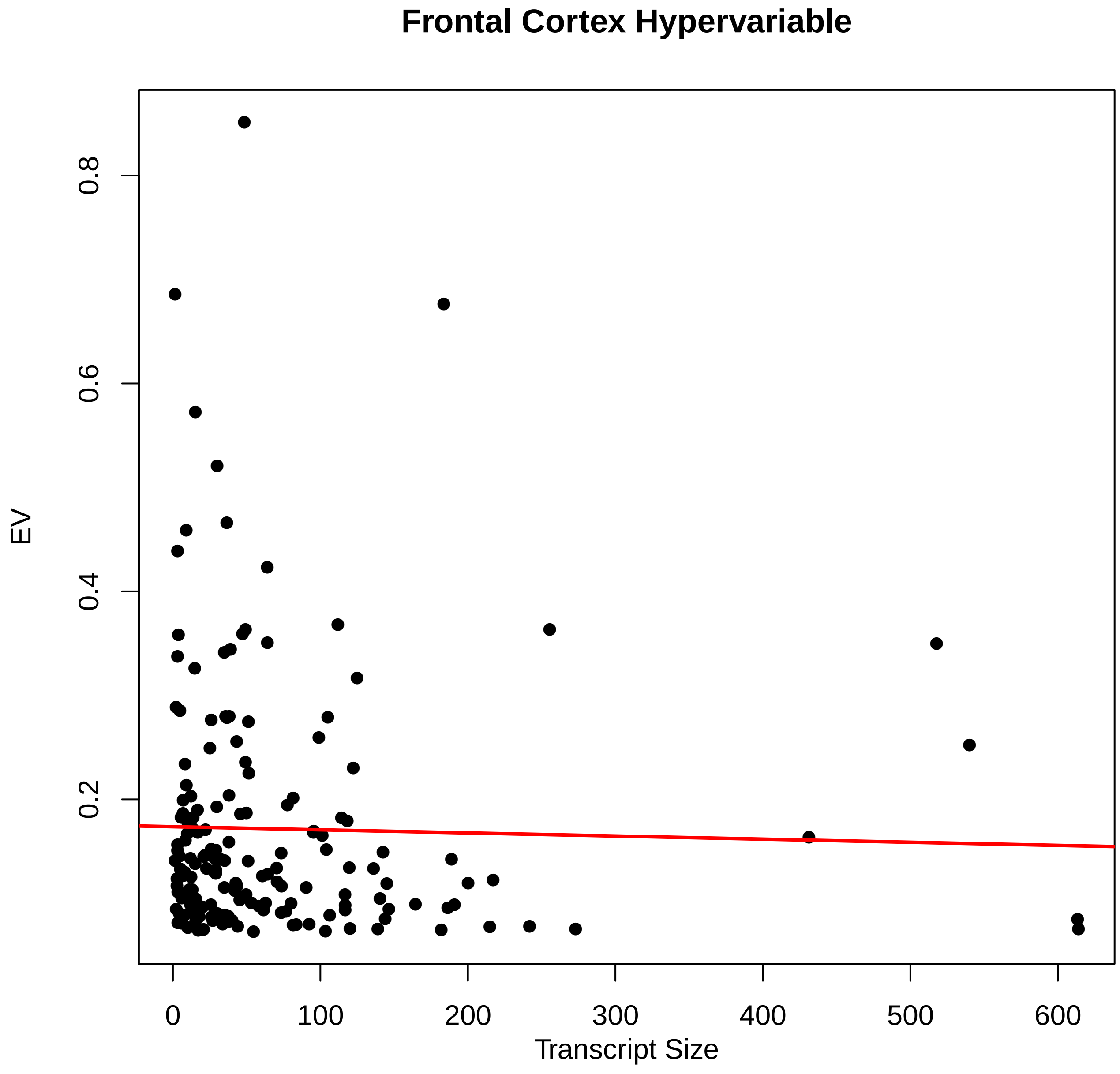


Intercept: 0.174 Slope: −3e−05 R2: −0.00511 Correlation: −0.0656

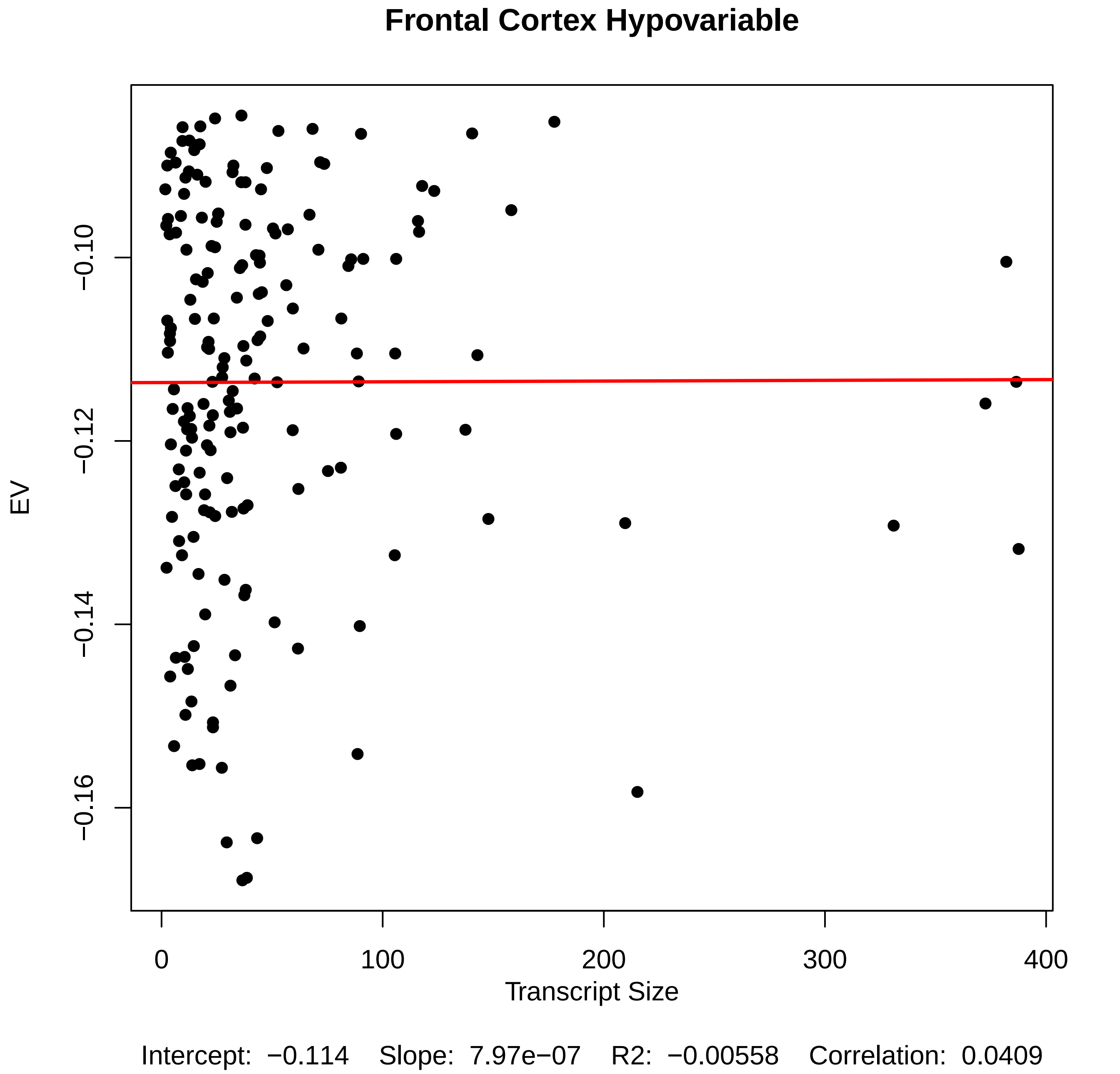

Frontal Cortex Hypovariable
EV
−0.10
−0.12
−0.14
−0.16
0
100
200
300
400
Transcript Size
Intercept: −0.114 Slope: 7.97e−07 R2: −0.00558 Correlation: 0.0409

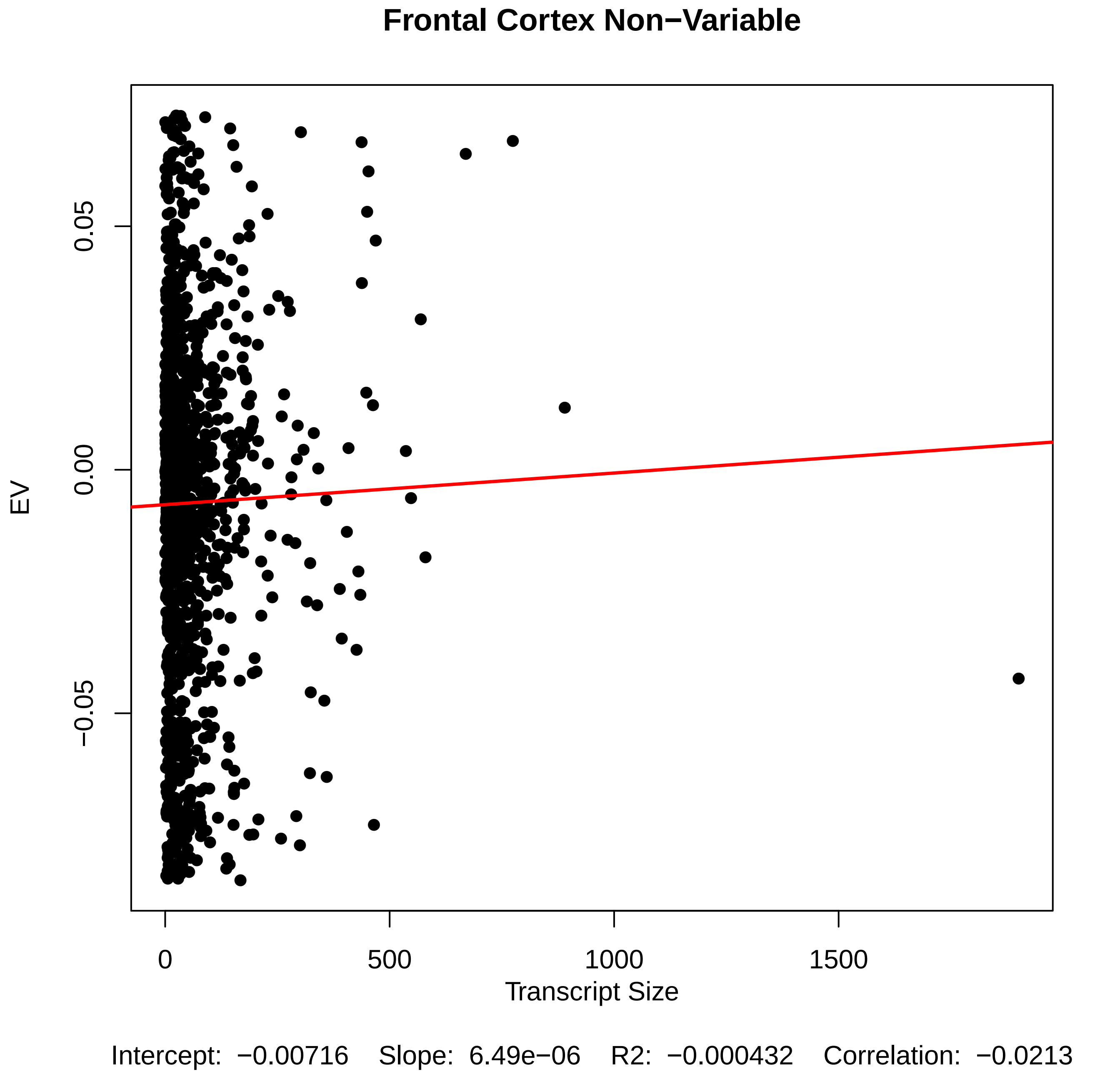

Frontal Cortex Non−Variable
EV
0.05
0.00
−0.05
0
500
1000
1500
Transcript Size
Intercept: −0.00716 Slope: 6.49e−06 R2: −0.000432 Correlation: −0.0213

# Exon Count Linear Regression Analysis

| | Tissue | Class | Intercept | Slope | P.value |
|---|---|---|---|---|---|
| **[1,]** | Breast | Hypervariable | 0.321399659 | 8.696334e−05 | 0.90054809 |
| **[2,]** | Breast | Hypovariable | −0.178363653 | 1.868211e−06 | 0.98673724 |
| **[3,]** | Breast | Non−Variable | −0.006501745 | −1.736883e−05 | 0.72300880 |
| **[4,]** | Cerebellum | Hypervariable | 0.151481544 | −5.539178e−04 | 0.06694287 |
| **[5,]** | Cerebellum | Hypovariable | −0.104869704 | −4.099122e−05 | 0.55248606 |
| **[6,]** | Cerebellum | Non−Variable | −0.004678161 | 4.606558e−06 | 0.88551046 |
| **[7,]** | Frontal Cortex | Hypervariable | 0.174125652 | −6.490396e−04 | 0.08671930 |
| **[8,]** | Frontal Cortex | Hypovariable | −0.111961012 | −4.020745e−05 | 0.56635617 |
| **[9,]** | Frontal Cortex | Non−Variable | −0.004379761 | −2.219381e−05 | 0.51065656 |

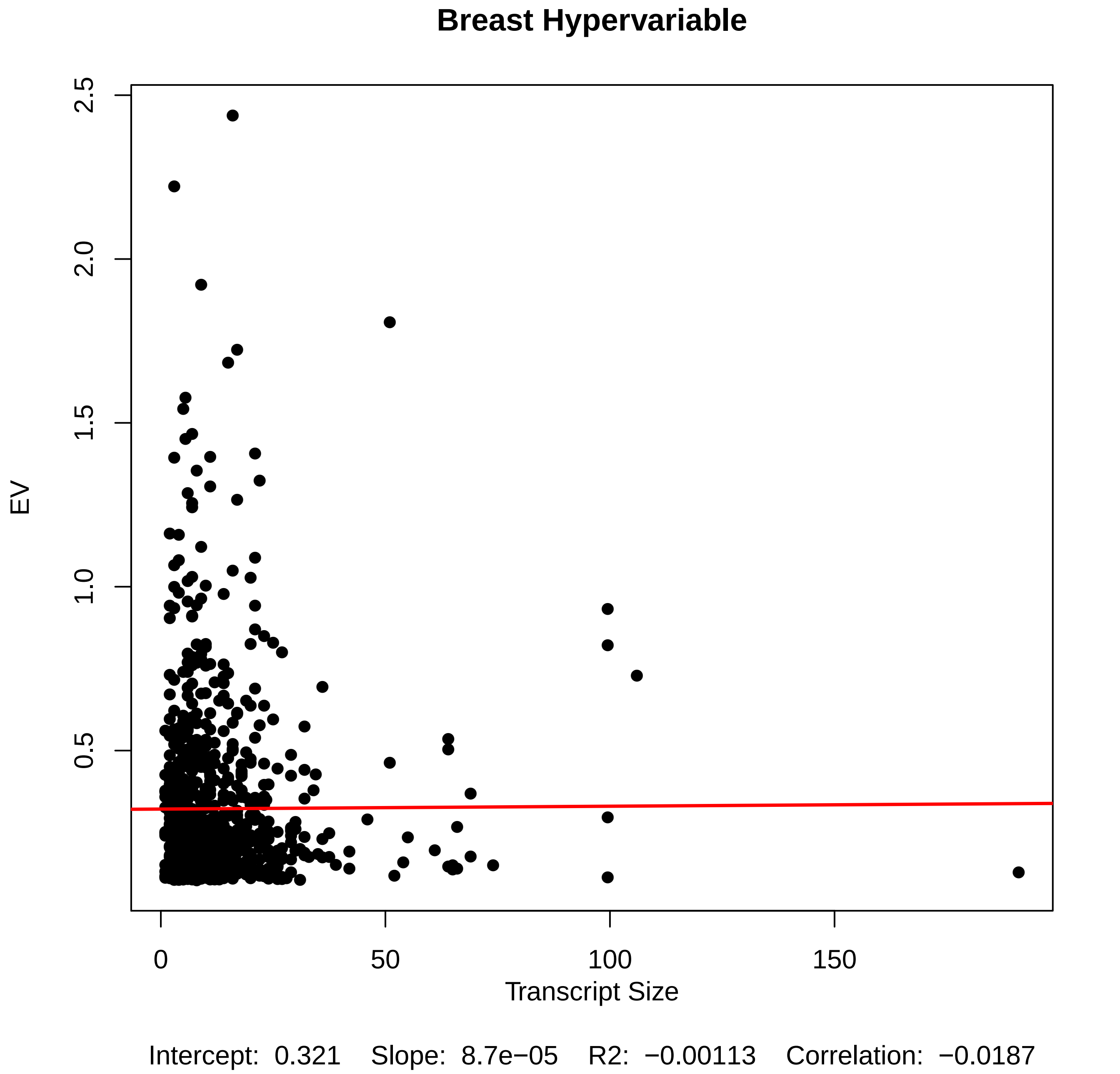
Breast Hypervariable
EV
Transcript Size
0
50
100
150
0.5
1.0
1.5
2.0
2.5
Intercept: 0.321 Slope: 8.7e−05 R2: −0.00113 Correlation: −0.0187

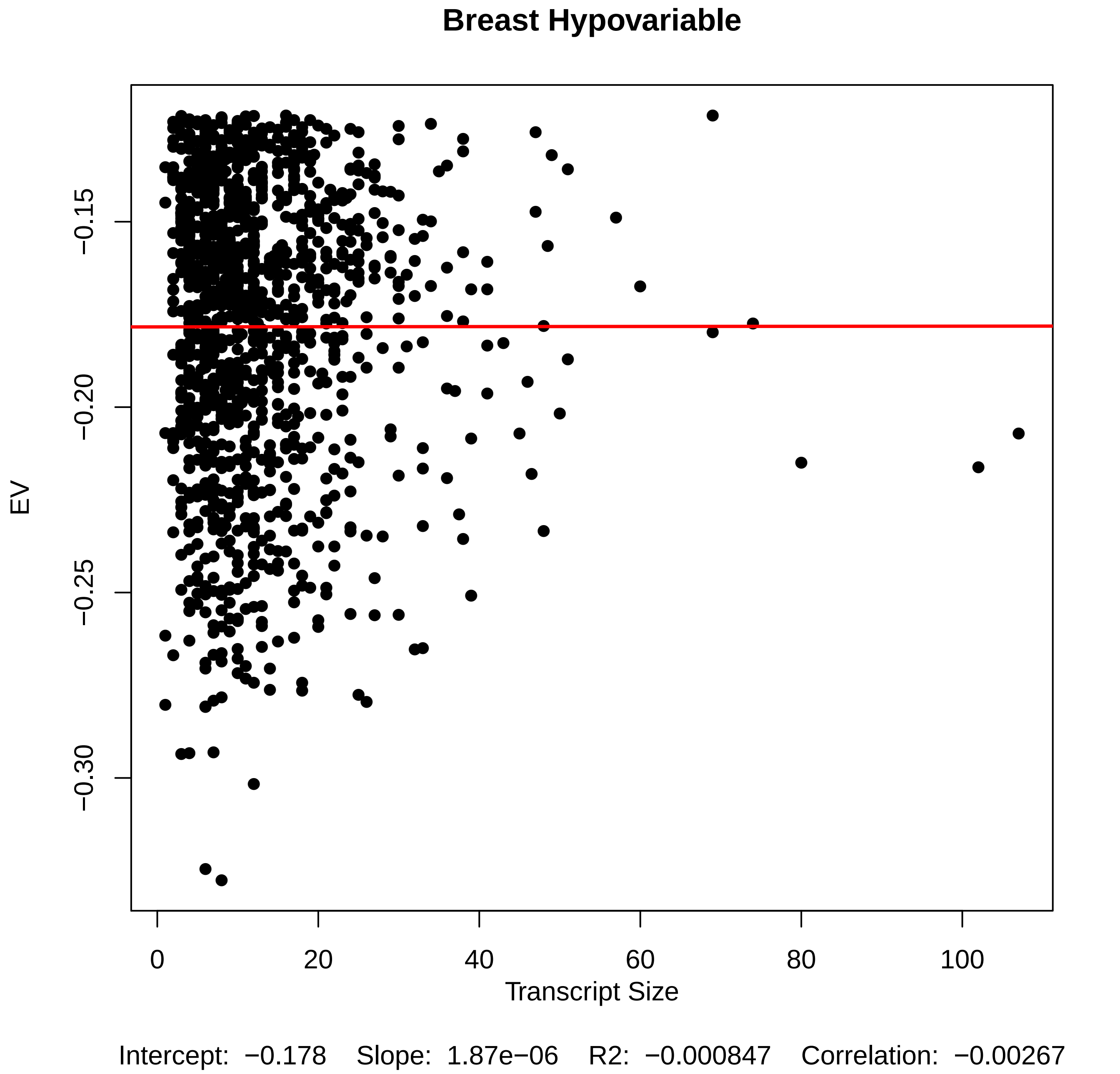
Breast Hypovariable
EV
−0.15
−0.20
−0.25
−0.30
0
20
40
60
80
100
Transcript Size
Intercept: −0.178 Slope: 1.87e−06 R2: −0.000847 Correlation: −0.00267

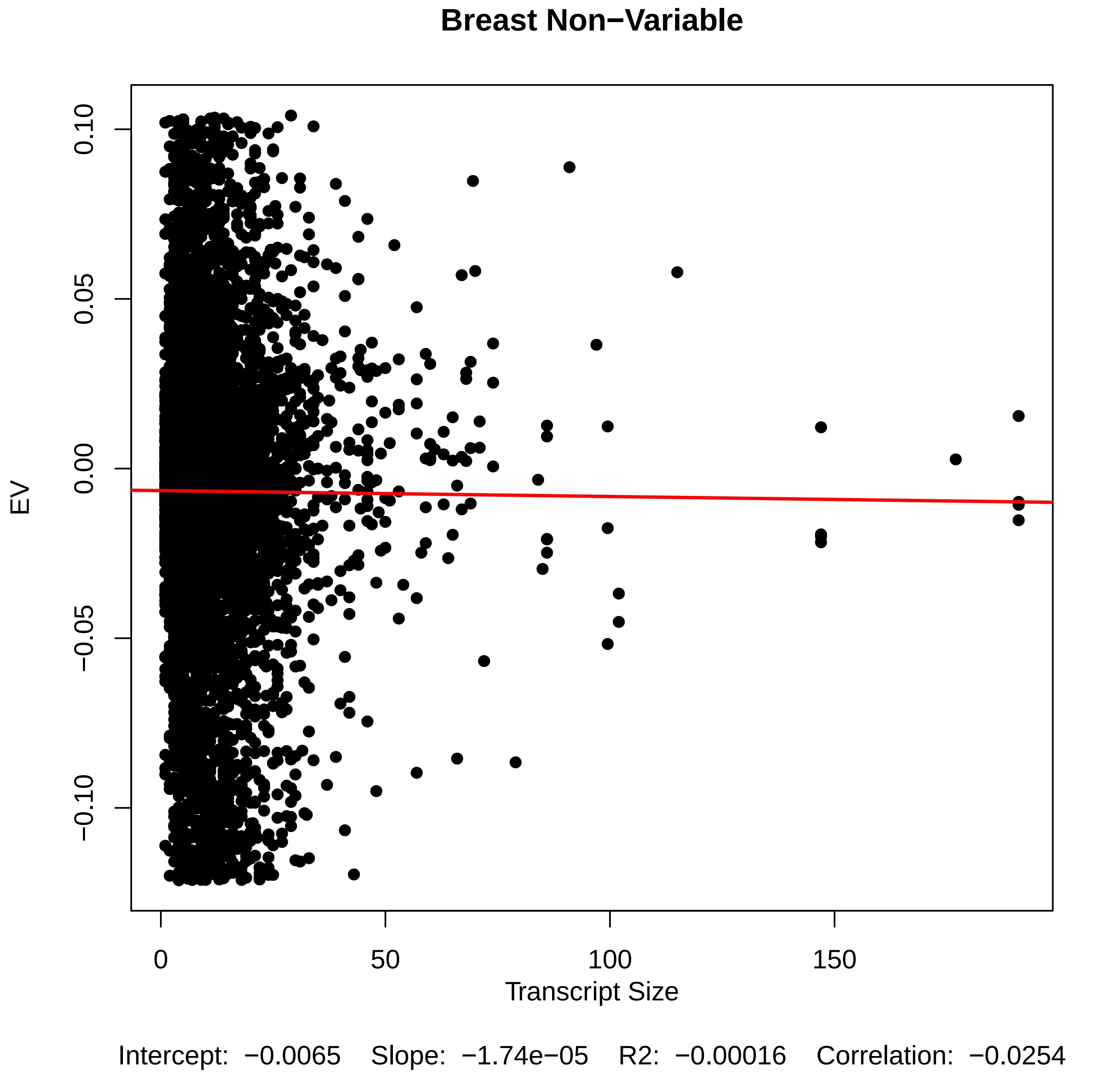
Breast Non−Variable
EV
0.10
0.05
0.00
−0.05
−0.10
0
50
100
150
Transcript Size
Intercept: −0.0065 Slope: −1.74e−05 R2: −0.00016 Correlation: −0.0254

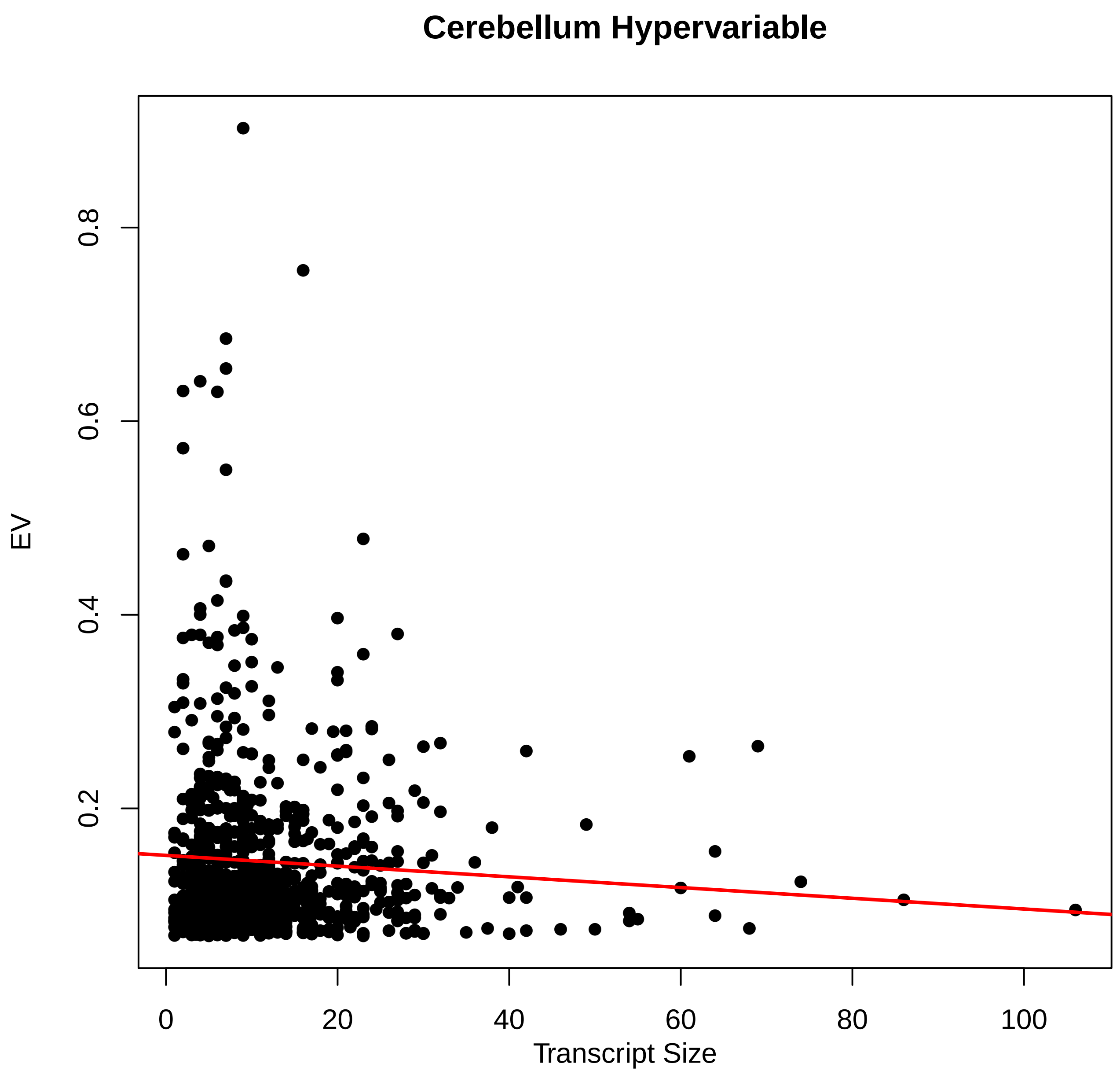


Intercept: 0.151 Slope: −0.000554 R2: 0.00299 Correlation: −0.0427

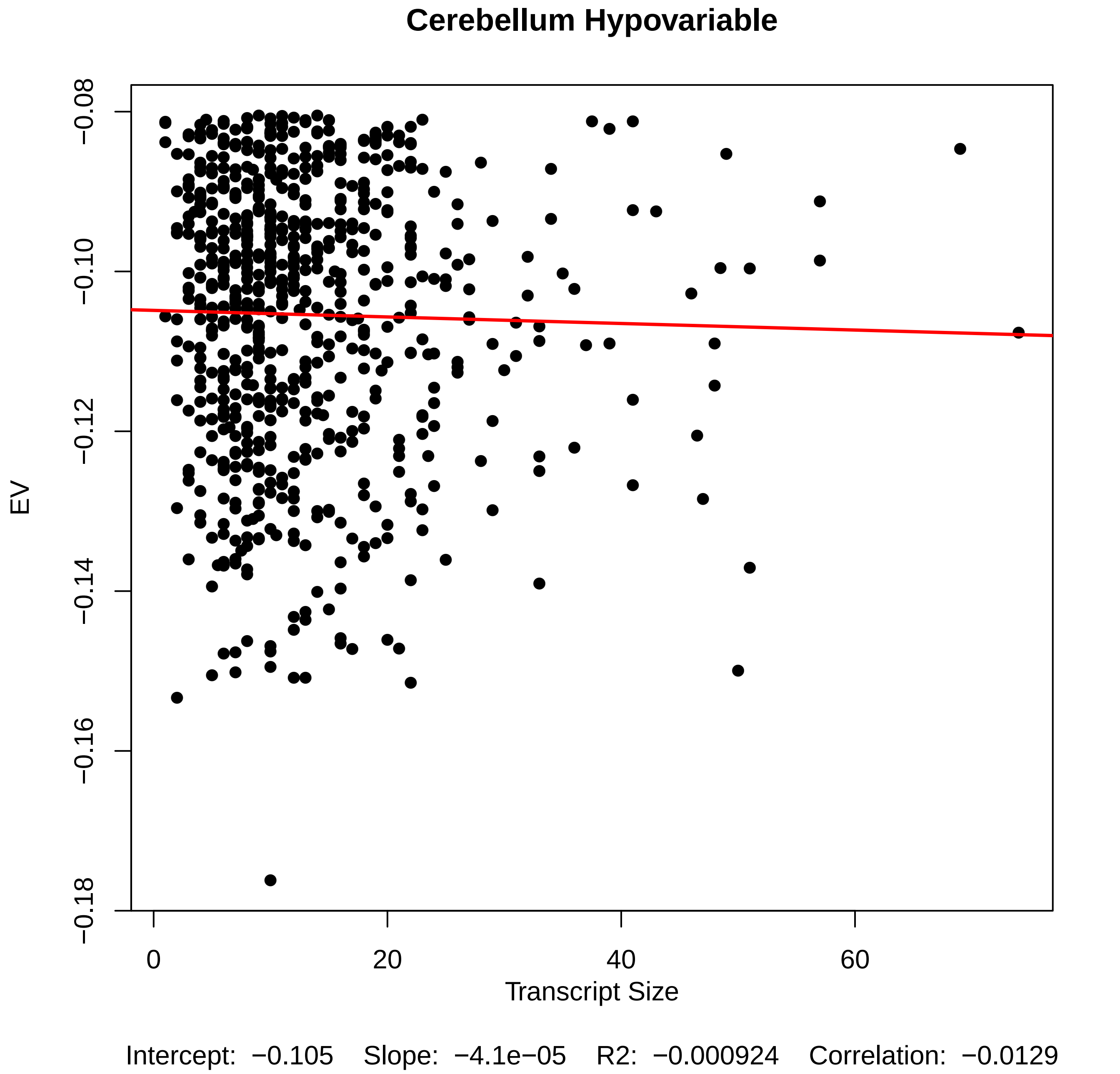
Cerebellum Hypovariable
−0.08
−0.10
−0.12
−0.14
−0.16
−0.18
EV
0
20
40
60
Transcript Size
Intercept: −0.105 Slope: −4.1e−05 R2: −0.000924 Correlation: −0.0129

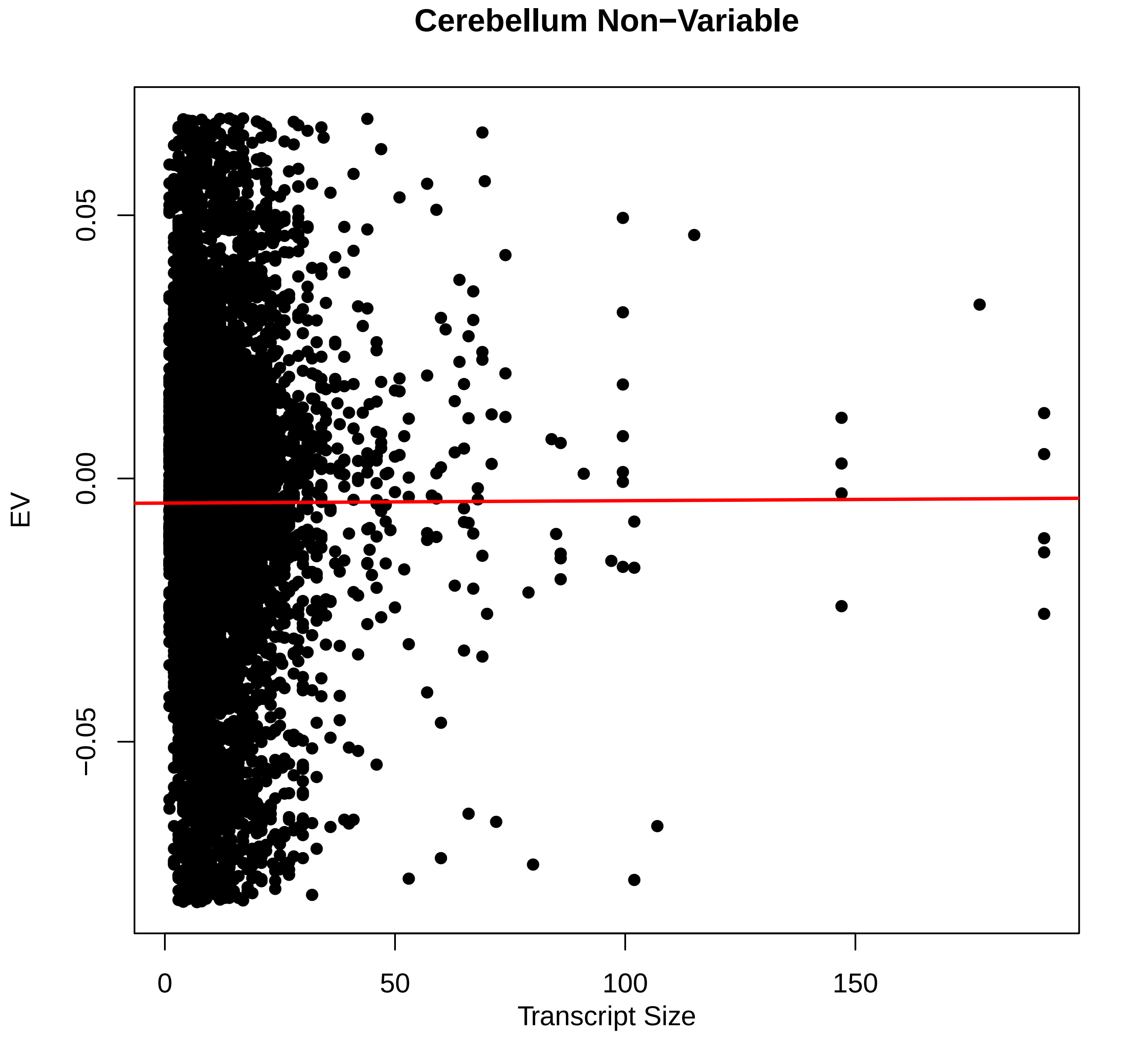


Intercept: −0.00468 Slope: 4.61e−06 R2: −0.000163 Correlation: −0.0162

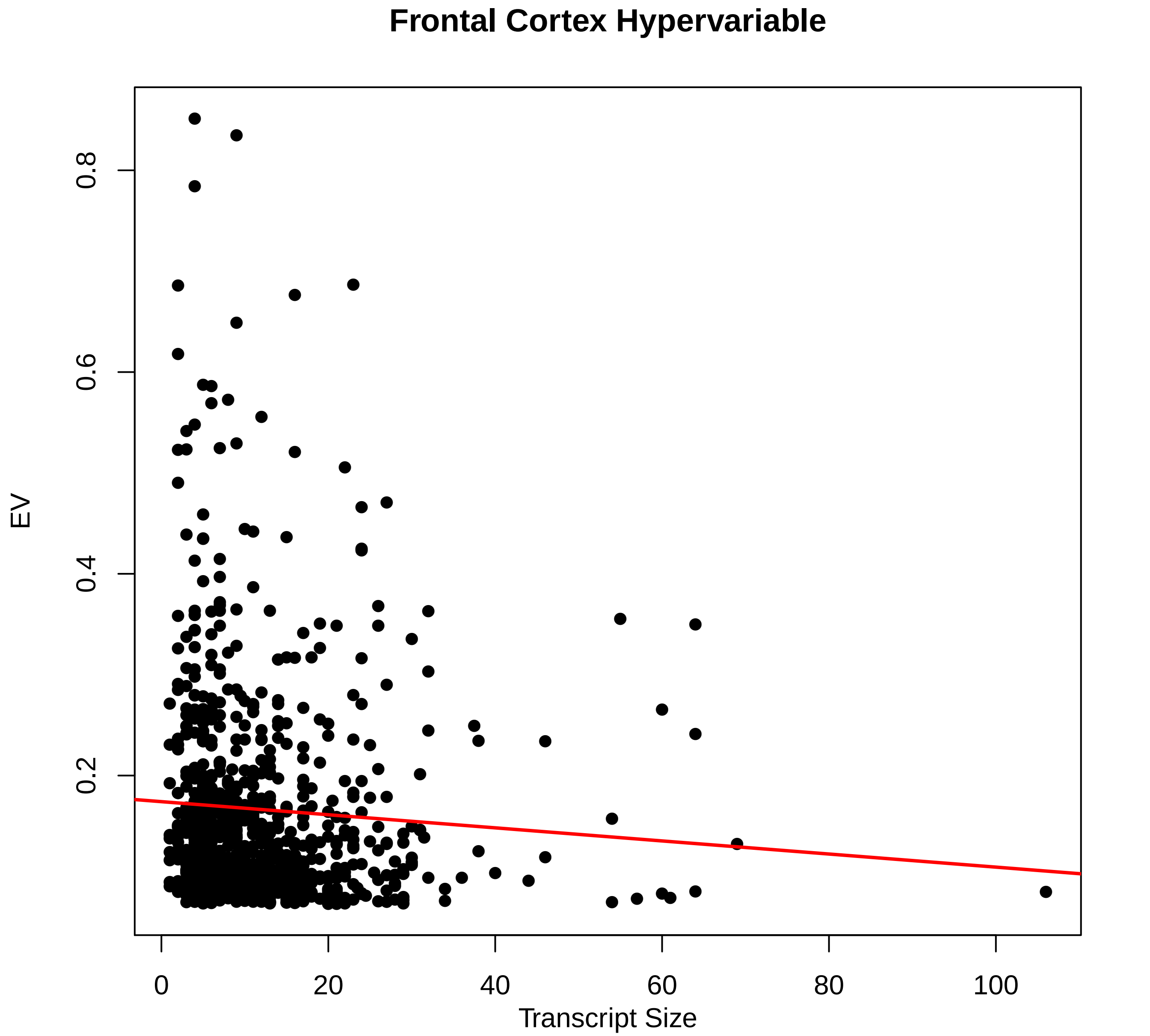


Intercept: 0.174 Slope: −0.000649 R2: 0.0024 Correlation: −0.0818

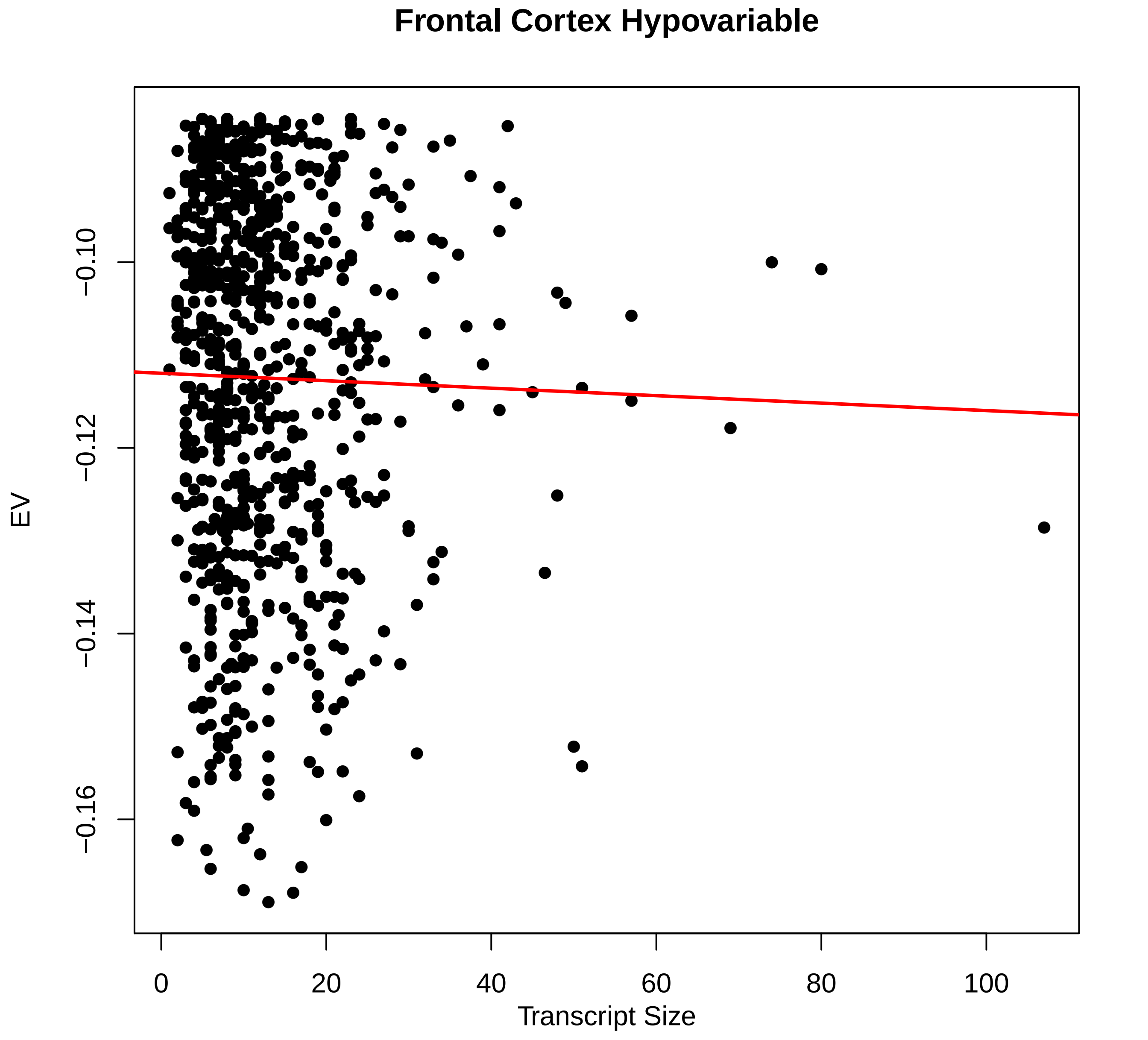


Intercept: −0.112 Slope: −4.02e−05 R2: −0.000843 Correlation: −0.0202

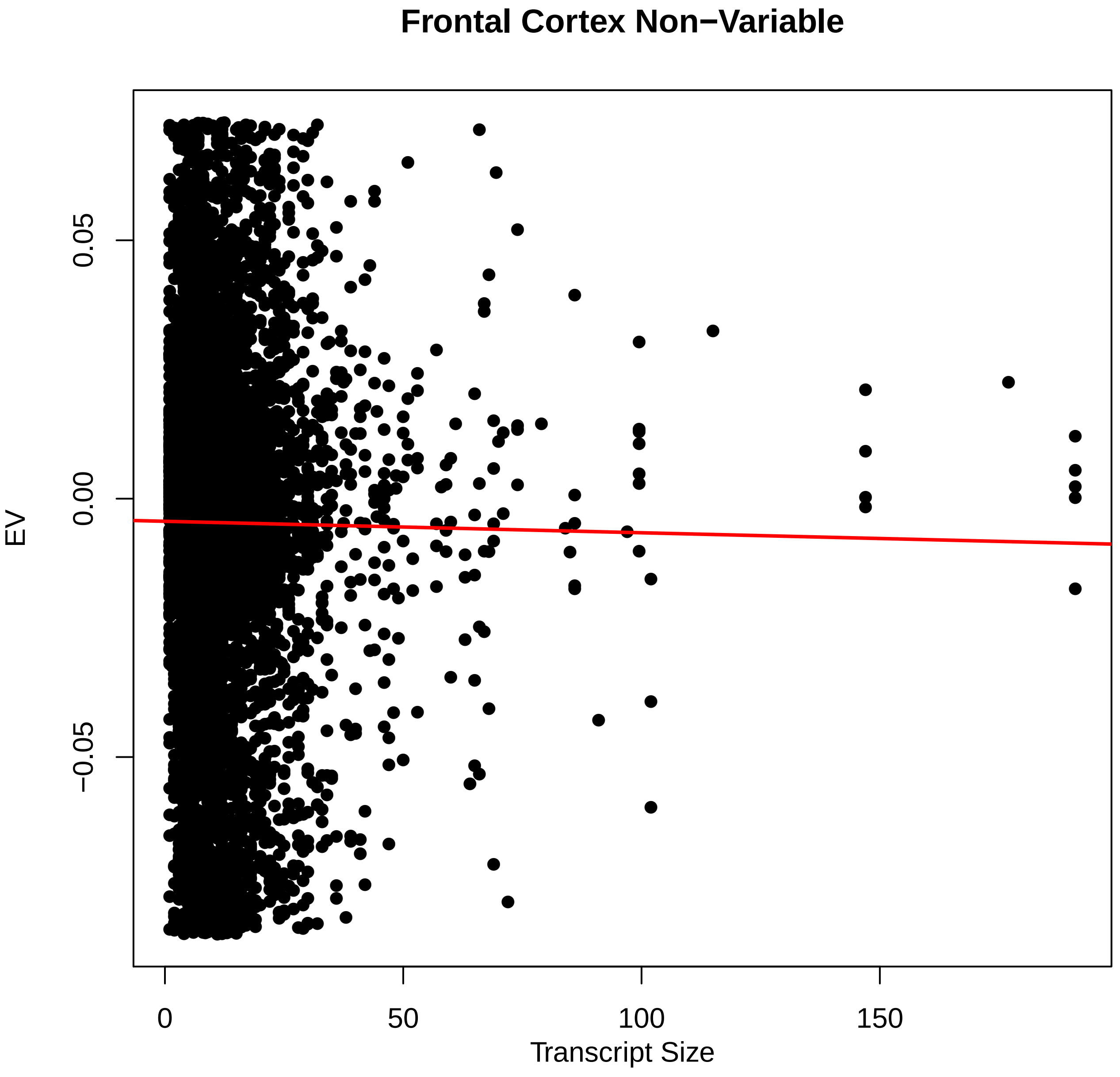


Intercept: −0.00438 Slope: −2.22e−05 R2: −9.6e−05 Correlation: −0.0236

# Sequence Length Linear Regression Analysis

| | Tissue | Class | Intercept | Slope | P.value |
|---|---|---|---|---|---|
| [1,] | Breast | Hypervariable | 0.356761881 | 1.138788e−06 | 0.785302220 |
| [2,] | Breast | Hypovariable | −0.177899245 | 4.242505e−07 | 0.448570934 |
| [3,] | Breast | Non−Variable | −0.006862151 | 7.455425e−08 | 0.754167152 |
| [4,] | Cerebellum | Hypervariable | 0.144741114 | 1.211111e−06 | 0.481605329 |
| [5,] | Cerebellum | Hypovariable | −0.102535192 | −4.813790e−07 | 0.203487408 |
| [6,] | Cerebellum | Non−Variable | −0.003441372 | −4.142047e−07 | 0.006519796 |
| [7,] | Frontal Cortex | Hypervariable | 0.171161101 | −5.706471e−06 | 0.001809231 |
| [8,] | Frontal Cortex | Hypovariable | −0.109123639 | −7.324830e−07 | 0.066204659 |
| [9,] | Frontal Cortex | Non−Variable | −0.002957930 | −4.178332e−07 | 0.009309854 |

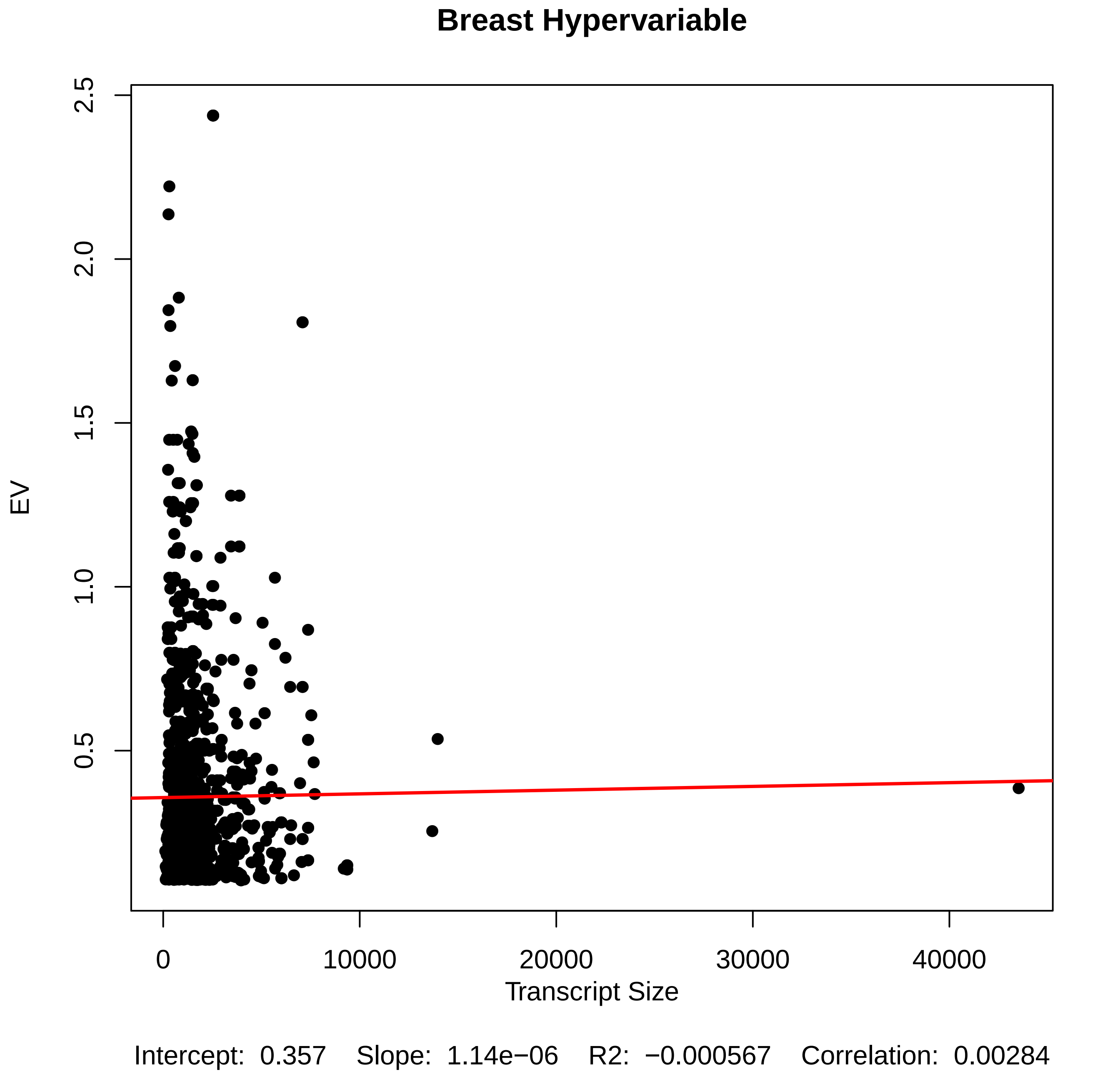

Breast Hypervariable
EV
2.5
2.0
1.5
1.0
0.5
0
10000
20000
30000
40000
Transcript Size
Intercept: 0.357 Slope: 1.14e−06 R2: −0.000567 Correlation: 0.00284

Breast Hypovariable

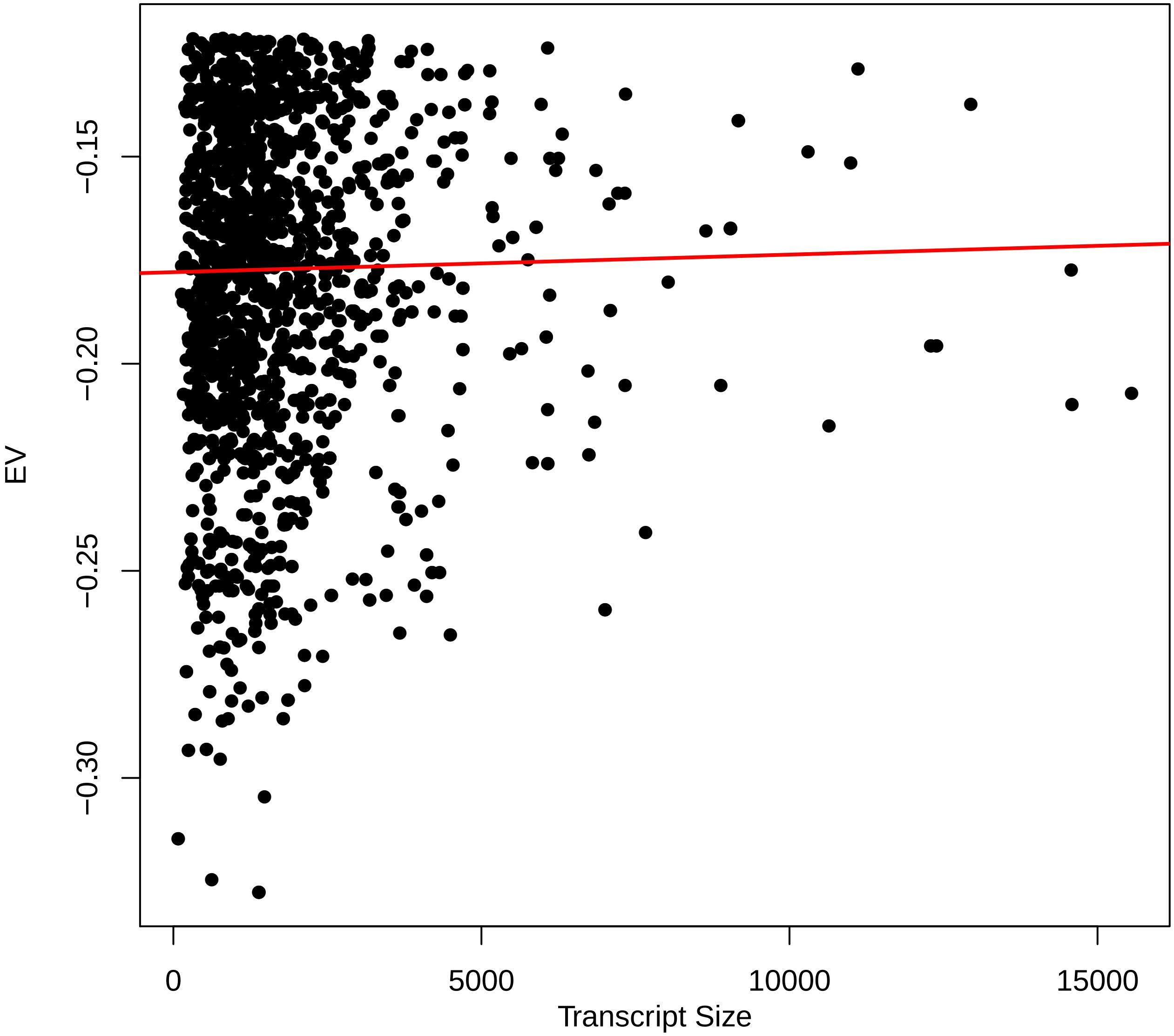


Intercept: −0.178 Slope: 4.24e−07 R2: −0.000212 Correlation: 0.0171

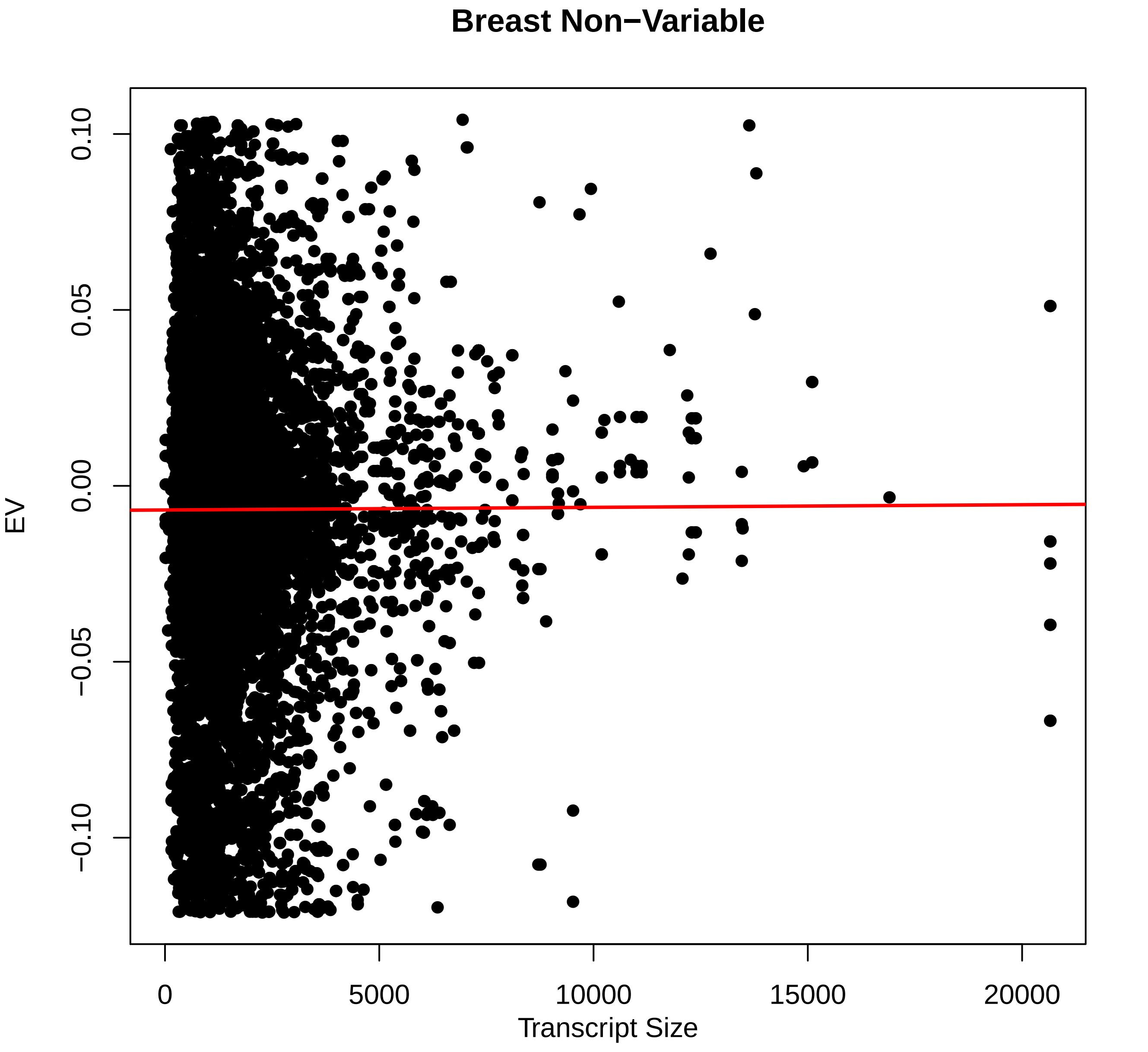


Intercept: −0.00686 Slope: 7.46e−08 R2: −7.35e−05 Correlation: −0.0104

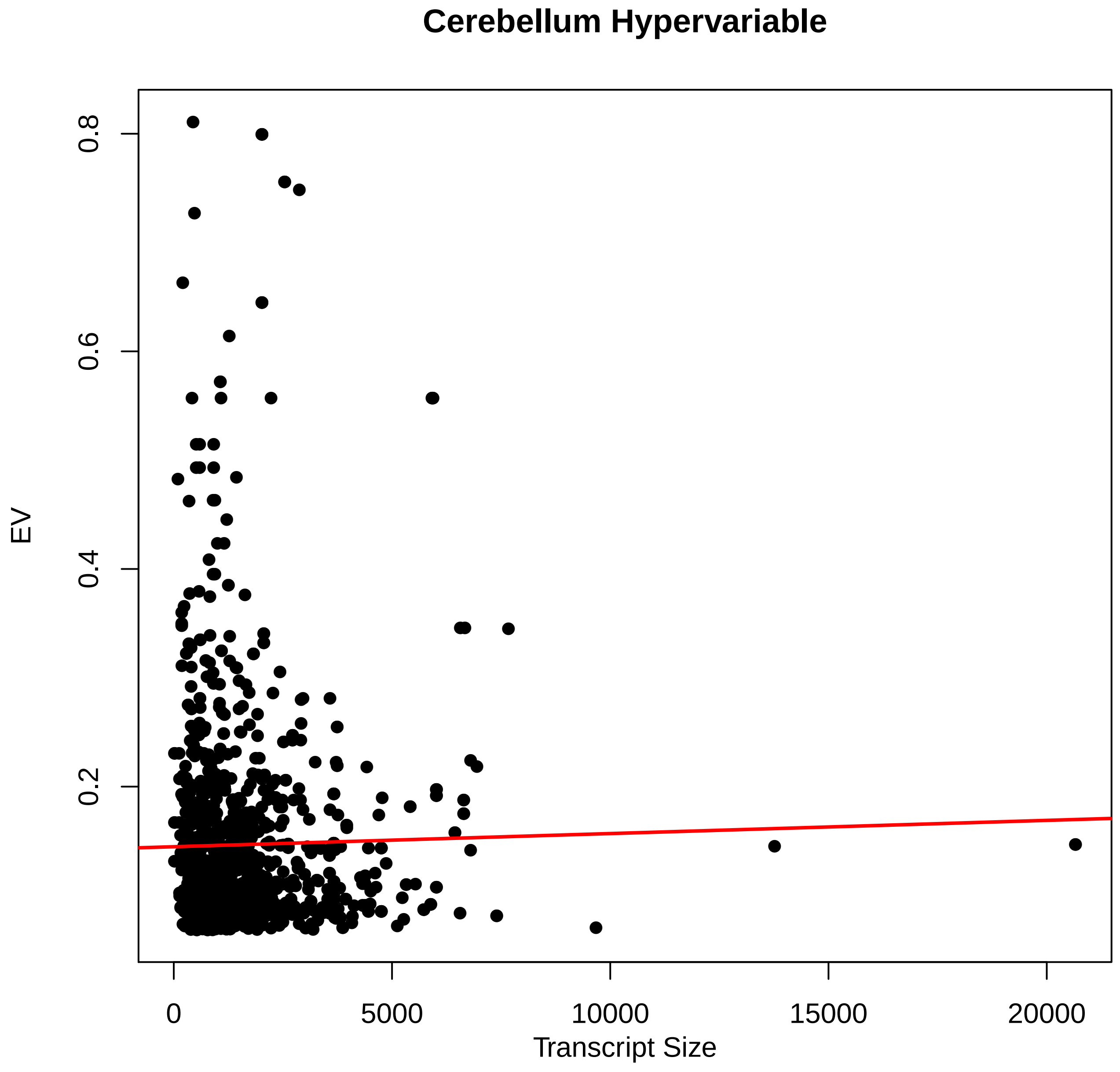


Intercept: 0.145 Slope: 1.21e−06 $R2$: −0.000361 Correlation: −0.00502

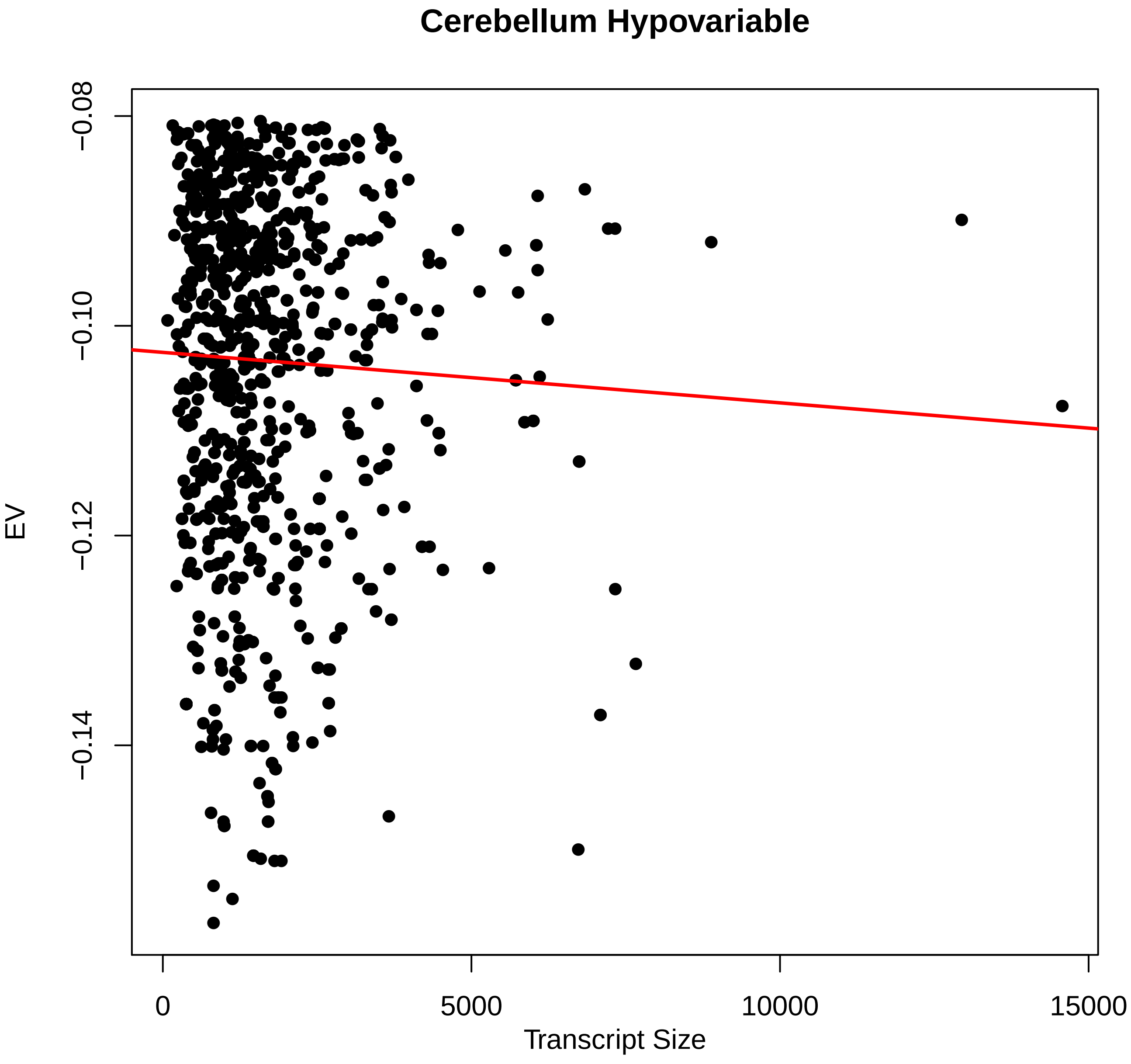


Intercept: −0.103 Slope: −4.81e−07 R2: 0.000556 Correlation: −0.0308

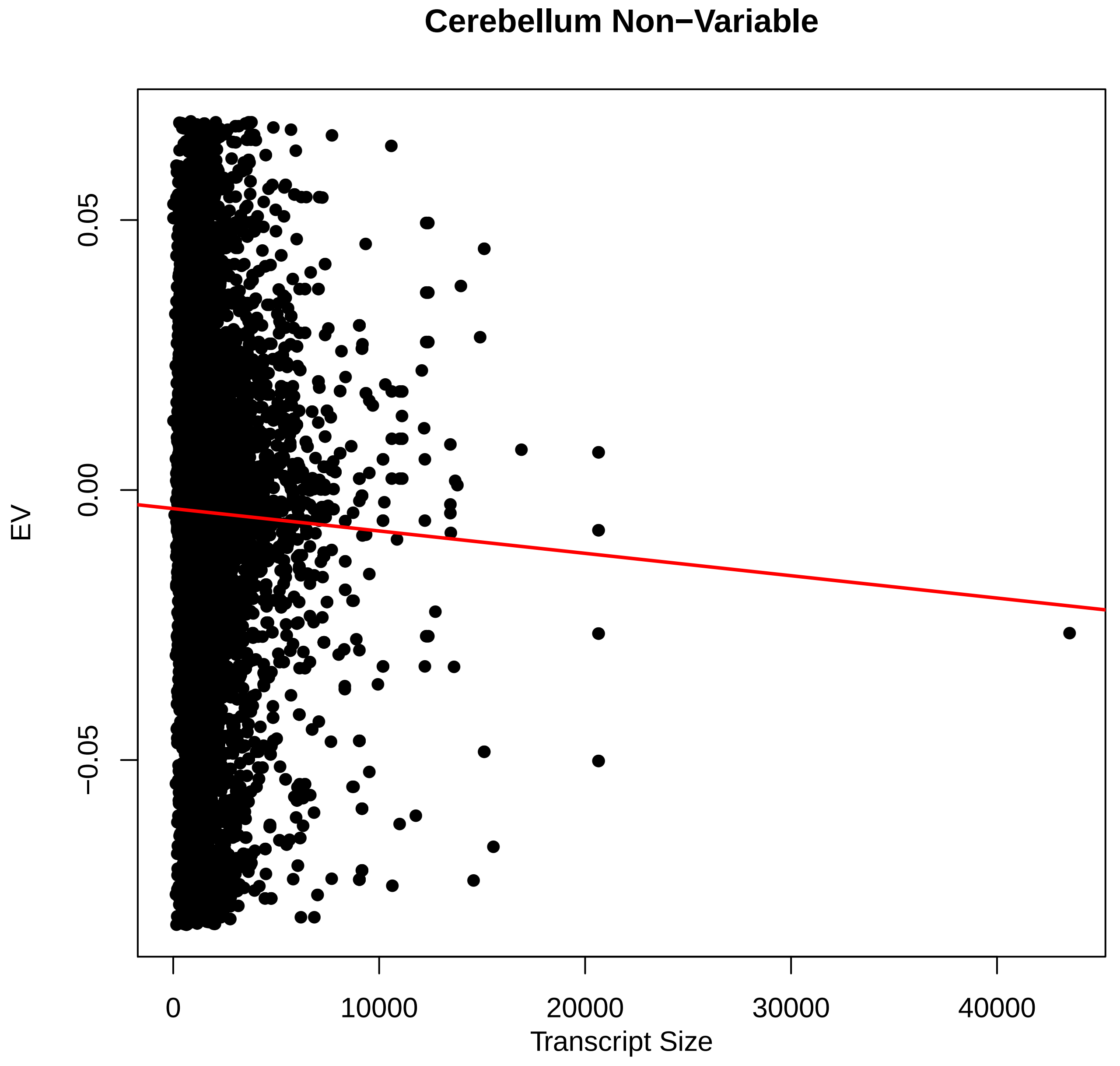


Intercept: −0.00344  Slope: −4.14e−07  R2: 0.000477  Correlation: −0.021

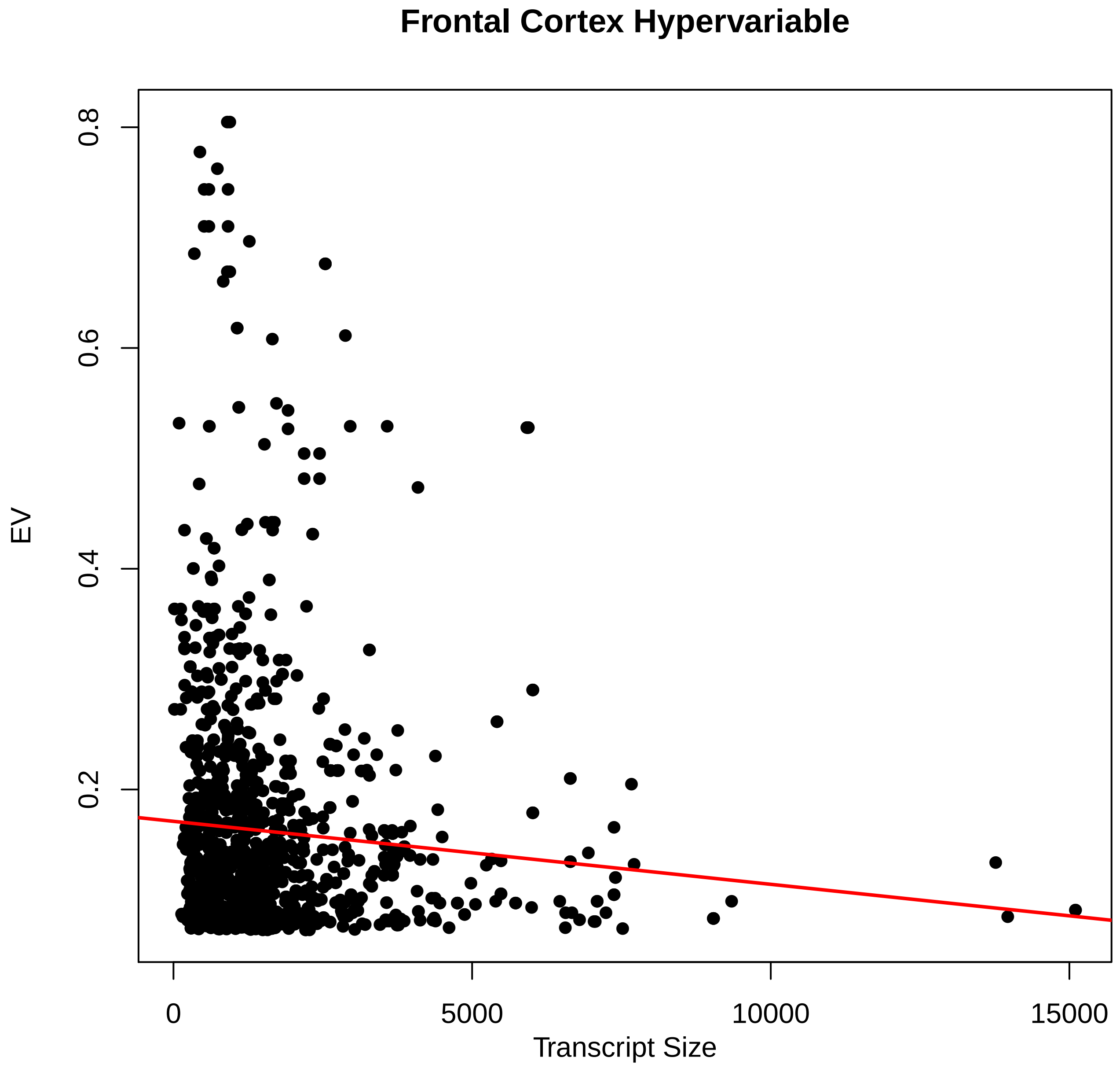


Intercept: 0.171 Slope: −5.71e−06 R2: 0.00572 Correlation: −0.0925

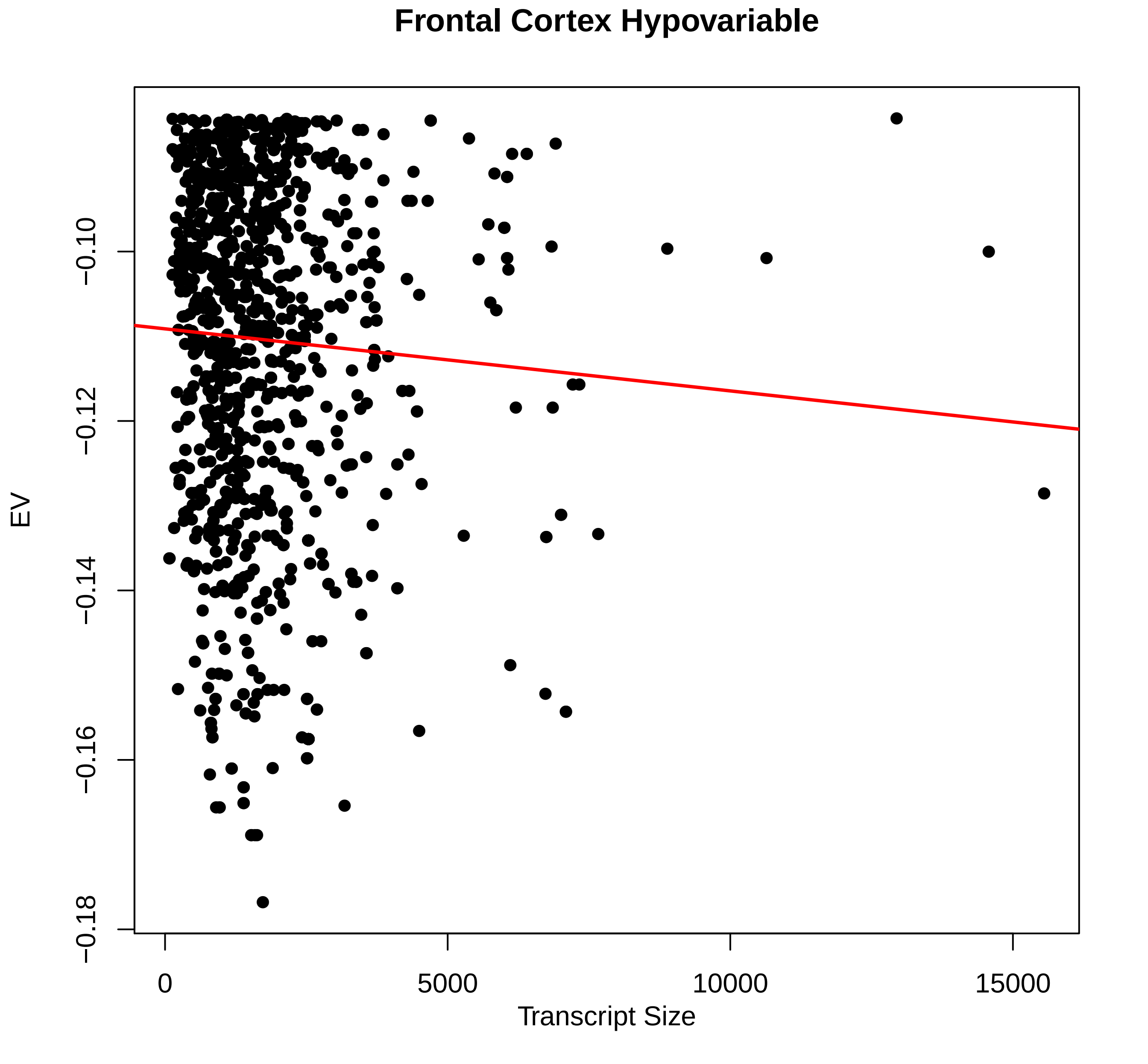


Intercept: −0.109 Slope: −7.32e−07 R2: 0.00179 Correlation: −0.0428

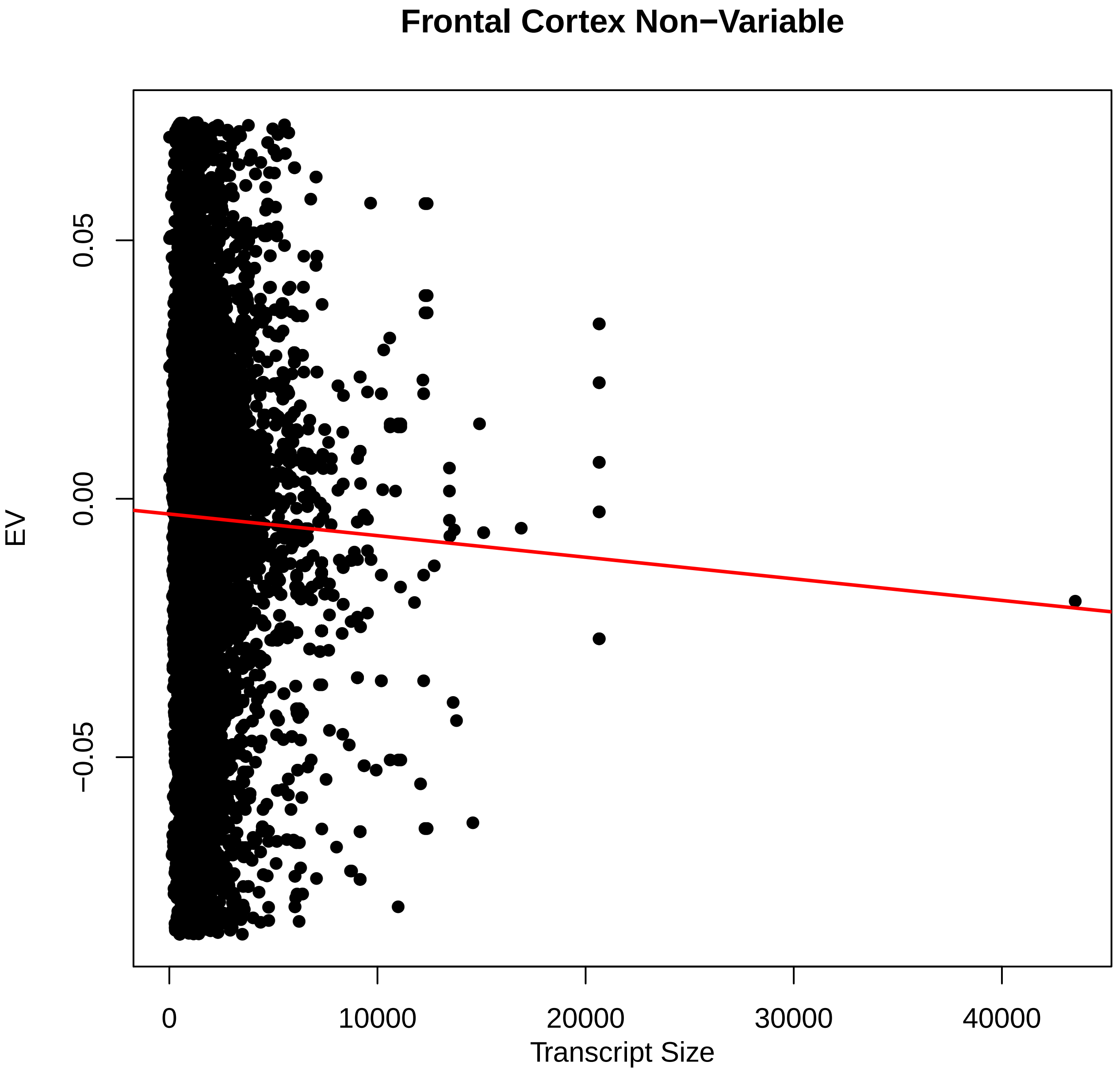


Intercept: −0.00296 Slope: −4.18e−07 R2: 0.000441 Correlation: −0.0227

# Number of Transcripts Linear Regression Analysis

| | Tissue | Class | Intercept | Slope | P.value |
|---|---|---|---|---|---|
| [1,] | Breast | Hypervariable | 0.333035870 | −5.128550e−04 | 0.160623977 |
| [2,] | Breast | Hypovariable | −0.178965643 | 2.643703e−05 | 0.543656485 |
| [3,] | Breast | Non−Variable | −0.005365237 | −5.736131e−05 | 0.007532805 |
| [4,] | Cerebellum | Hypervariable | 0.148933312 | −1.626243e−04 | 0.135821722 |
| [5,] | Cerebellum | Hypovariable | −0.105217830 | −7.589073e−06 | 0.753650866 |
| [6,] | Cerebellum | Non−Variable | −0.004661885 | 1.819036e−06 | 0.901925770 |
| [7,] | Frontal Cortex | Hypervariable | 0.167658560 | −4.744168e−05 | 0.694528762 |
| [8,] | Frontal Cortex | Hypovariable | −0.111816198 | −2.798615e−05 | 0.305860106 |
| [9,] | Frontal Cortex | Non−Variable | −0.004534336 | −5.438156e−06 | 0.729377781 |

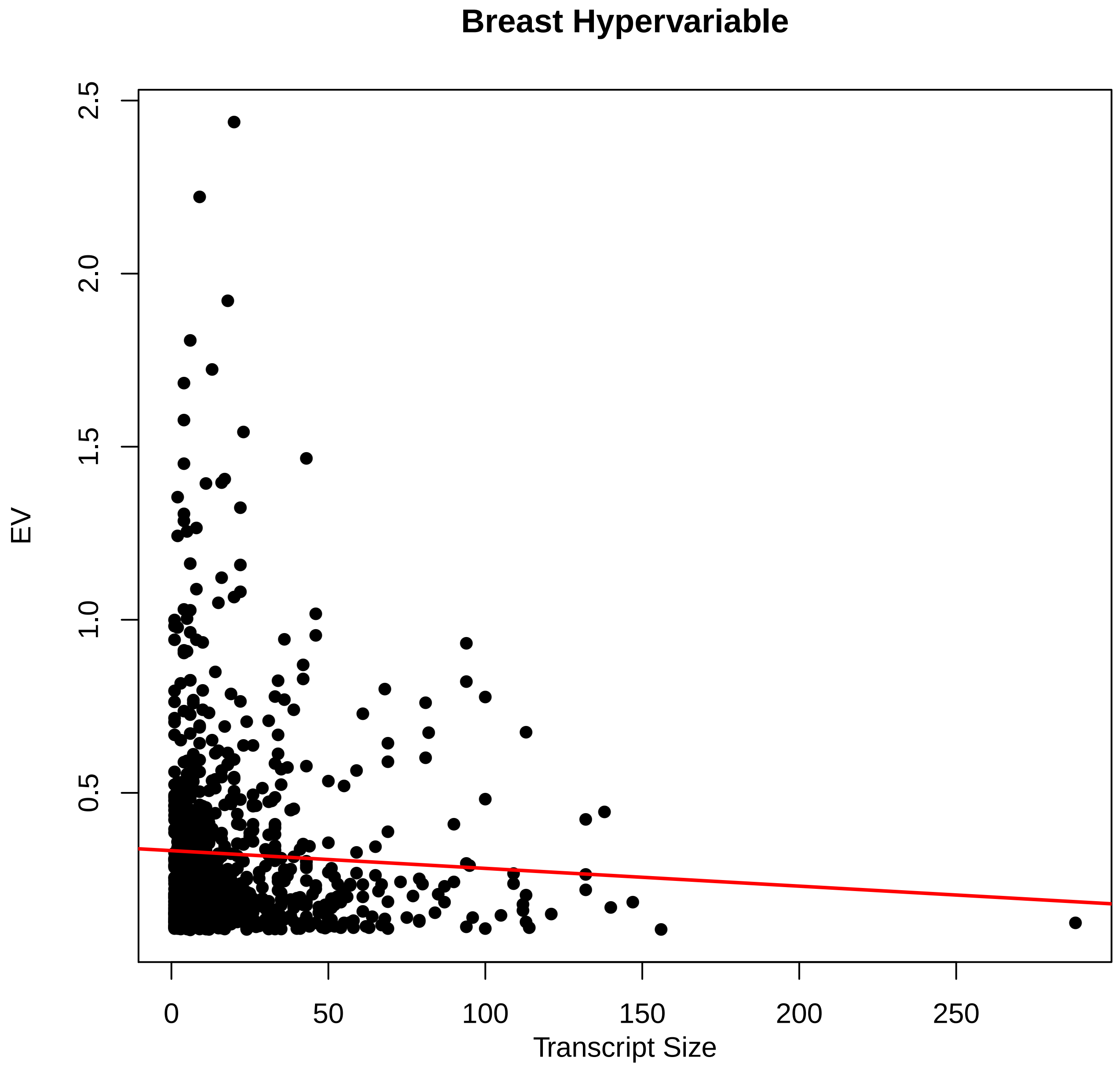


Intercept: 0.333 Slope: −0.000513 R2: 0.00111 Correlation: −0.0237

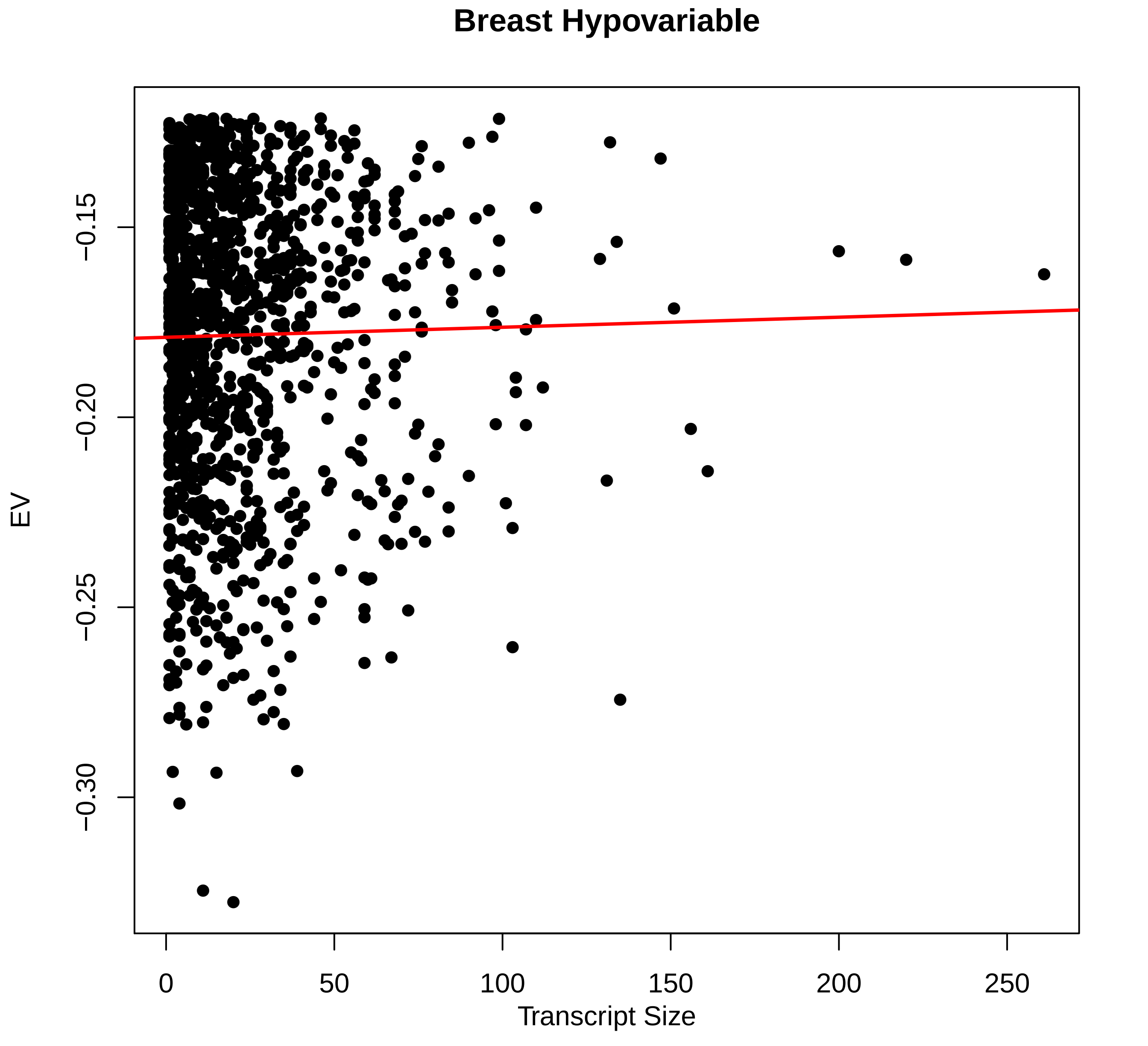


Intercept: −0.179 Slope: 2.64e−05 R2: −0.000535 Correlation: 0.0206

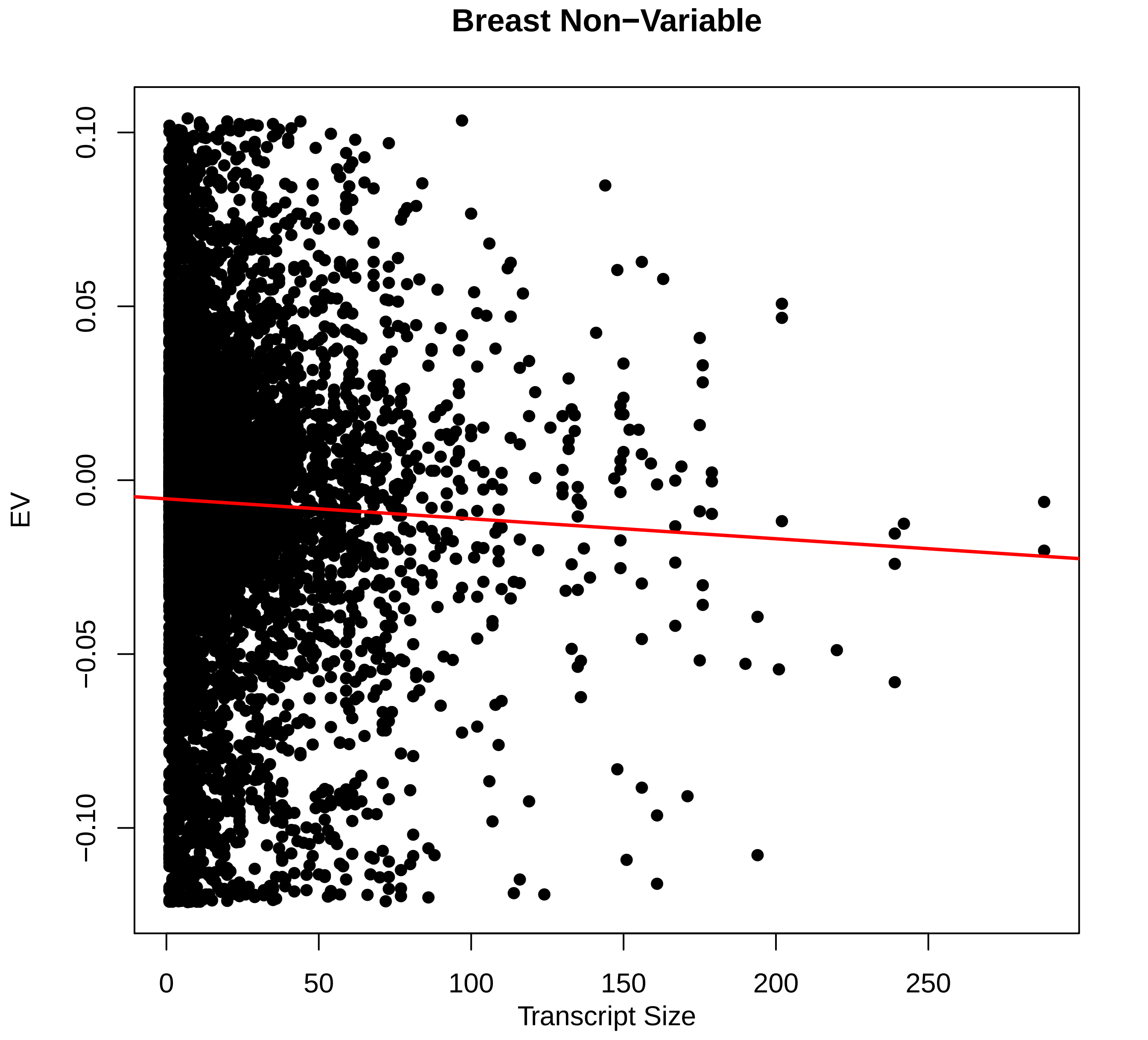


Intercept: −0.00537    Slope: −5.74e−05    R2: 0.00113    Correlation: −0.019

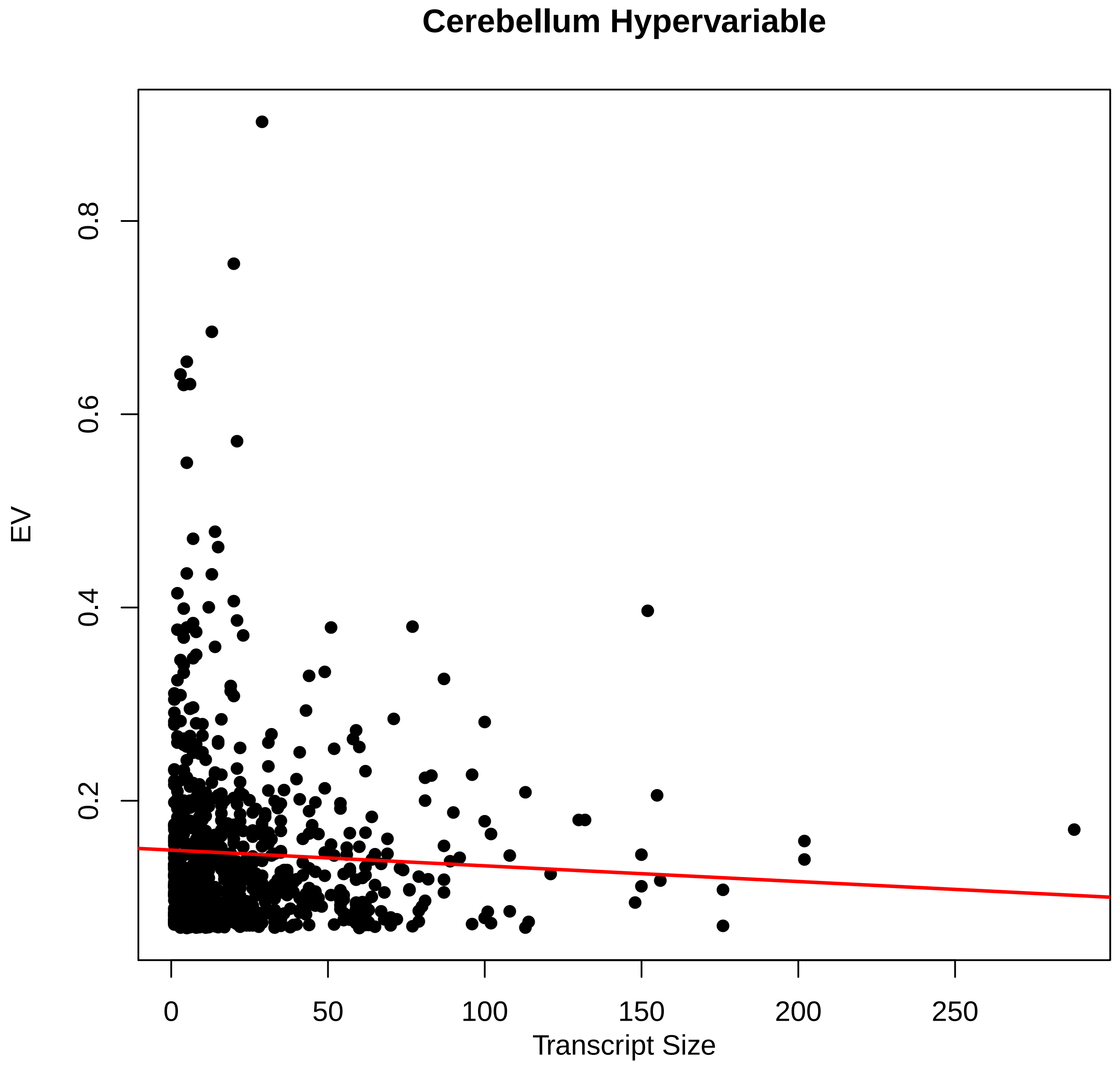


Intercept: 0.149 Slope: −0.000163 R2: 0.00156 Correlation: −0.0545

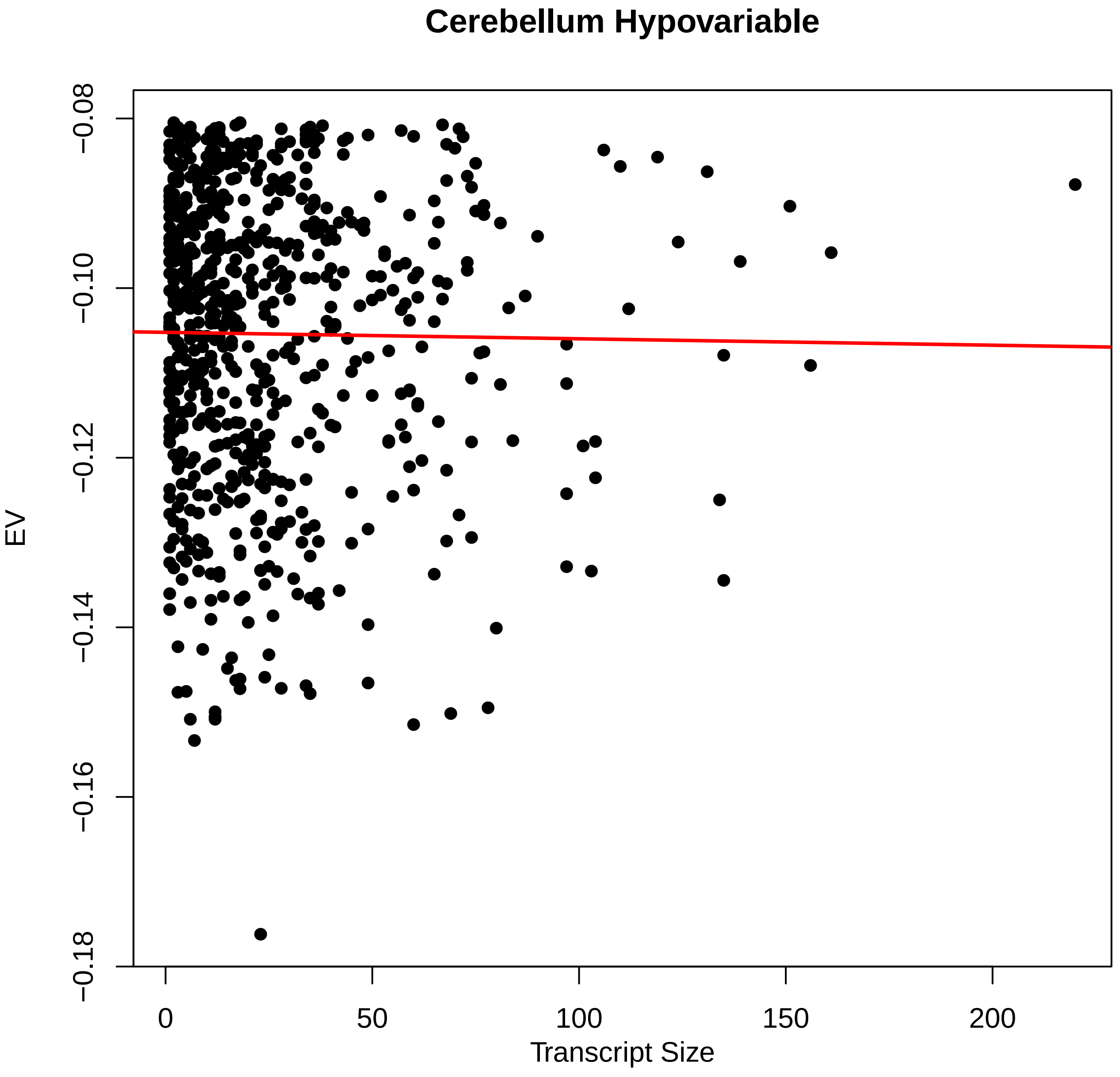


Intercept: −0.105 Slope: −7.59e−06 R2: −0.00129 Correlation: −0.0186

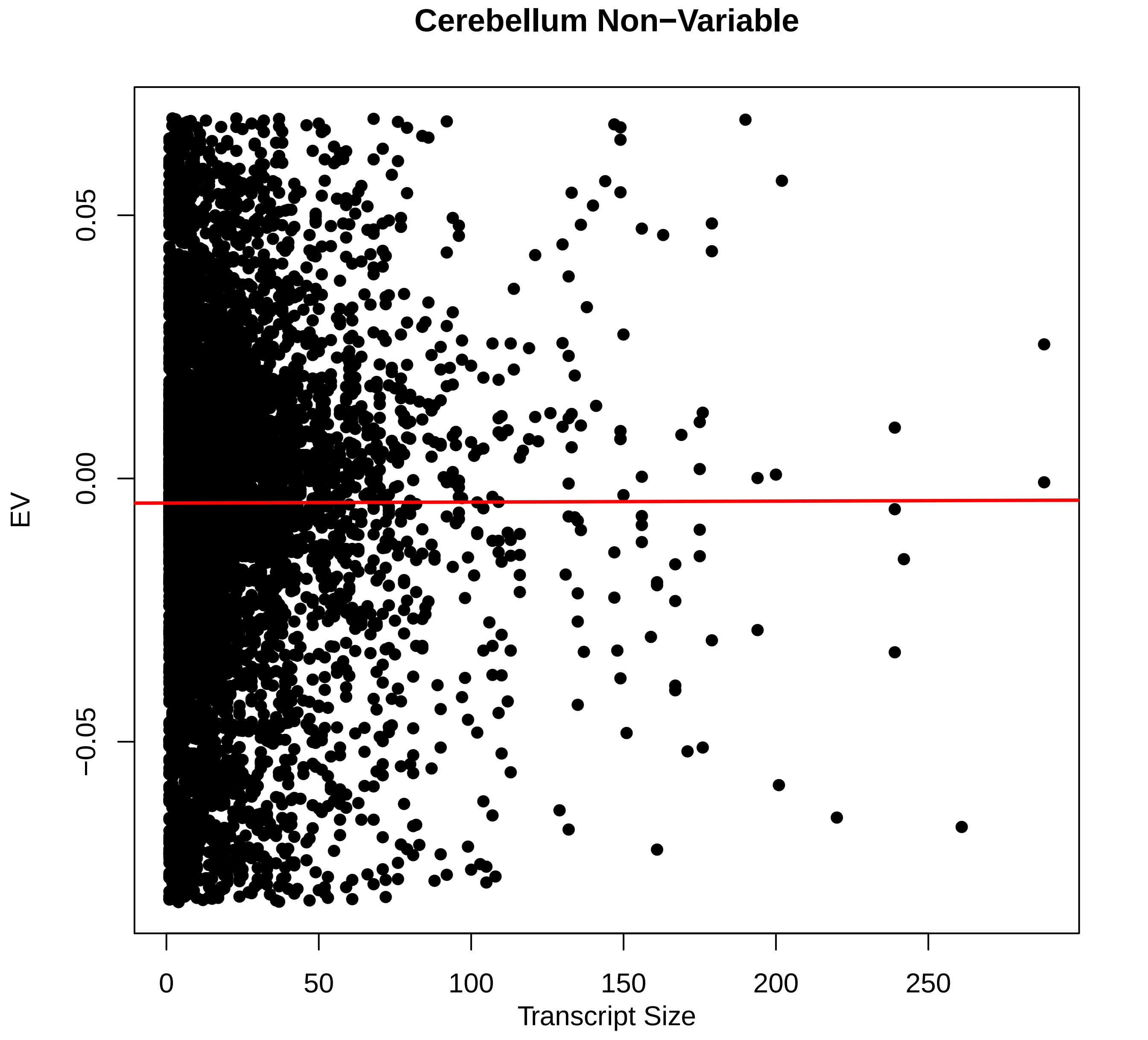


Intercept: −0.00466 Slope: 1.82e−06 R2: −0.000164 Correlation: 0.000546

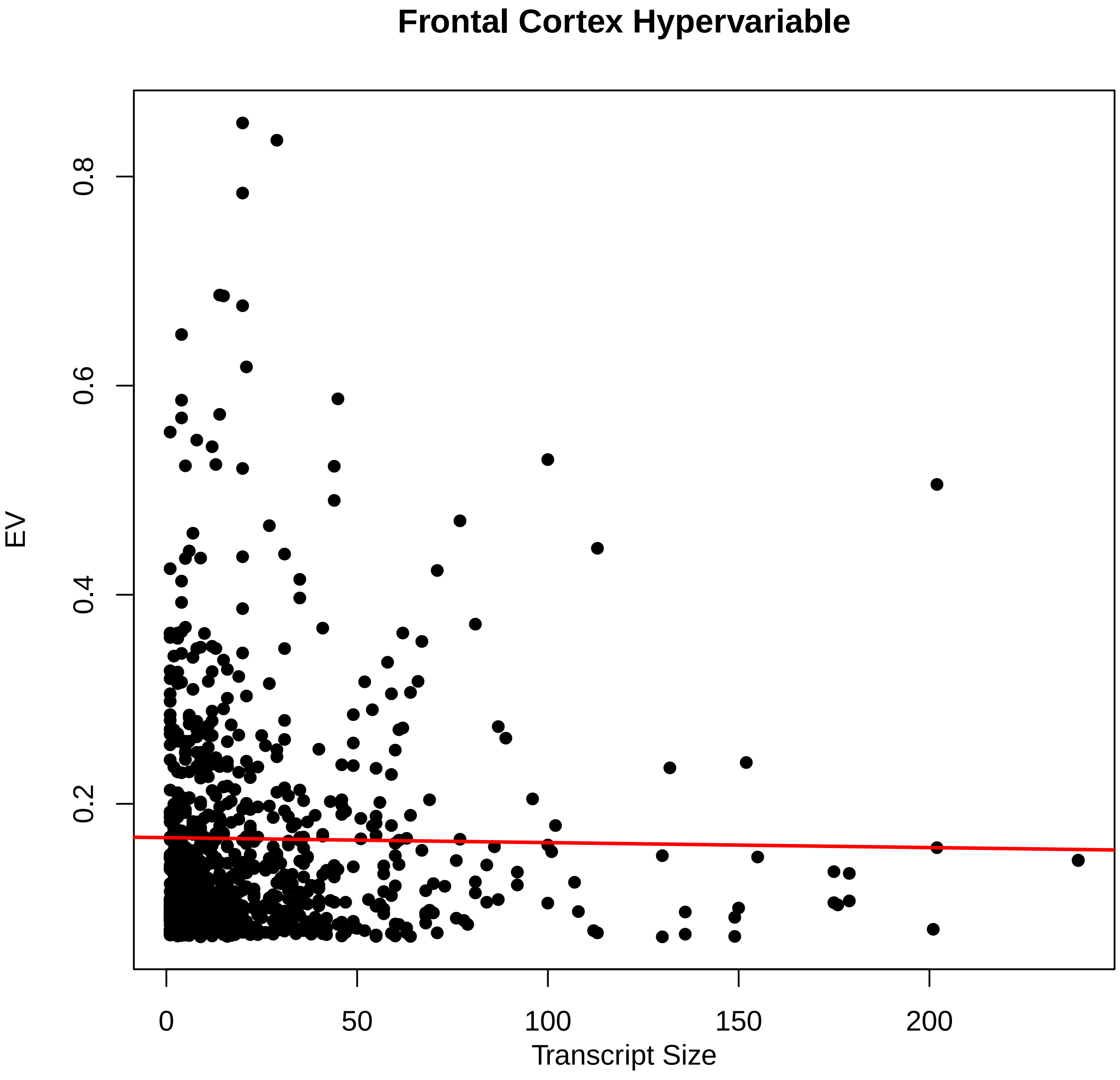


Intercept: 0.168 Slope: −4.74e−05 R2: −0.00105 Correlation: −0.0261

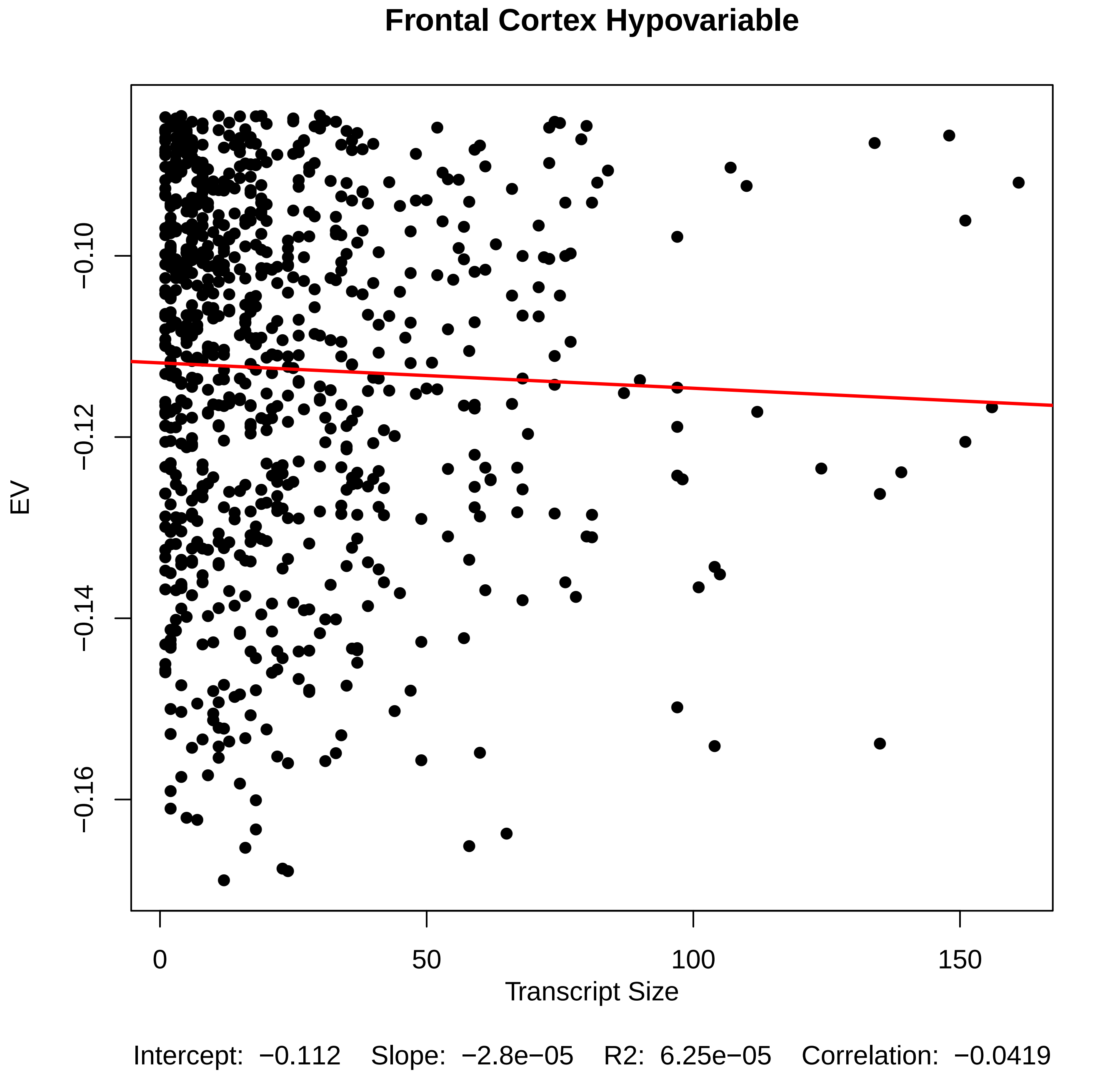
Frontal Cortex Hypovariable
EV
−0.10
−0.12
−0.14
−0.16
0
50
100
150
Transcript Size
Intercept: −0.112 Slope: −2.8e−05 R2: 6.25e−05 Correlation: −0.0419

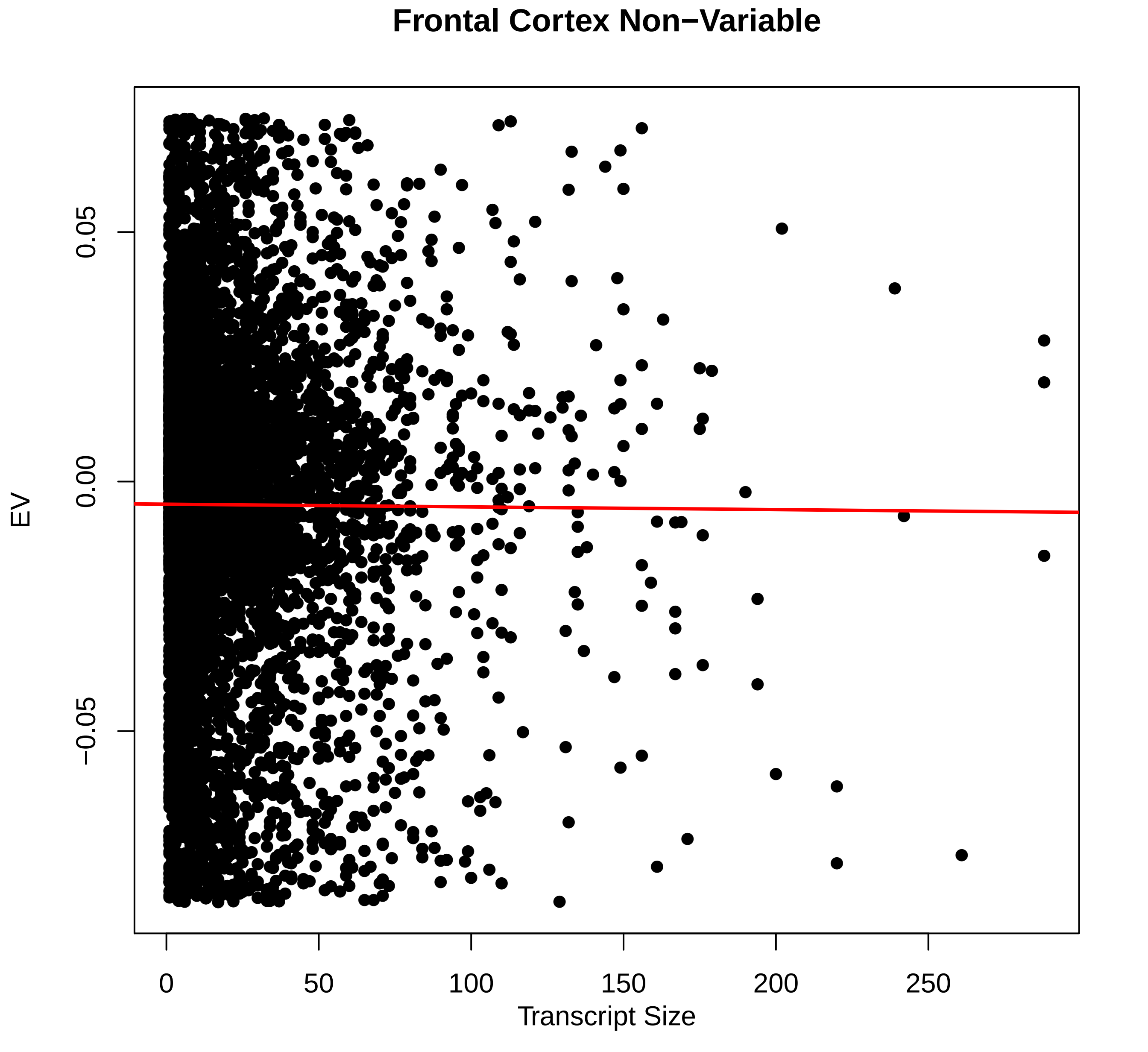


Intercept: −0.00453 Slope: −5.44e−06 R2: −0.000149 Correlation: −0.00117
