# Supplementary material for: Human gene expression variability and its dependence on methylation and aging
Source: BMC Genomics. 2019 Dec 7;20:941. doi: 10.1186/s12864-019-6308-7 (PMC6898959; doi:10.1186/s12864-019-6308-7)
Supplement: Supplementary file 2 — Additional file 2. EV Correlation between different tissue types [file 12864_2019_6308_MOESM2_ESM.pdf]

Additional File 2. EV correlation between different tissue types

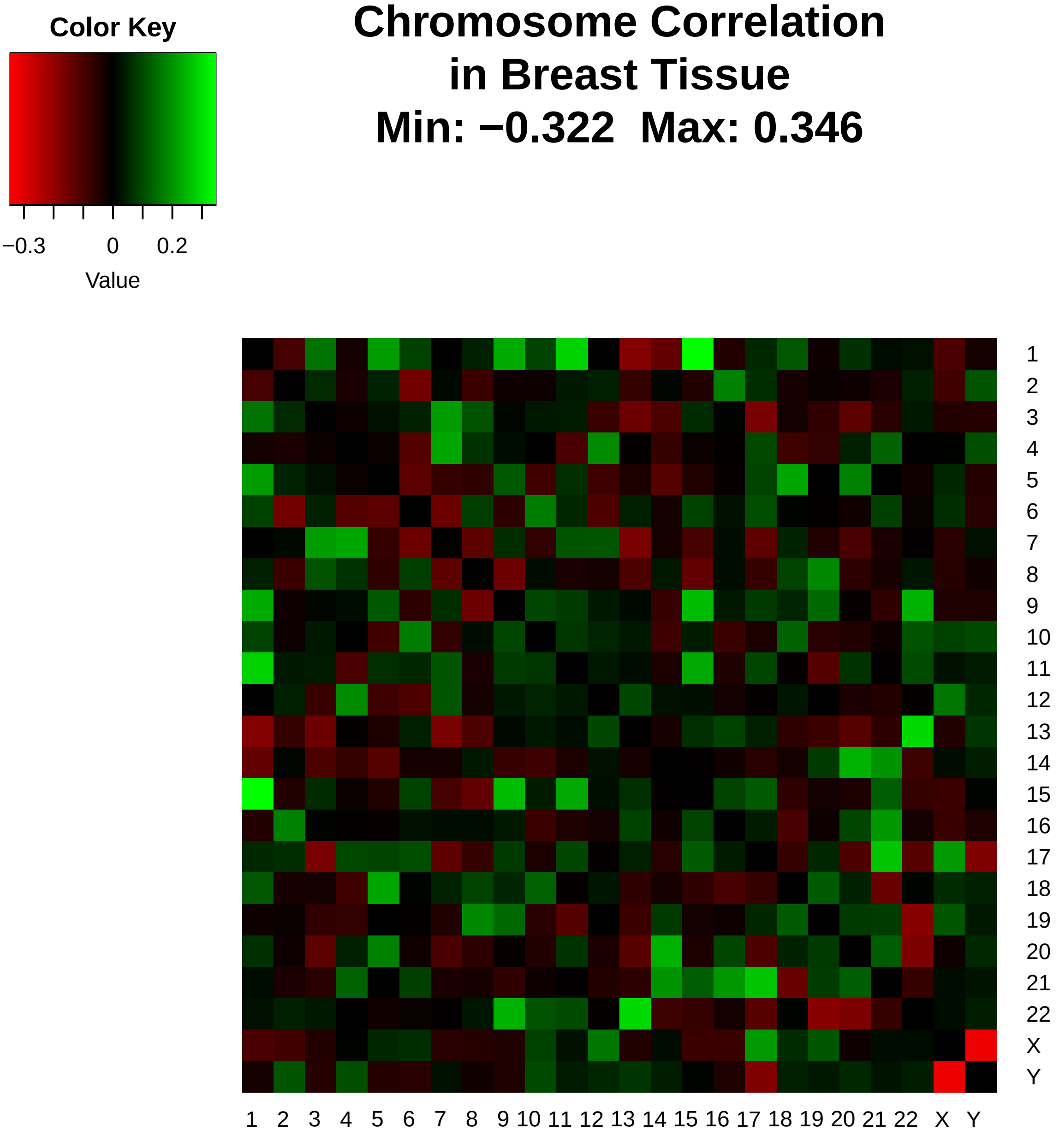

Color Key
−0.3
0
0.2
Value
Chromosome Correlation
in Breast Tissue
Min: −0.322 Max: 0.346
1
2
3
4
5
6
7
8
9
10
11
12
13
14
15
16
17
18
19
20
21
22
X
Y

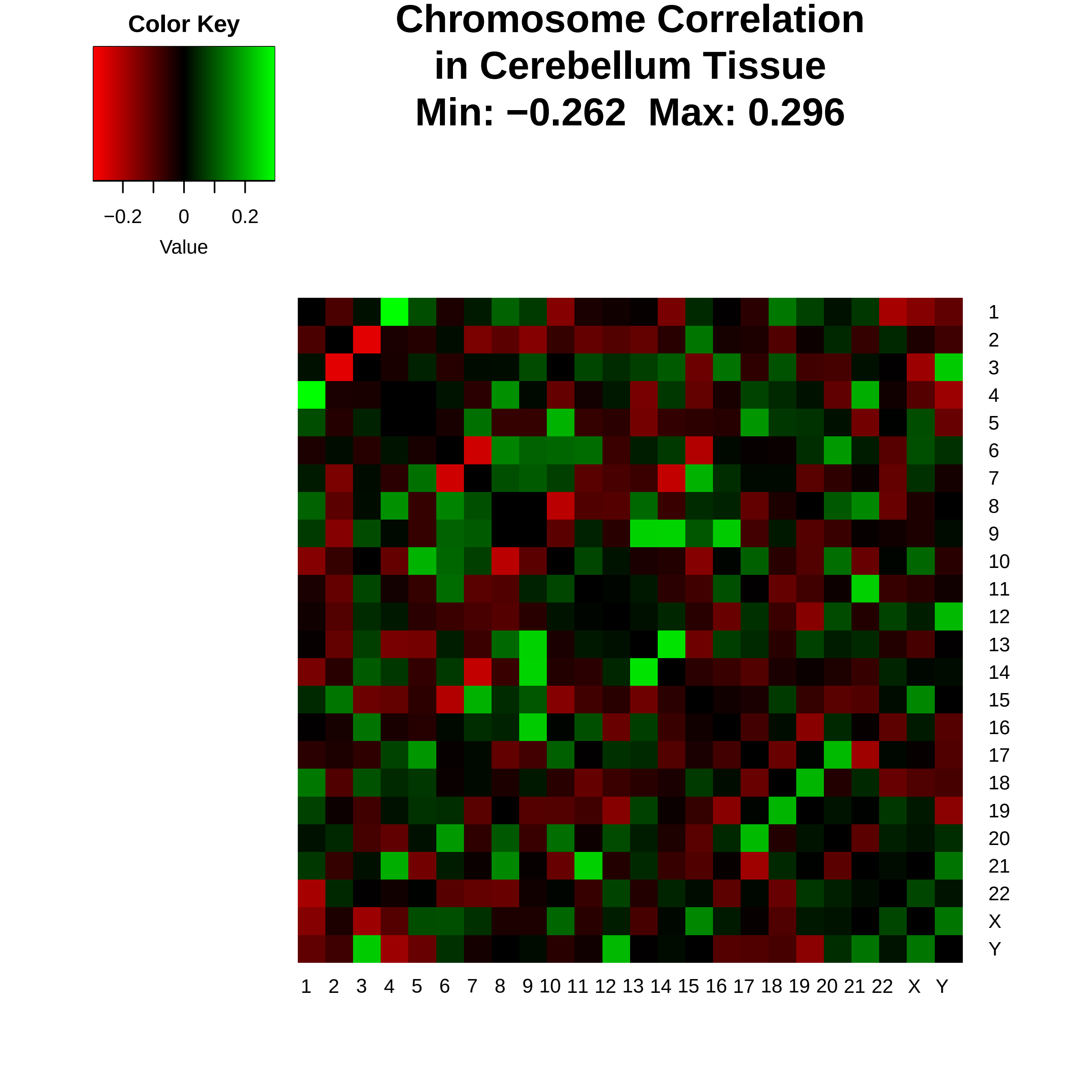
Chromosome Correlation
in Cerebellum Tissue
Min: −0.262 Max: 0.296
Color Key
−0.2
0
0.2
Value
1
2
3
4
5
6
7
8
9
10
11
12
13
14
15
16
17
18
19
20
21
22
X
Y

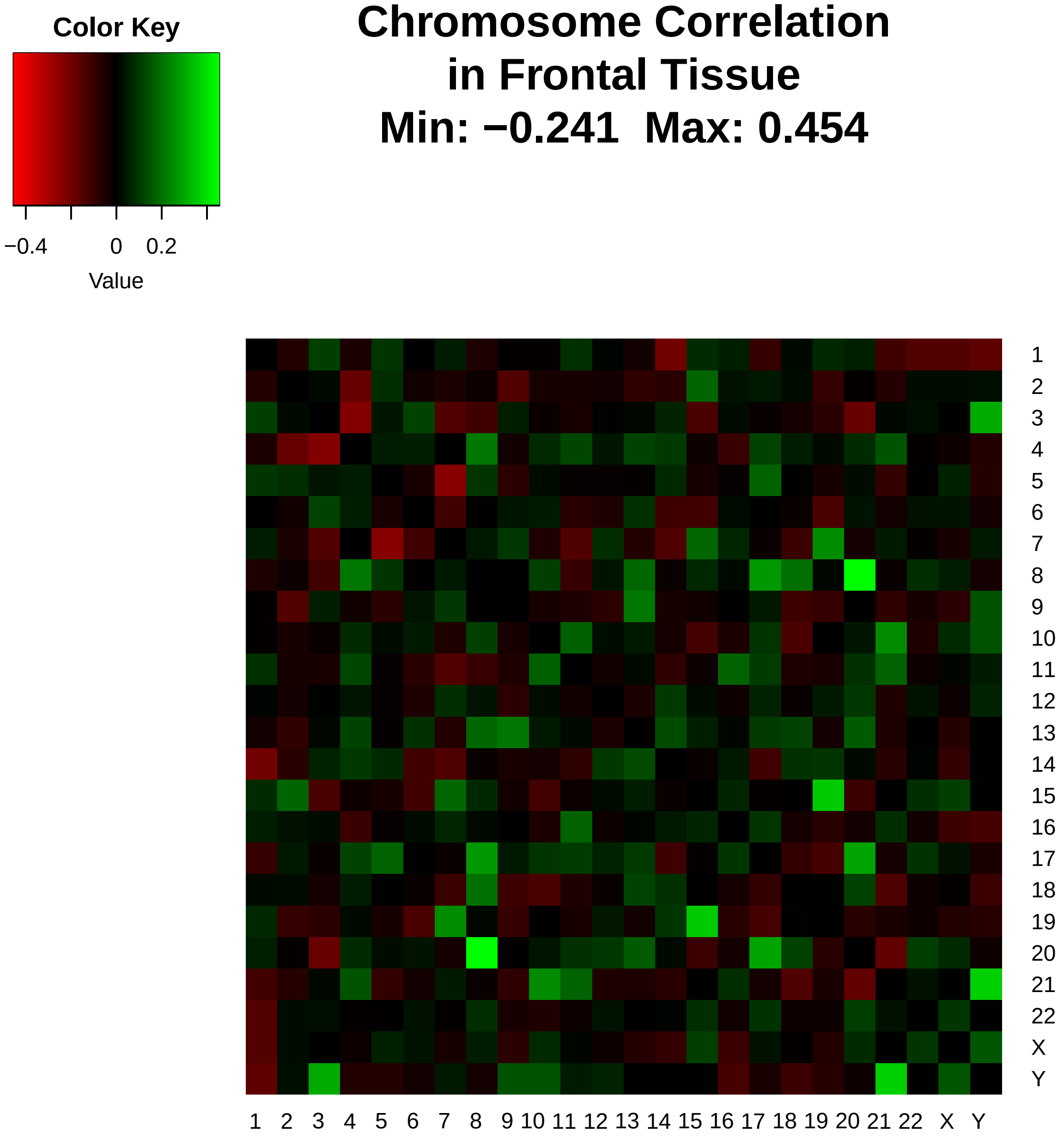
Color Key
−0.4
0
0.2
Value
Chromosome Correlation
in Frontal Tissue
Min: −0.241 Max: 0.454
1 2 3 4 5 6 7 8 9 10 11 12 13 14 15 16 17 18 19 20 21 22 X Y
1 2 3 4 5 6 7 8 9 10 11 12 13 14 15 16 17 18 19 20 21 22 X Y

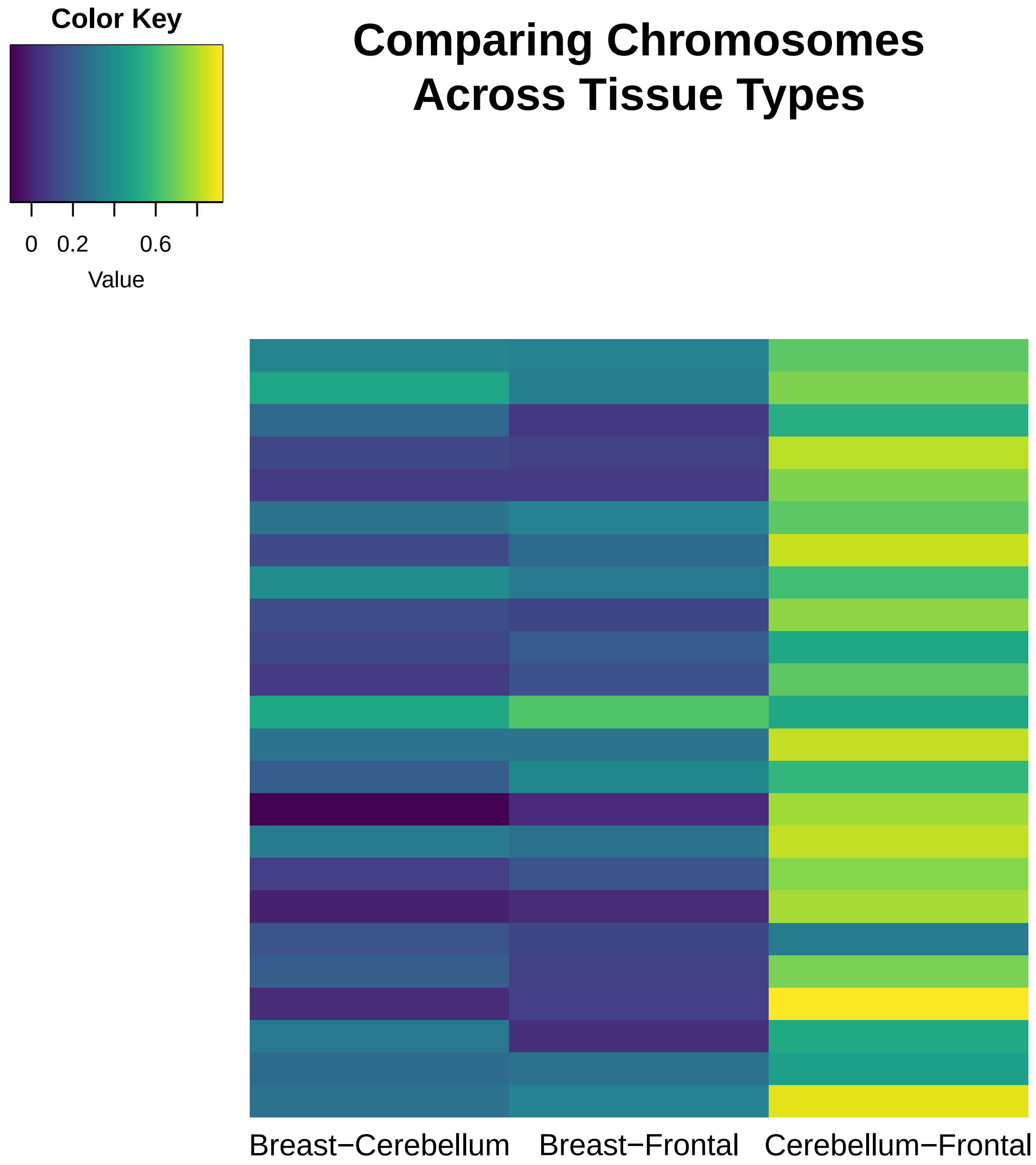
Color Key
0
0.2
0.6
Value
Comparing Chromosomes
Across Tissue Types
1
2
3
4
5
6
7
8
9
10
11
12
13
14
15
16
17
18
19
20
21
22
X
Y
Breast−Cerebellum
Breast−Frontal
Cerebellum−Frontal
