# Supplementary material for: Human gene expression variability and its dependence on methylation and aging
Source: BMC Genomics. 2019 Dec 7;20:941. doi: 10.1186/s12864-019-6308-7 (PMC6898959; doi:10.1186/s12864-019-6308-7)

# Additional File 3. Complete list of GO terms for all genes

Common Hypervariable Biological Processes

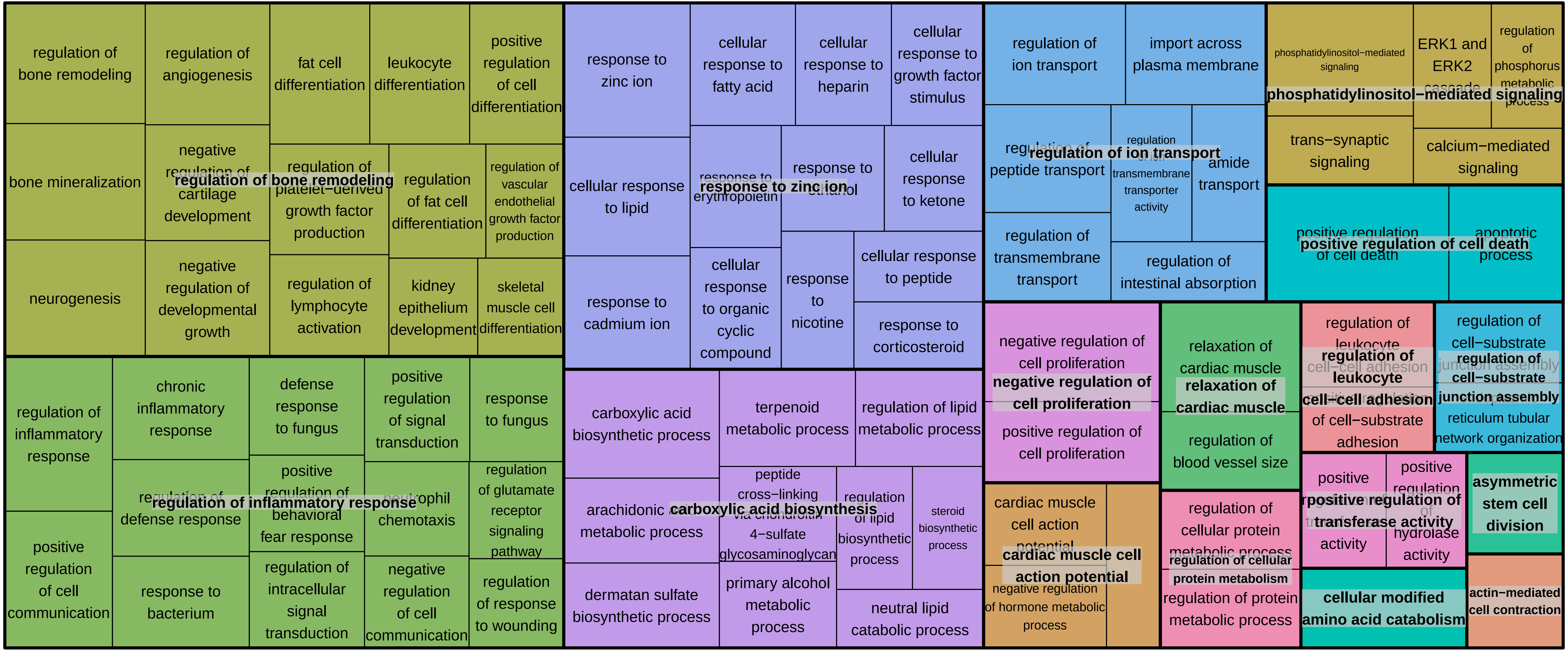

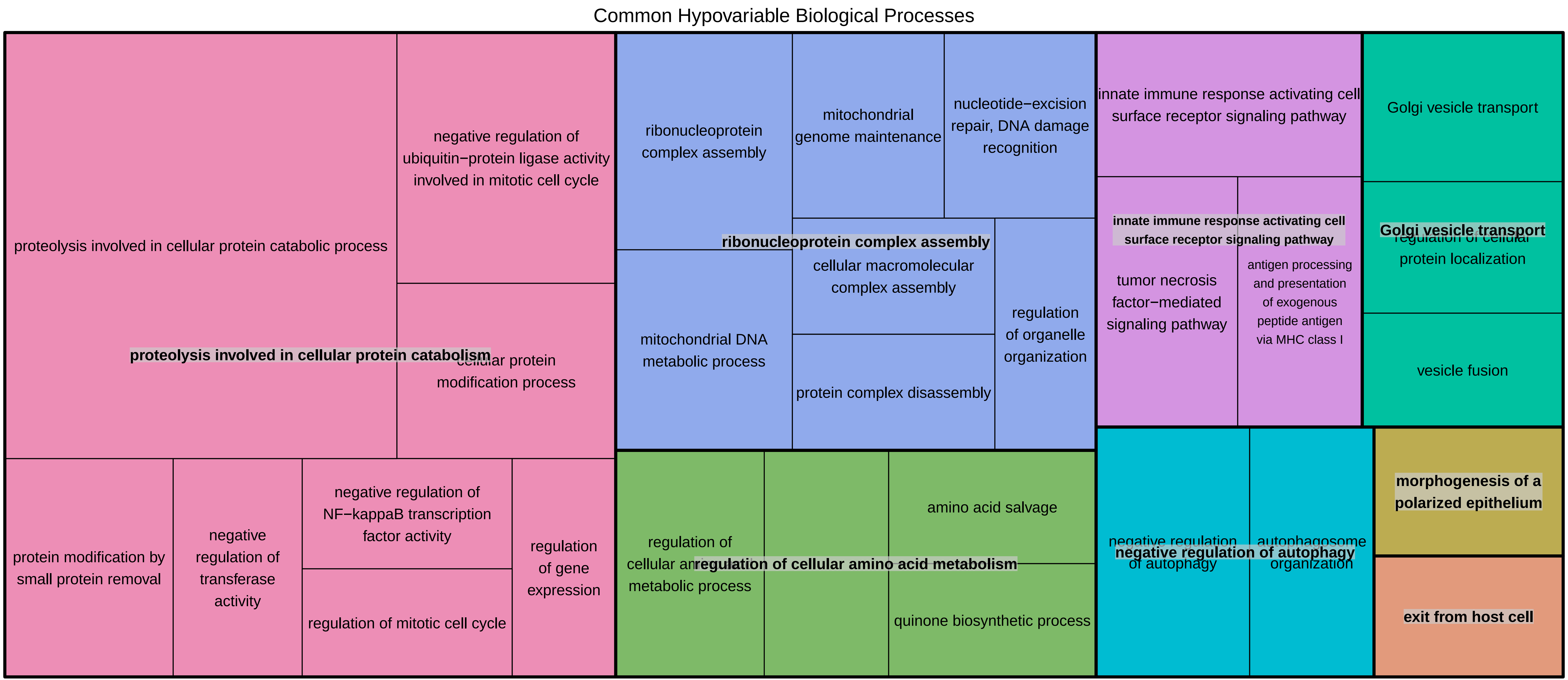

Common Hypovariable Biological Processes
proteolysis involved in cellular protein catabolic process
negative regulation of ubiquitin−protein ligase activity involved in mitotic cell cycle
proteolysis involved in cellular protein catabolism
modification process
protein modification by small protein removal
negative regulation of transferase activity
negative regulation of NF−kappaB transcription factor activity
regulation of mitotic cell cycle
regulation of gene expression
ribonucleoprotein complex assembly
mitochondrial genome maintenance
nucleotide−excision repair, DNA damage recognition
ribonucleoprotein complex assembly
cellular macromolecular complex assembly
mitochondrial DNA metabolic process
regulation of organelle organization
protein complex disassembly
regulation of
metabolic process
amino acid salvage
regulation of cellular amino acid metabolism
quinone biosynthetic process
innate immune response activating cell surface receptor signaling pathway
innate immune response activating cell surface receptor signaling pathway
tumor necrosis factor−mediated signaling pathway
antigen processing and presentation of exogenous peptide antigen via MHC class I
Golgi vesicle transport
Golgi vesicle transport
protein localization
vesicle fusion
negative regulation of autophagy
morphogenesis of a polarized epithelium
exit from host cell

Unique Hypervariable Breast Biological Processes

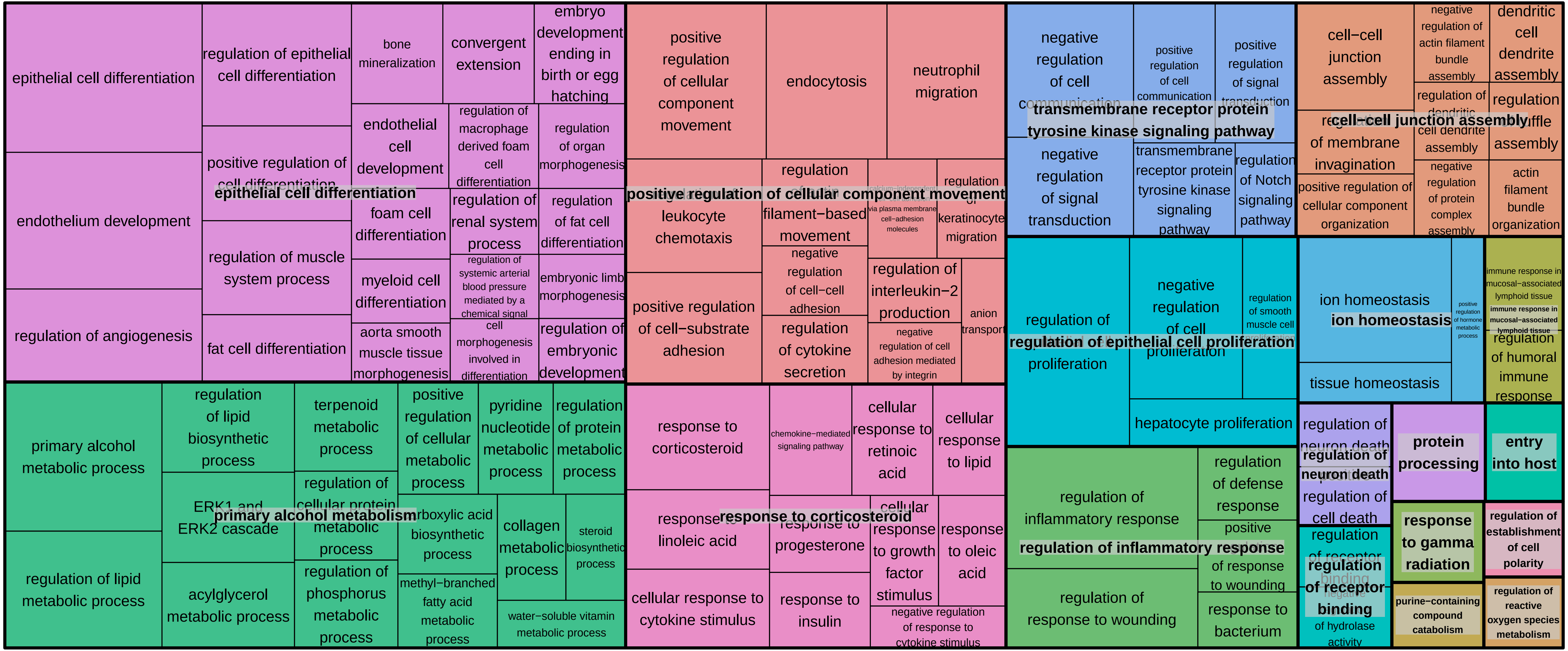

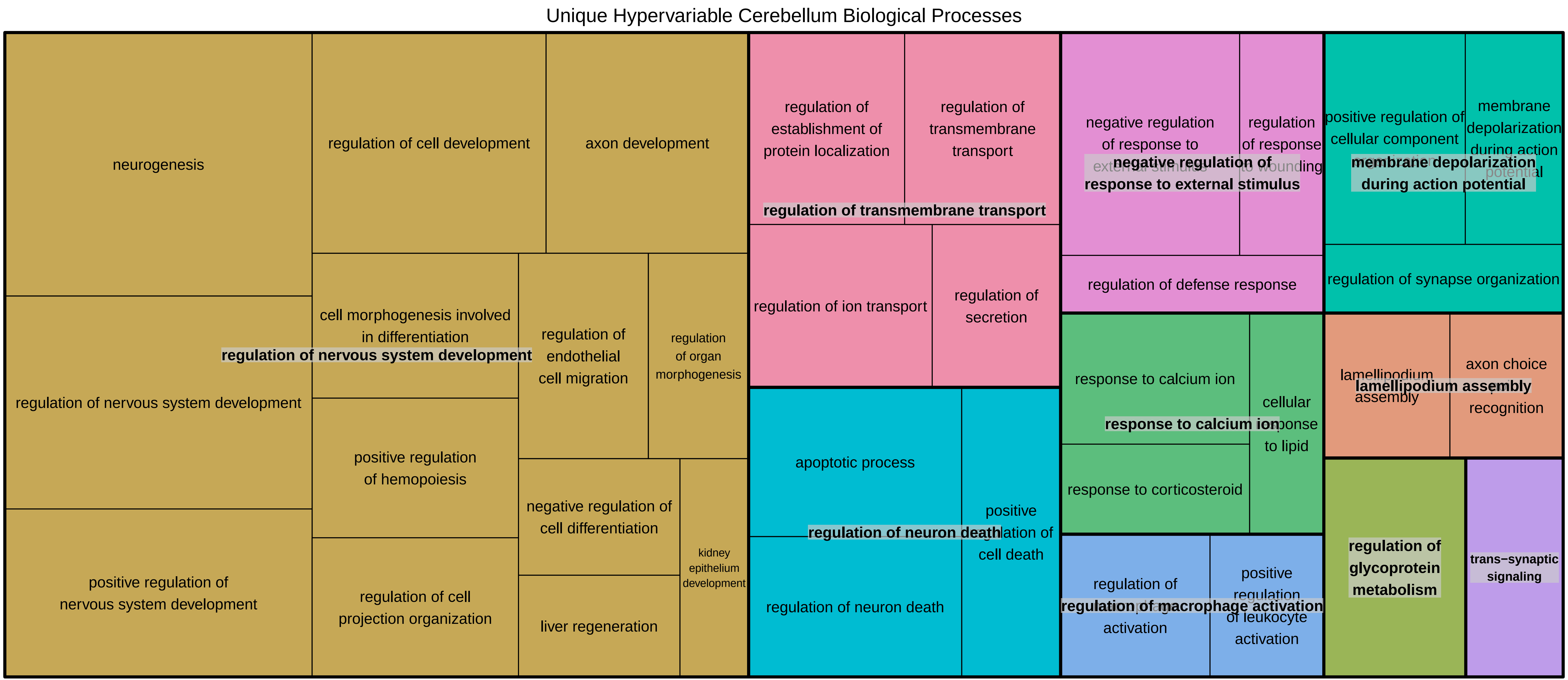
Unique Hypervariable Cerebellum Biological Processes
neurogenesis
regulation of cell development
axon development
regulation of nervous system development
cell morphogenesis involved in differentiation
regulation of endothelial cell migration
regulation of organ morphogenesis
regulation of nervous system development
positive regulation of hemopoiesis
negative regulation of cell differentiation
kidney epithelium development
positive regulation of nervous system development
regulation of cell projection organization
liver regeneration
regulation of establishment of protein localization
regulation of transmembrane transport
regulation of transmembrane transport
regulation of ion transport
regulation of secretion
apoptotic process
positive regulation of cell death
regulation of neuron death
regulation of neuron death
negative regulation of response to external stimulus
regulation of response to wounding
negative regulation of response to external stimulus
regulation of defense response
response to calcium ion
cellular response to lipid
response to calcium ion
response to corticosteroid
regulation of macrophage activation
positive regulation of leukocyte activation
regulation of macrophage activation
positive regulation of cellular component organization
membrane depolarization during action potential
membrane depolarization during action potential
regulation of synapse organization
lamellipodium assembly
axon choice recognition
lamellipodium assembly
regulation of glycoprotein metabolism
trans−synaptic signaling

Unique Hypervariable Frontal Biological Processes

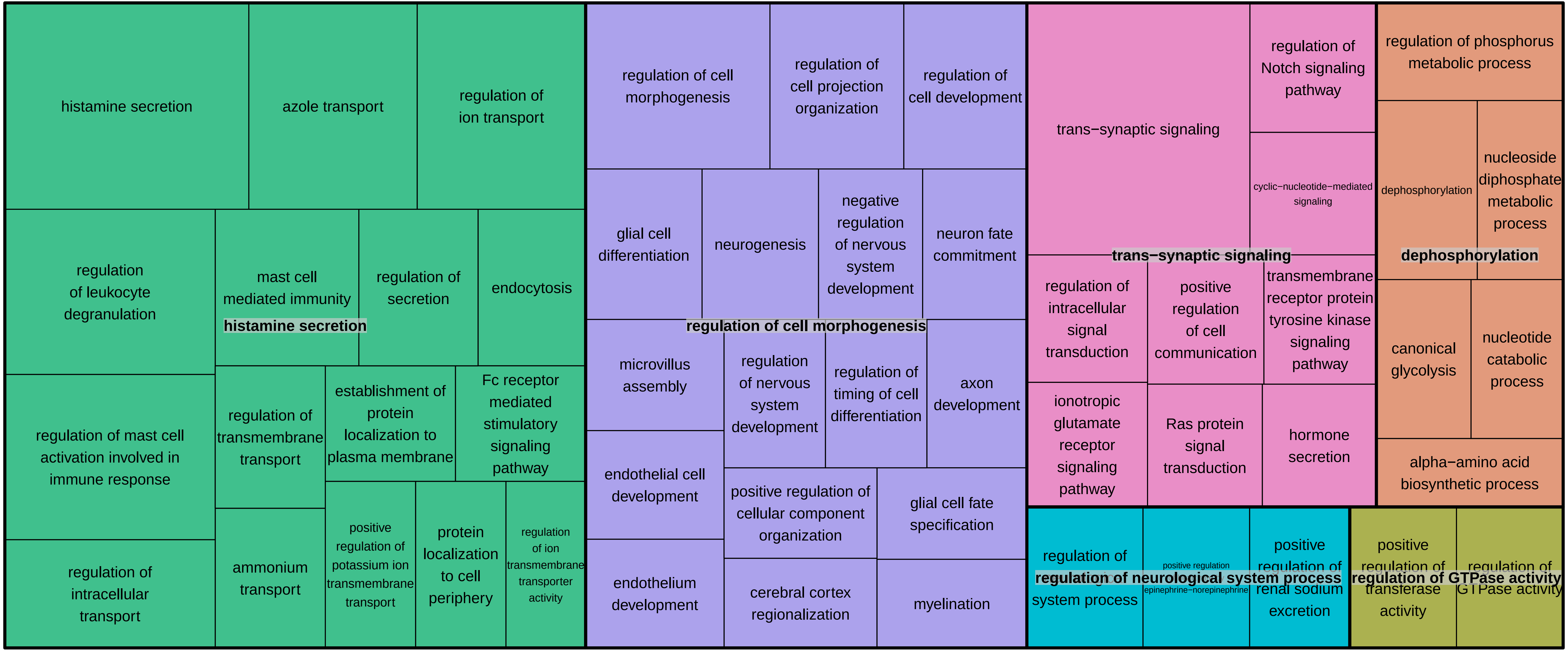

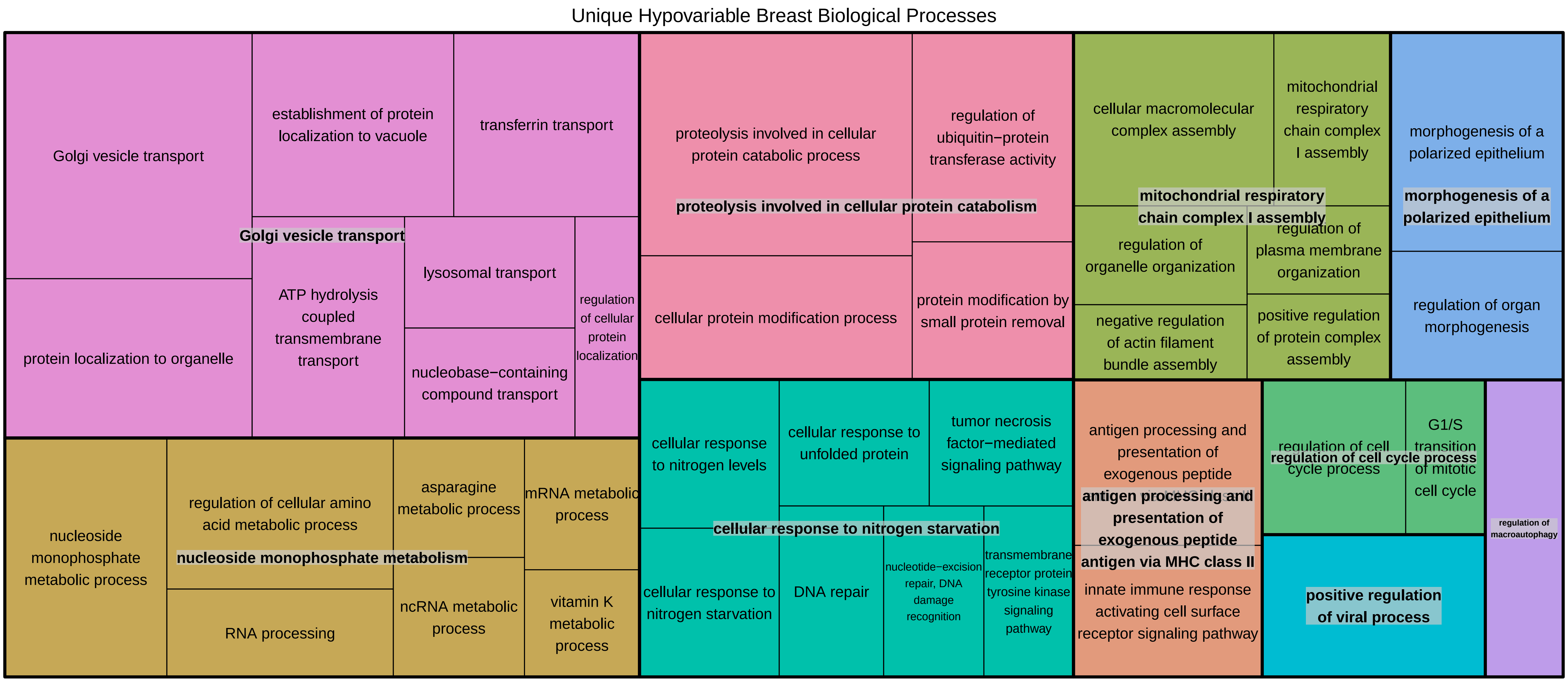

Unique Hypovariable Breast Biological Processes
Golgi vesicle transport
establishment of protein localization to vacuole
transferrin transport
protein localization to organelle
Golgi vesicle transport
ATP hydrolysis coupled transmembrane transport
lysosomal transport
nucleobase−containing compound transport
regulation of cellular protein localization
proteolysis involved in cellular protein catabolic process
regulation of ubiquitin−protein transferase activity
proteolysis involved in cellular protein catabolism
cellular protein modification process
protein modification by small protein removal
cellular macromolecular complex assembly
mitochondrial respiratory chain complex I assembly
mitochondrial respiratory chain complex I assembly
regulation of organelle organization
regulation of plasma membrane organization
negative regulation of actin filament bundle assembly
positive regulation of protein complex assembly
morphogenesis of a polarized epithelium
morphogenesis of a polarized epithelium
regulation of organ morphogenesis
nucleoside monophosphate metabolic process
regulation of cellular amino acid metabolic process
nucleoside monophosphate metabolism
asparagine metabolic process
mRNA metabolic process
RNA processing
ncRNA metabolic process
vitamin K metabolic process
cellular response to nitrogen levels
cellular response to unfolded protein
tumor necrosis factor−mediated signaling pathway
cellular response to nitrogen starvation
cellular response to nitrogen starvation
DNA repair
nucleotide−excision repair, DNA damage recognition
transmembrane receptor protein tyrosine kinase signaling pathway
antigen processing and presentation of exogenous peptide
antigen processing and presentation of exogenous peptide antigen via MHC class II
innate immune response activating cell surface receptor signaling pathway
regulation of cell cycle process
regulation of cell cycle process
G1/S transition of mitotic cell cycle
positive regulation of viral process
regulation of macroautophagy

Unique Hypovariable Cerebellum Biological Processes

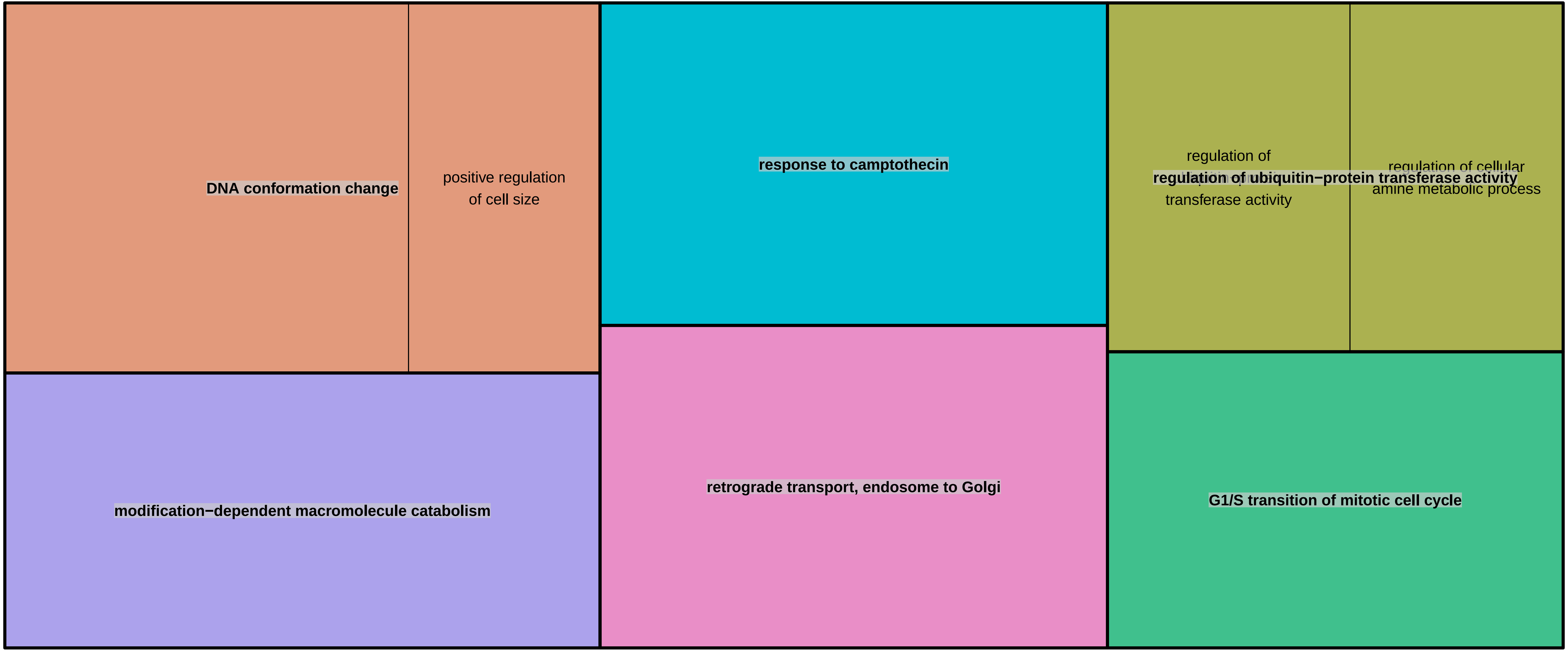

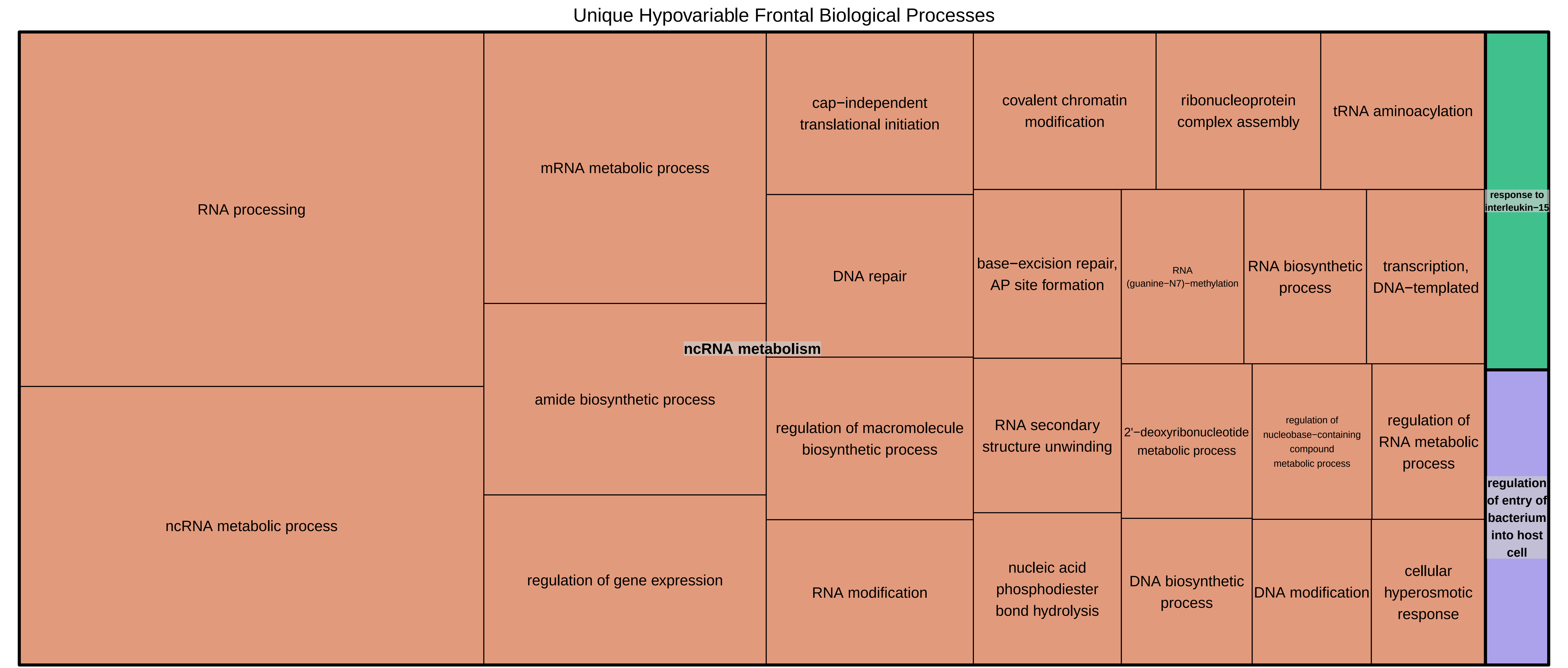
Unique Hypovariable Frontal Biological Processes
RNA processing
ncRNA metabolic process
mRNA metabolic process
amide biosynthetic process
regulation of gene expression
ncRNA metabolism
cap−independent translational initiation
DNA repair
regulation of macromolecule biosynthetic process
RNA modification
covalent chromatin modification
ribonucleoprotein complex assembly
tRNA aminoacylation
base−excision repair, AP site formation
RNA (guanine−N7)−methylation
RNA biosynthetic process
transcription, DNA−templated
RNA secondary structure unwinding
nucleic acid phosphodiester bond hydrolysis
2'−deoxyribonucleotide metabolic process
regulation of nucleobase−containing compound metabolic process
regulation of RNA metabolic process
DNA biosynthetic process
DNA modification
cellular hyperosmotic response
response to interleukin−15
regulation of entry of bacterium into host cell

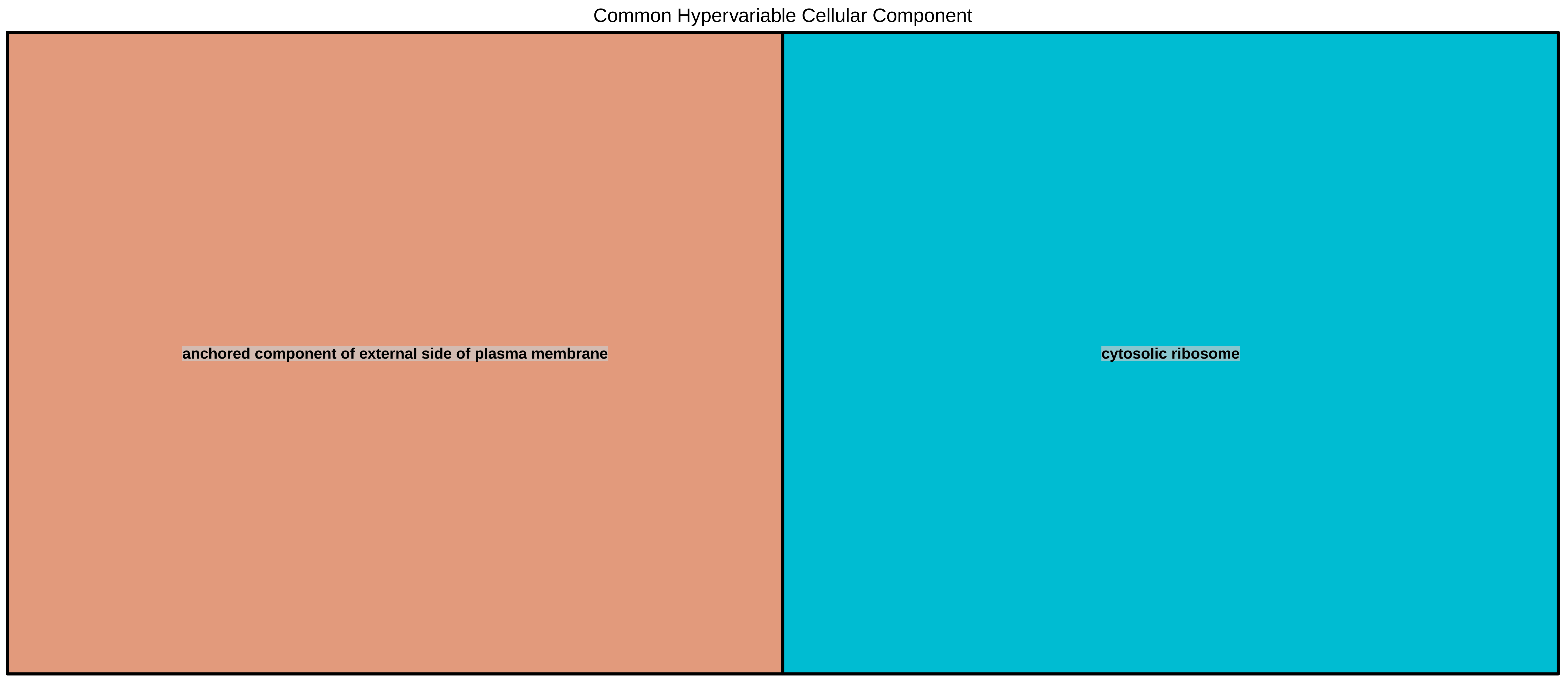
Common Hypervariable Cellular Component
anchored component of external side of plasma membrane
cytosolic ribosome

Common Hypovariable Cellular Component

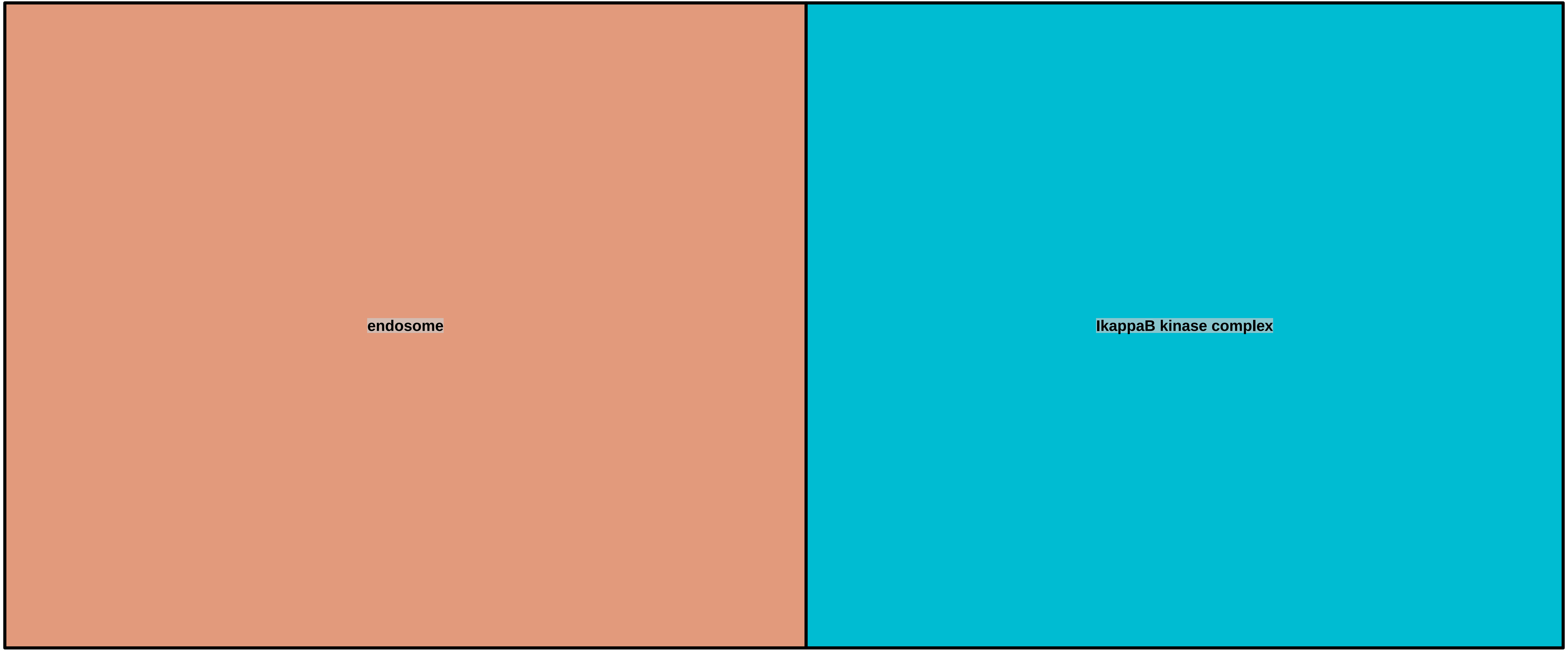

Unique Hypervariable Breast Cellular Component

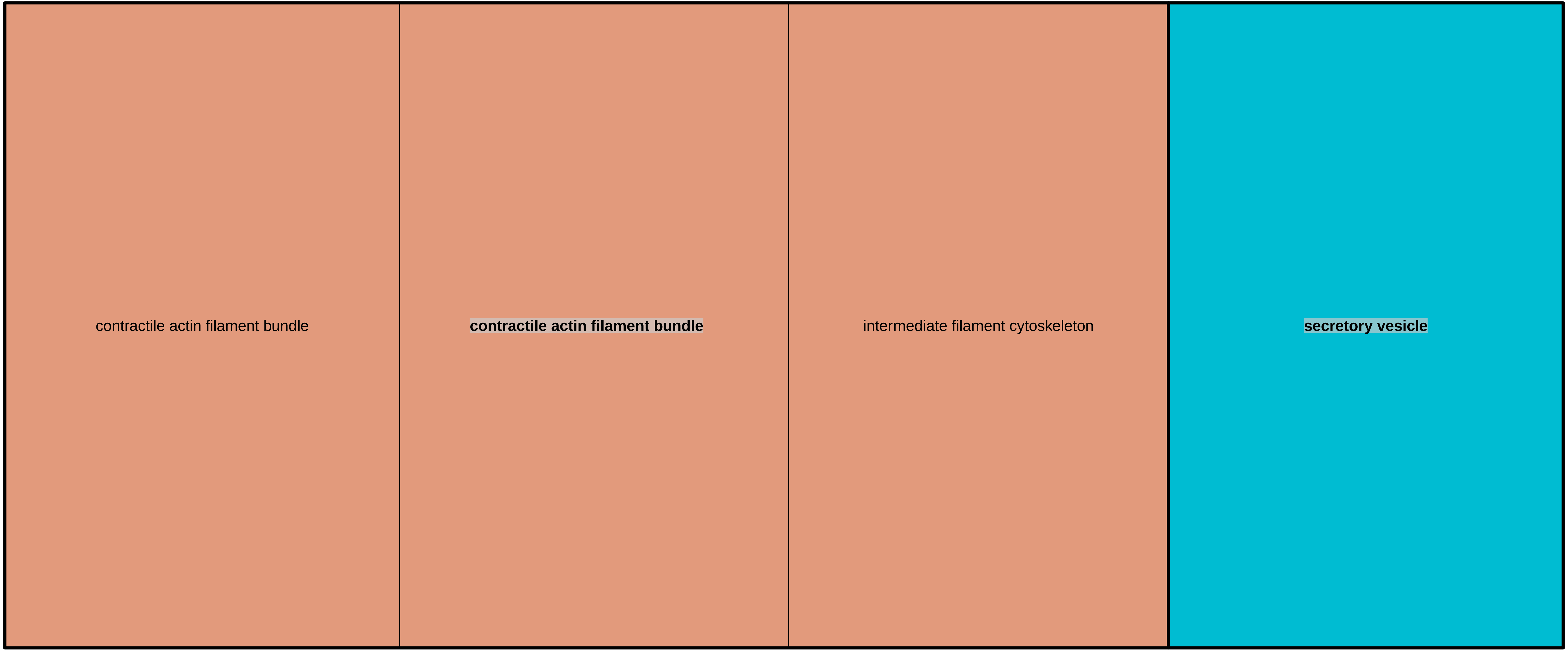

Unique Hypervariable Cerebellum Cellular Component

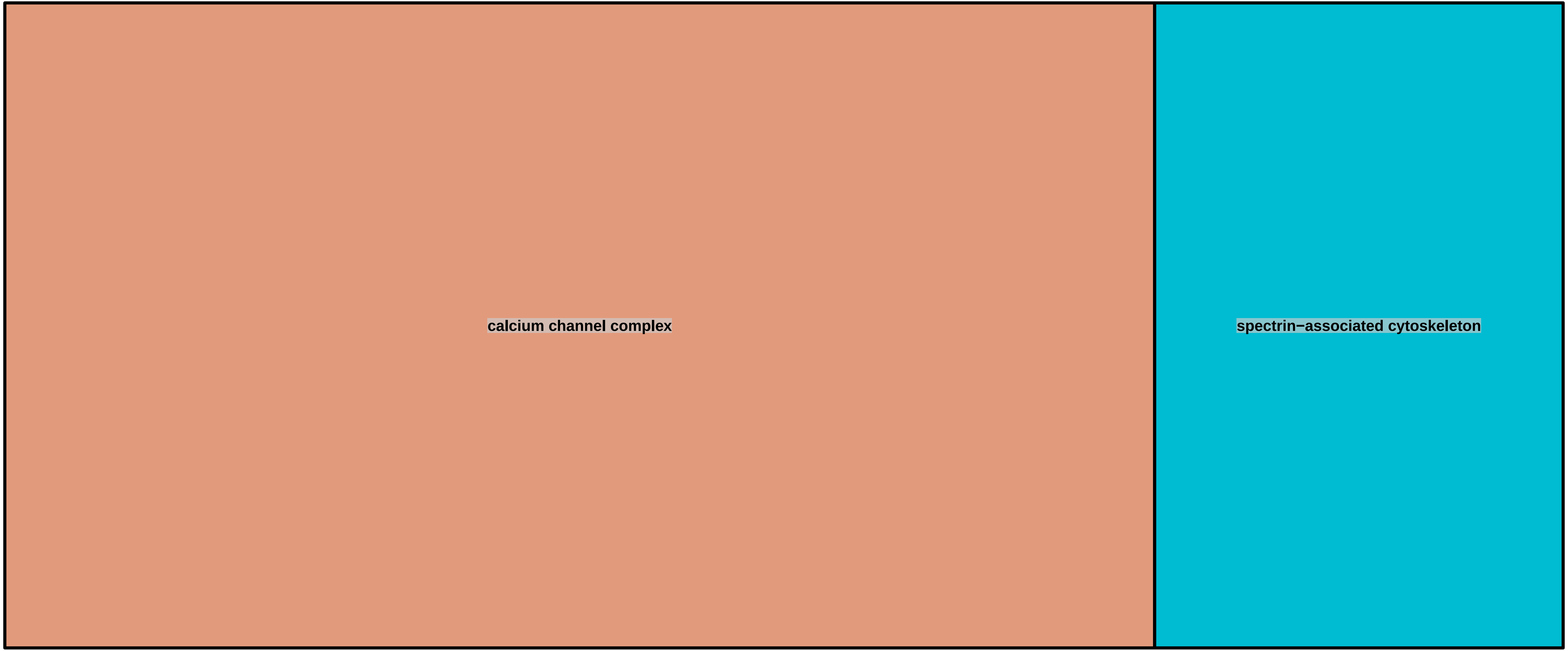

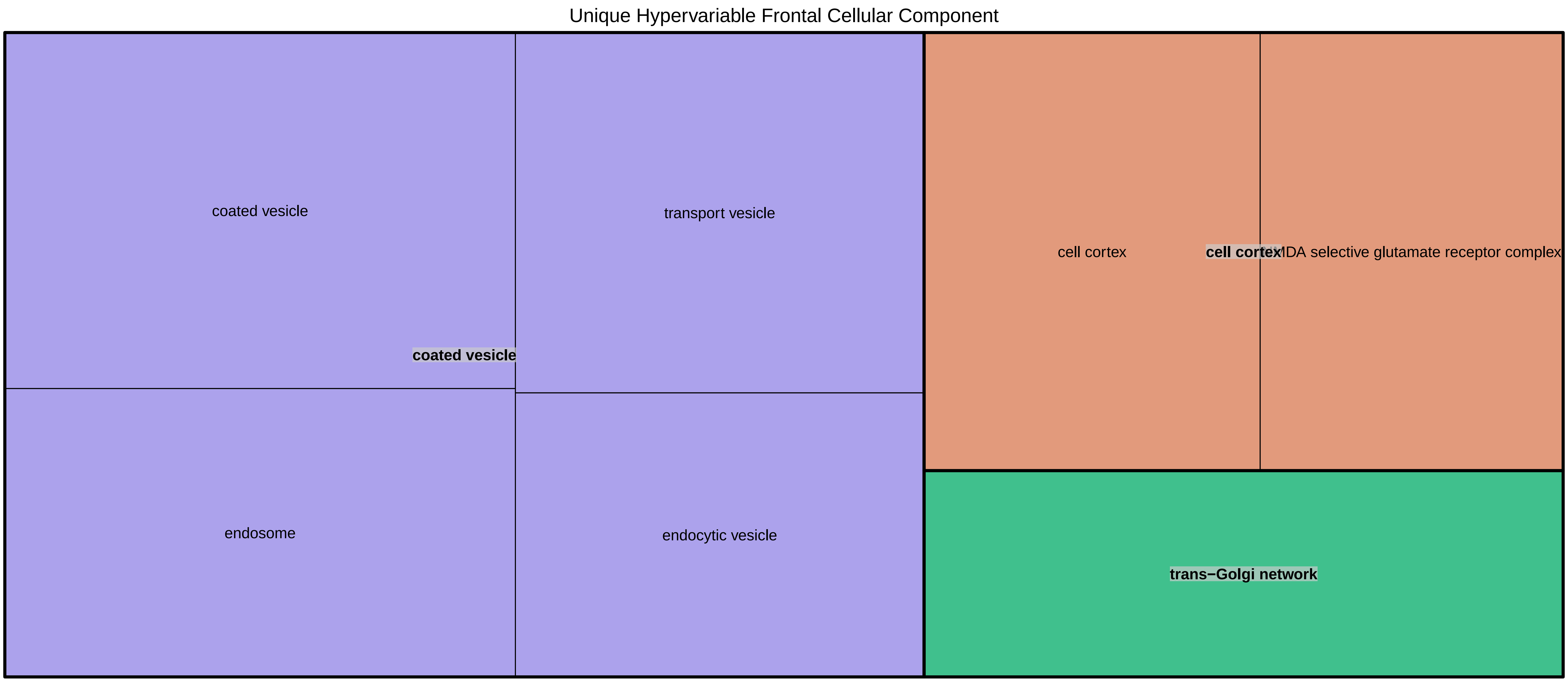
Unique Hypervariable Frontal Cellular Component
coated vesicle
coated vesicle
endosome
transport vesicle
endocytic vesicle
cell cortex
cell cortex
NMDA selective glutamate receptor complex
trans−Golgi network

Unique Hypovariable Breast Cellular Component

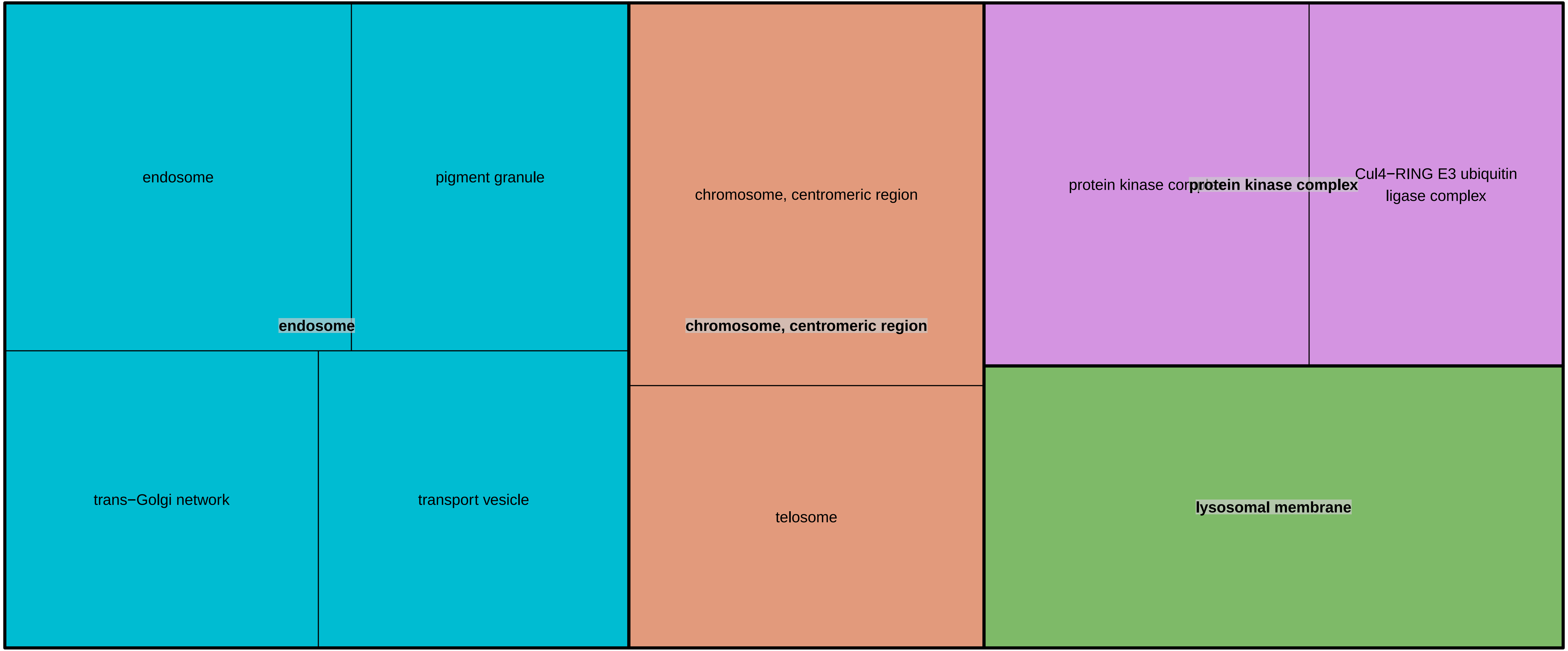

Unique Hypovariable Cerebellum Cellular Component

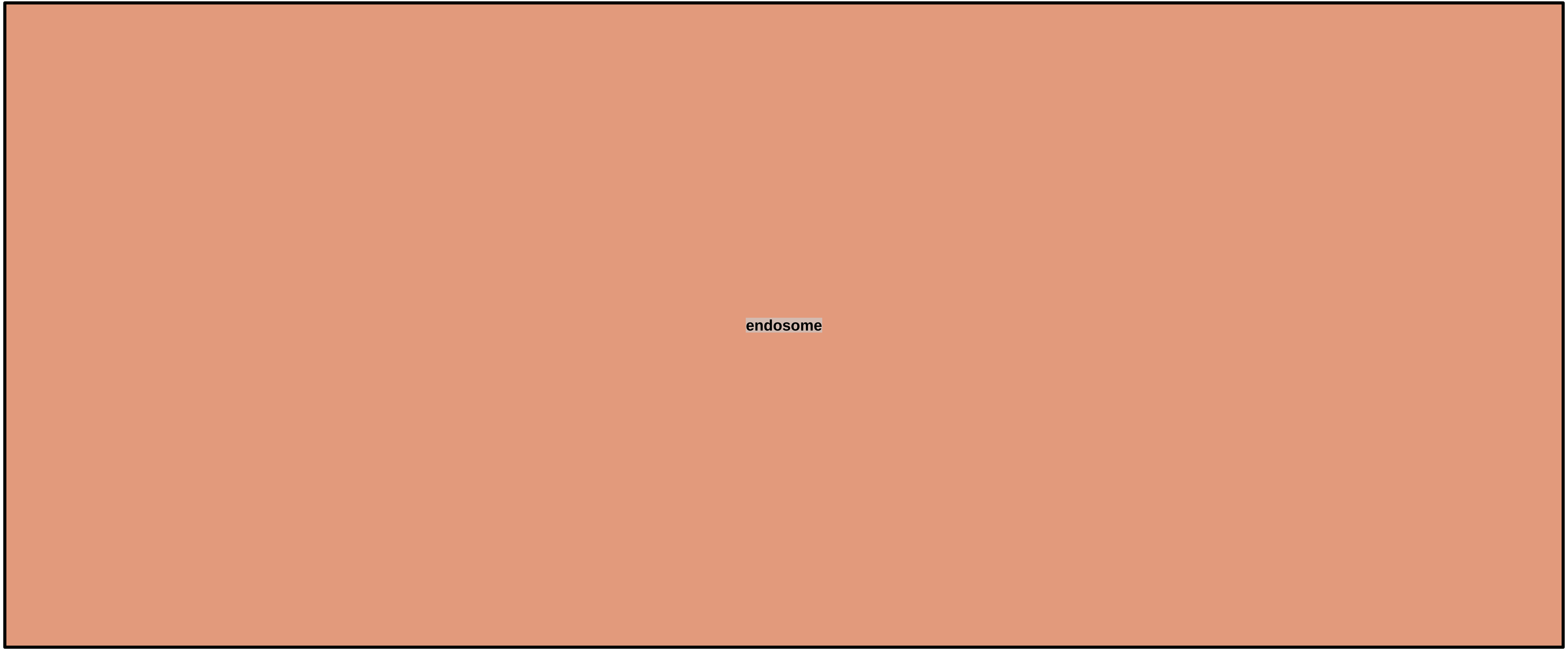

Unique Hypovariable Frontal Cellular Components

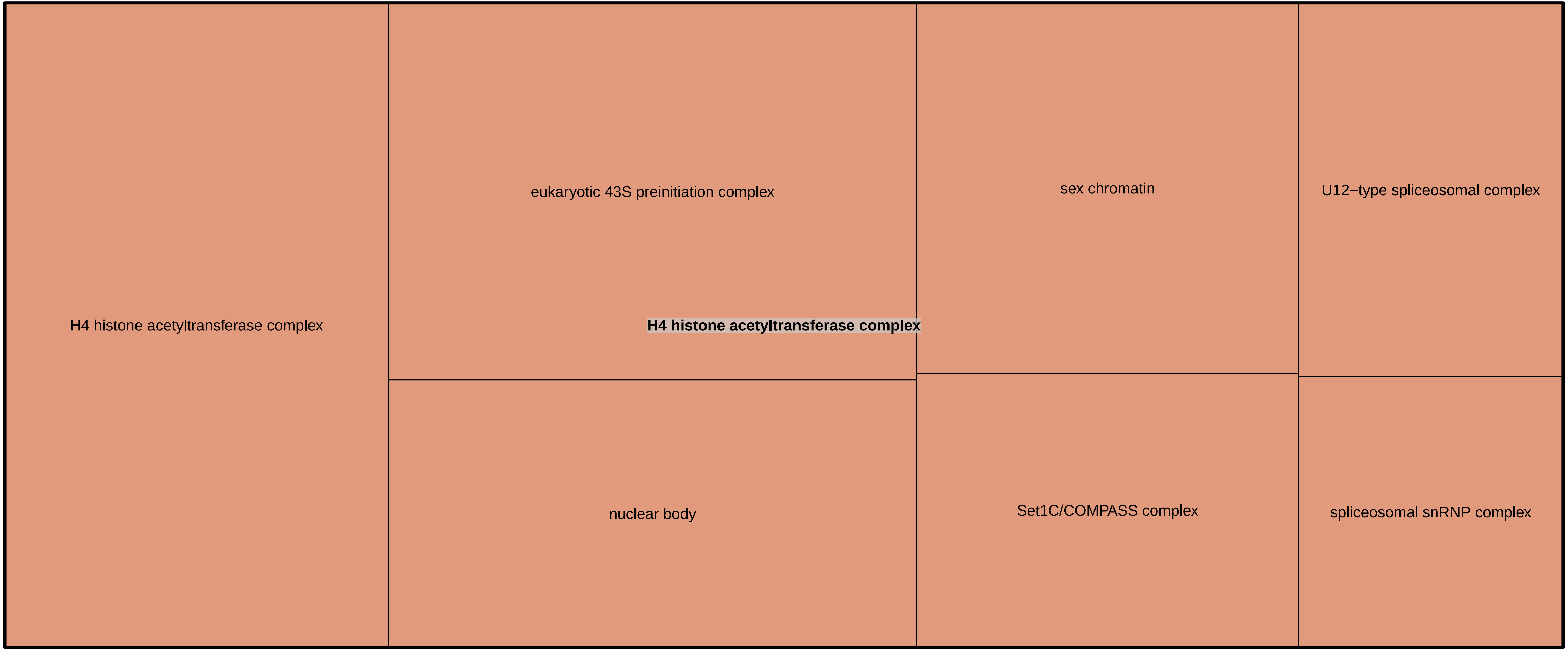

Supplement: Supplementary file 3 — Additional file 3. Complete list of GO term treemaps for all genes [file 12864_2019_6308_MOESM3_ESM.pdf]
