# Supplementary material for: Human gene expression variability and its dependence on methylation and aging
Source: BMC Genomics. 2019 Dec 7;20:941. doi: 10.1186/s12864-019-6308-7 (PMC6898959; doi:10.1186/s12864-019-6308-7)
Supplement: Supplementary file 4 — Additional file 4. Chi−Squared enrichment analysis methodology [file 12864_2019_6308_MOESM4_ESM.pdf]

Additional File 4. Chi−Squared Enrichment Analysis Methodology

1) Construct a contigency table of genes for each tissue type
2) Conduct a chi−square test using the chisq.test() function in R
3) Extract the standardized residuals(stdres) whereby:

> standardized residuals, (observed − expected) / sqrt(V), where V is the
> residual cell variance (Agresti, 2007, section 2.4.5 for the case where
> x is a matrix, n * p * (1 − p) otherwise).

Breast Tissue Essential Gene Enrichment Analysis
Contingency Table

	Hypervariable	Hypovariable	NV	
**Essential**	165	103	2095	2363
**Non−Essential**	1283	854	37452	39589
	1448	957	39547	41952

Chi−Squared Test Summary

X−Squared	Degrees of Freedom	P−Value
146.198632787005	2	1.79213309275818e−32

Standardized Residuals

	Hypervariable	Hypovariable	NV
**Essential**	9.679	6.964	−12.073
**Non−Essential**	−9.679	−6.964	12.073

Cerebellum Tissue Essential Gene Enrichment Analysis
Contingency Table

	Hypervariable	Hypovariable	NV	
**Essential**	160	76	2128	2364
**Non−Essential**	1480	761	37357	39598
	1640	837	39485	41962

Chi−Squared Test Summary

X−Squared	Degrees of Freedom	P−Value
75.557486782899	2	3.91651876644079e−17

Standardized Residuals

	Hypervariable	Hypovariable	NV
**Essential**	7.386	4.368	−8.665
**Non−Essential**	−7.386	−4.368	8.665

Frontal Cortex Tissue Essential Gene Enrichment Analysis
Contingency Table

	Hypervariable	Hypovariable	NV	
**Essential**	181	121	2062	2364
**Non−Essential**	1579	1133	36886	39598
	1760	1254	38948	41962

Chi−Squared Test Summary

X−Squared	Degrees of Freedom	P−Value
118.069557050356	2	2.29890723006322e−26

Standardized Residuals

	Hypervariable	Hypovariable	NV
**Essential**	8.645	6.261	−10.840
**Non−Essential**	−8.645	−6.261	10.840

Cerebellum Tissue Methylation Enrichment Analysis
Contingency Table

	High Methylation	Low Methylation	Medium Methylation	
**Hypervariable**	183	626	186	995
**Hypovariable**	38	472	32	542
**NV**	2377	6015	1394	9786
	2598	7113	1612	11323

Chi−Squared Test Summary

X−Squared	Degrees of Freedom	P−Value
170.654433820031	4	7.5684732413822e−36

Standardized Residuals

	High Methylation	Low Methylation	Medium Methylation
**Hypervariable**	−3.57596	0.06529	4.21279
**Hypovariable**	−9.04103	11.97954	−5.68945
**NV**	8.59096	−7.52068	0.06401

Frontal Cortex Tissue Methylation Enrichment Analysis
Contingency Table

	High Methylation	Low Methylation	Medium Methylation	
**Hypervariable**	178	627	276	1081
**Hypovariable**	60	662	72	794
**NV**	2397	5355	1762	9514
	2635	6644	2110	11389

Chi−Squared Test Summary

X−Squared	Degrees of Freedom	P−Value
280.693146499535	4	1.57958487900589e−59

Standardized Residuals

	High Methylation	Low Methylation	Medium Methylation
**Hypervariable**	−5.46631	−0.23494	6.23142
**Hypovariable**	−10.79327	14.83749	−7.11254
**NV**	11.73222	−10.00349	−0.04067
