# Supplementary material for: Human gene expression variability and its dependence on methylation and aging
Source: BMC Genomics. 2019 Dec 7;20:941. doi: 10.1186/s12864-019-6308-7 (PMC6898959; doi:10.1186/s12864-019-6308-7)

Additional File 5. Complete list of GO terms for essential genes

Common Hypervariable Essential GO Annotations

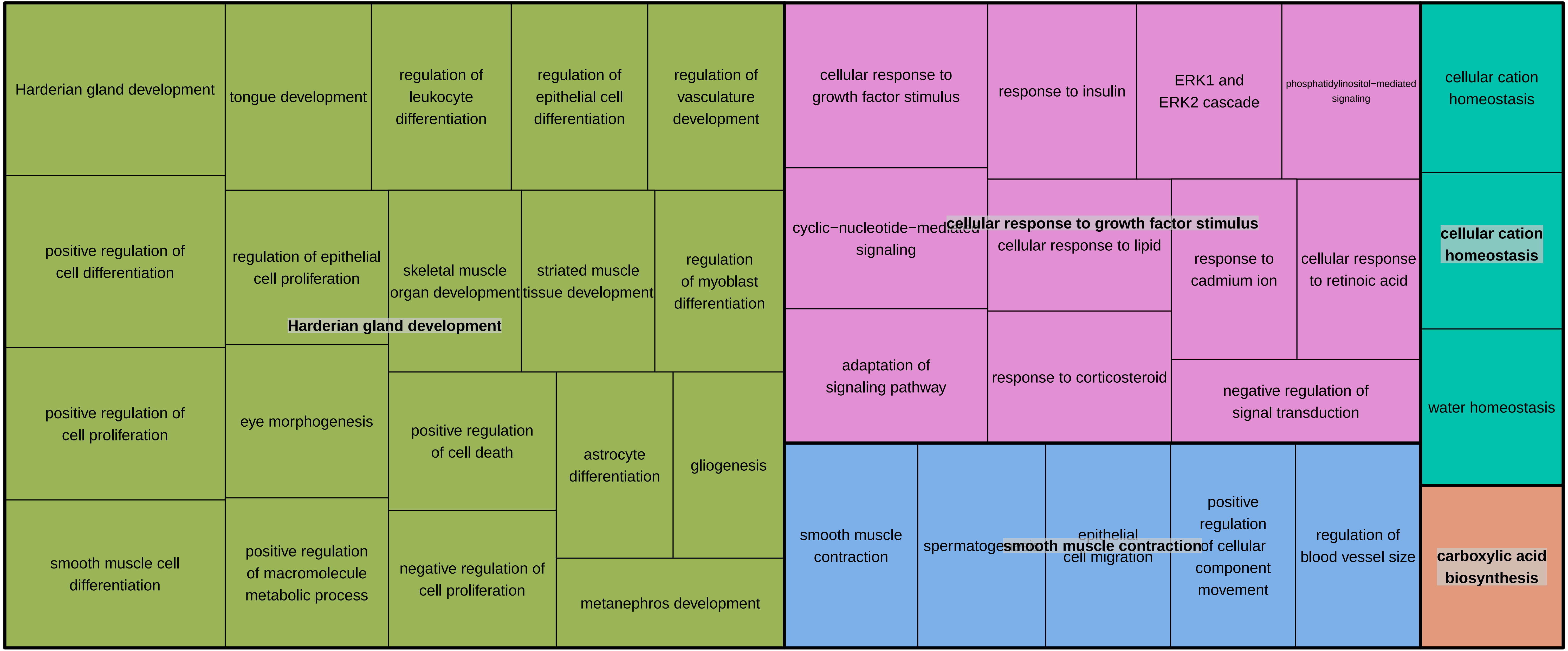

Hypervariable Essential GO Annotations in Breast Tissue

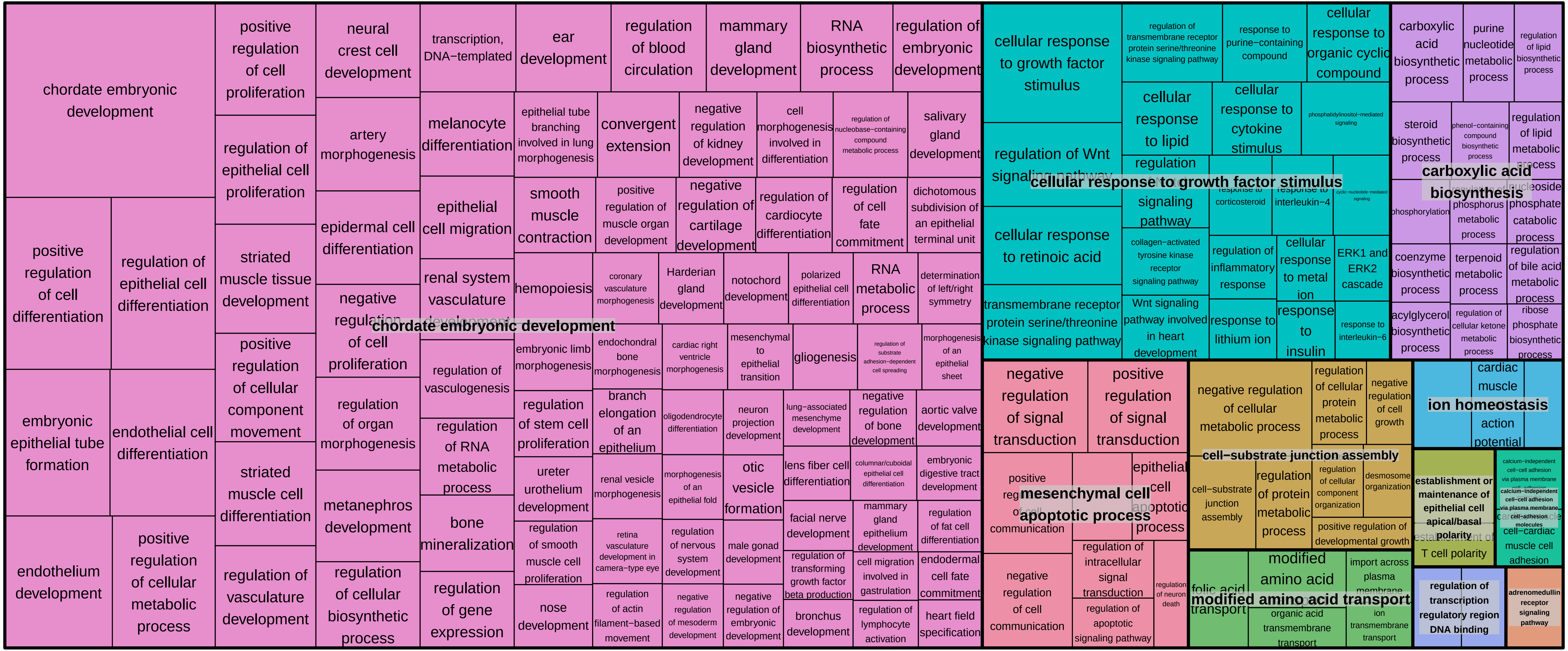

Hypervariable Essential GO Annotations in Cerebellum Tissue

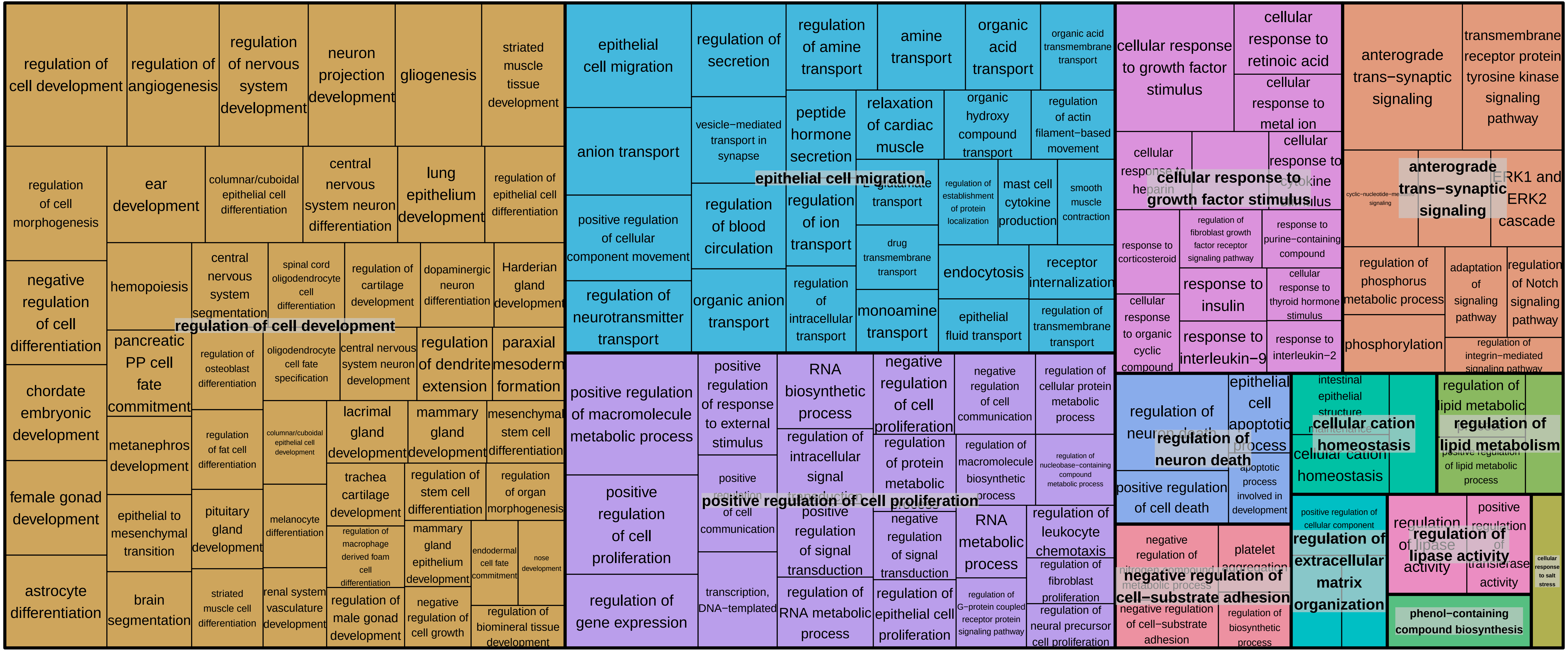

Hypervariable Essential GO Annotations in Frontal Cortex Tissue

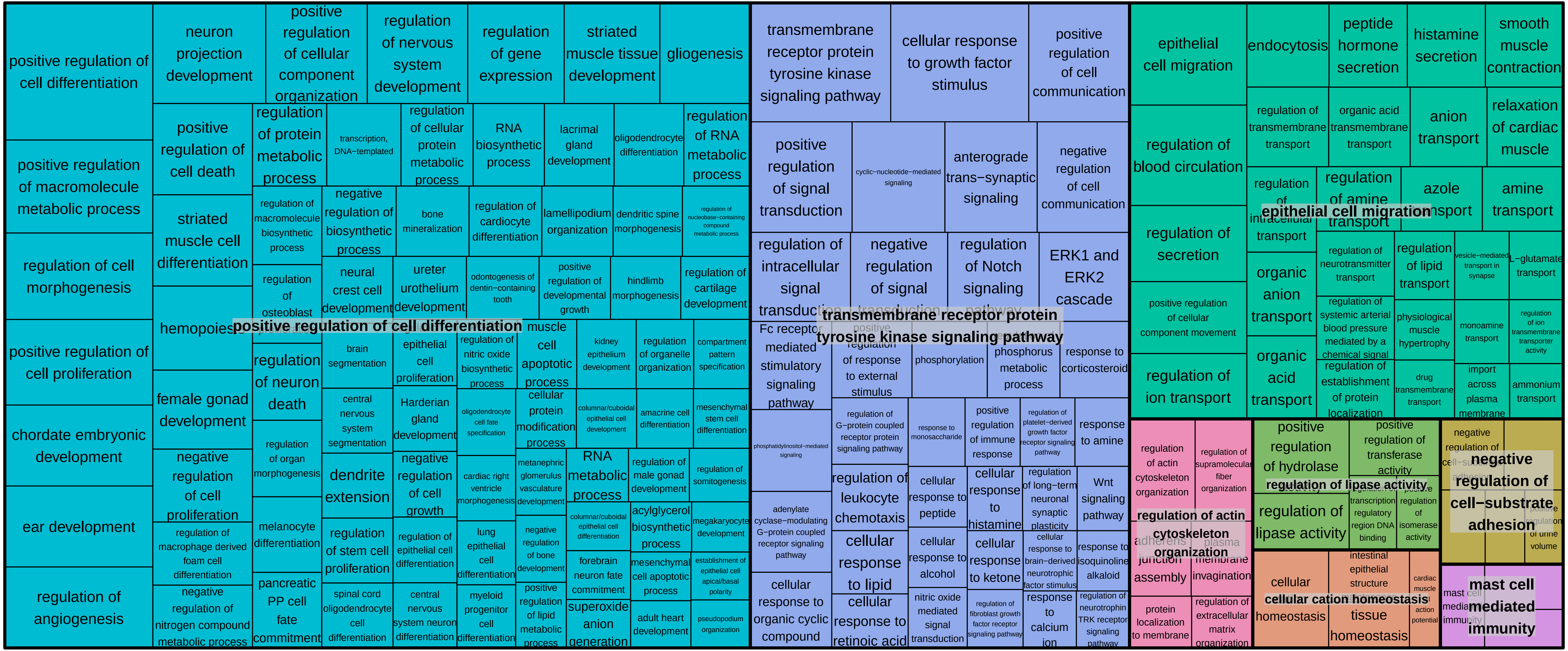

Common Hypovariable Essential GO Annotations

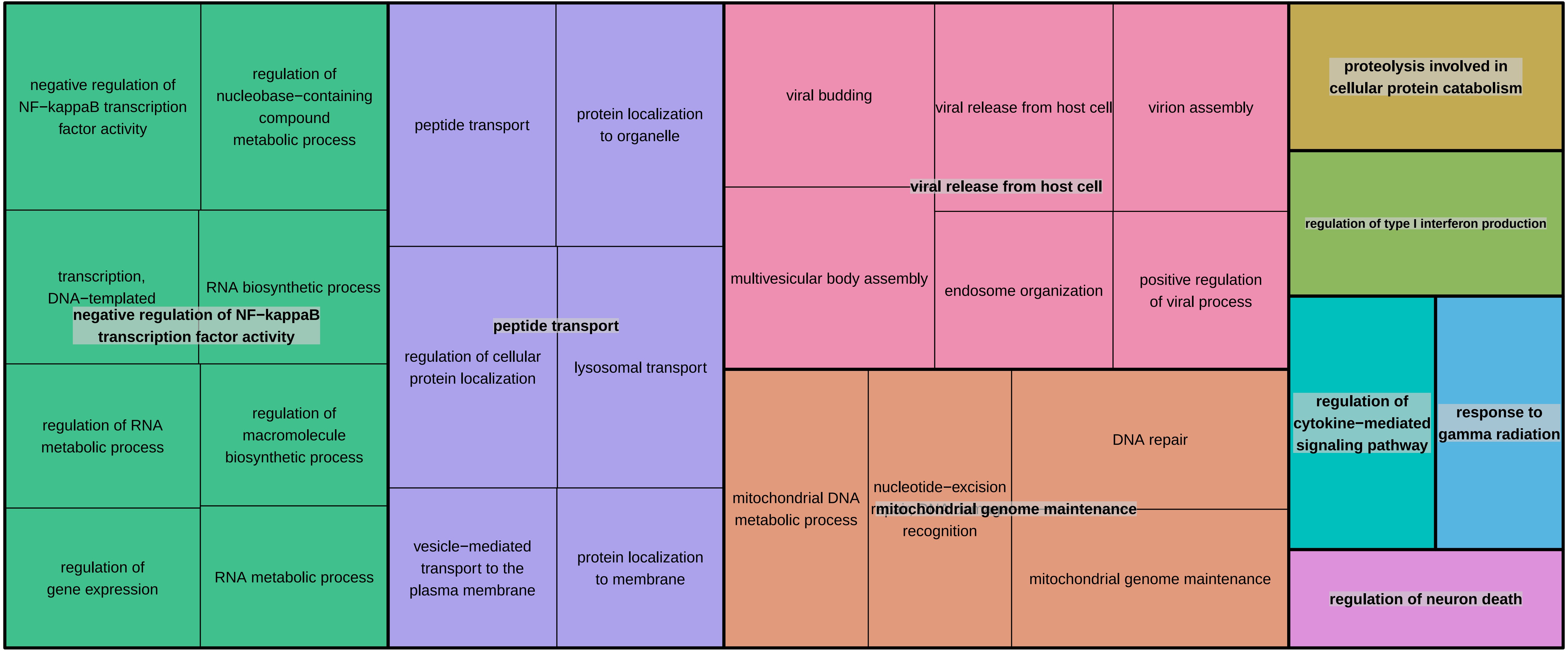

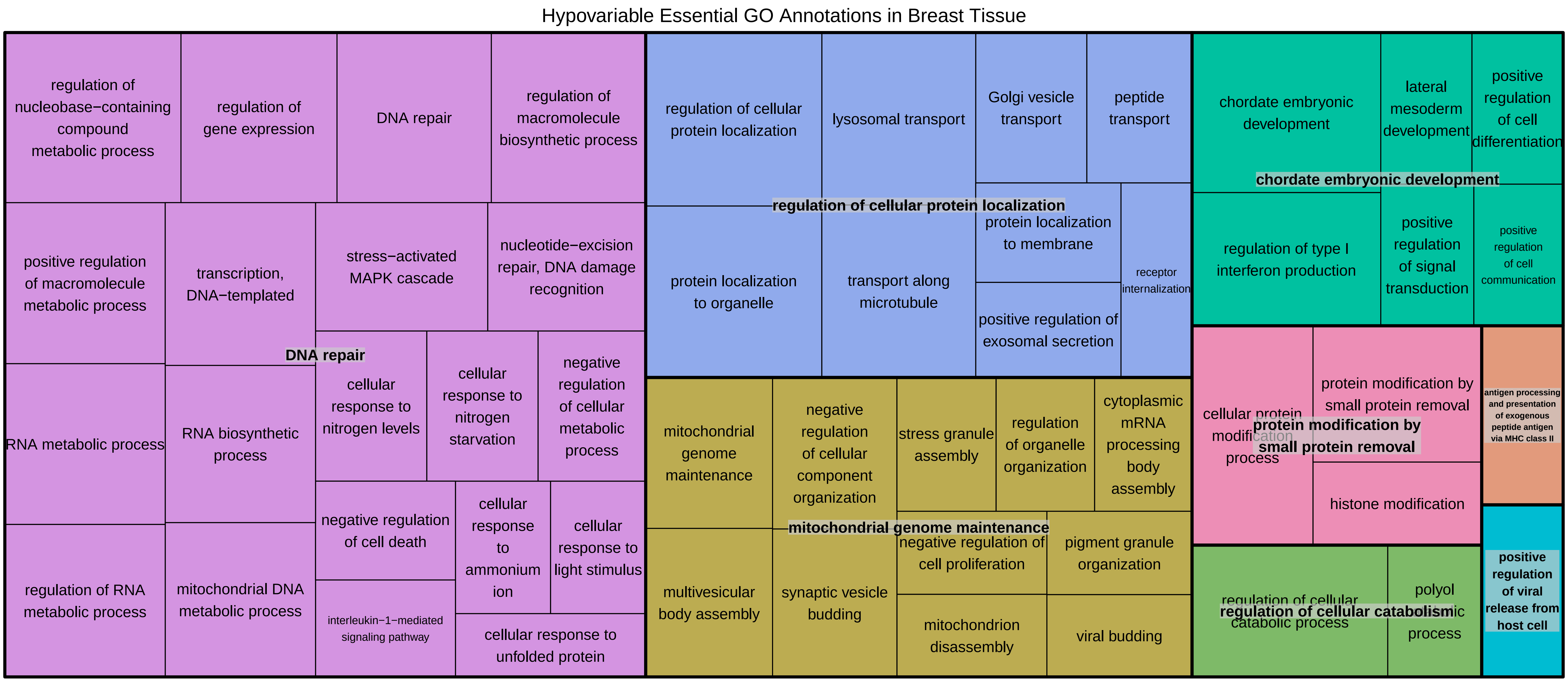

Hypovariable Essential GO Annotations in Breast Tissue
regulation of nucleobase−containing compound metabolic process
regulation of gene expression
DNA repair
regulation of macromolecule biosynthetic process
positive regulation of macromolecule metabolic process
transcription, DNA−templated
stress−activated MAPK cascade
nucleotide−excision repair, DNA damage recognition
DNA repair
cellular response to nitrogen levels
cellular response to nitrogen starvation
negative regulation of cellular metabolic process
RNA metabolic process
RNA biosynthetic process
negative regulation of cell death
cellular response to ammonium ion
cellular response to light stimulus
interleukin−1−mediated signaling pathway
regulation of RNA metabolic process
mitochondrial DNA metabolic process
cellular response to unfolded protein
regulation of cellular protein localization
lysosomal transport
Golgi vesicle transport
peptide transport
regulation of cellular protein localization
protein localization to membrane
receptor internalization
protein localization to organelle
transport along microtubule
positive regulation of exosomal secretion
mitochondrial genome maintenance
negative regulation of cellular component organization
stress granule assembly
regulation of organelle organization
cytoplasmic mRNA processing body assembly
mitochondrial genome maintenance
negative regulation of cell proliferation
pigment granule organization
multivesicular body assembly
synaptic vesicle budding
mitochondrion disassembly
viral budding
chordate embryonic development
lateral mesoderm development
positive regulation of cell differentiation
chordate embryonic development
regulation of type I interferon production
positive regulation of signal transduction
positive regulation of cell communication
cellular protein modification process
protein modification by small protein removal
protein modification by small protein removal
histone modification
antigen processing and presentation of exogenous peptide antigen via MHC class II
regulation of cellular catabolic process
regulation of cellular catabolism
polyol catabolic process
positive regulation of viral release from host cell

Hypovariable Essential GO Annotations in Cerebellum Tissue

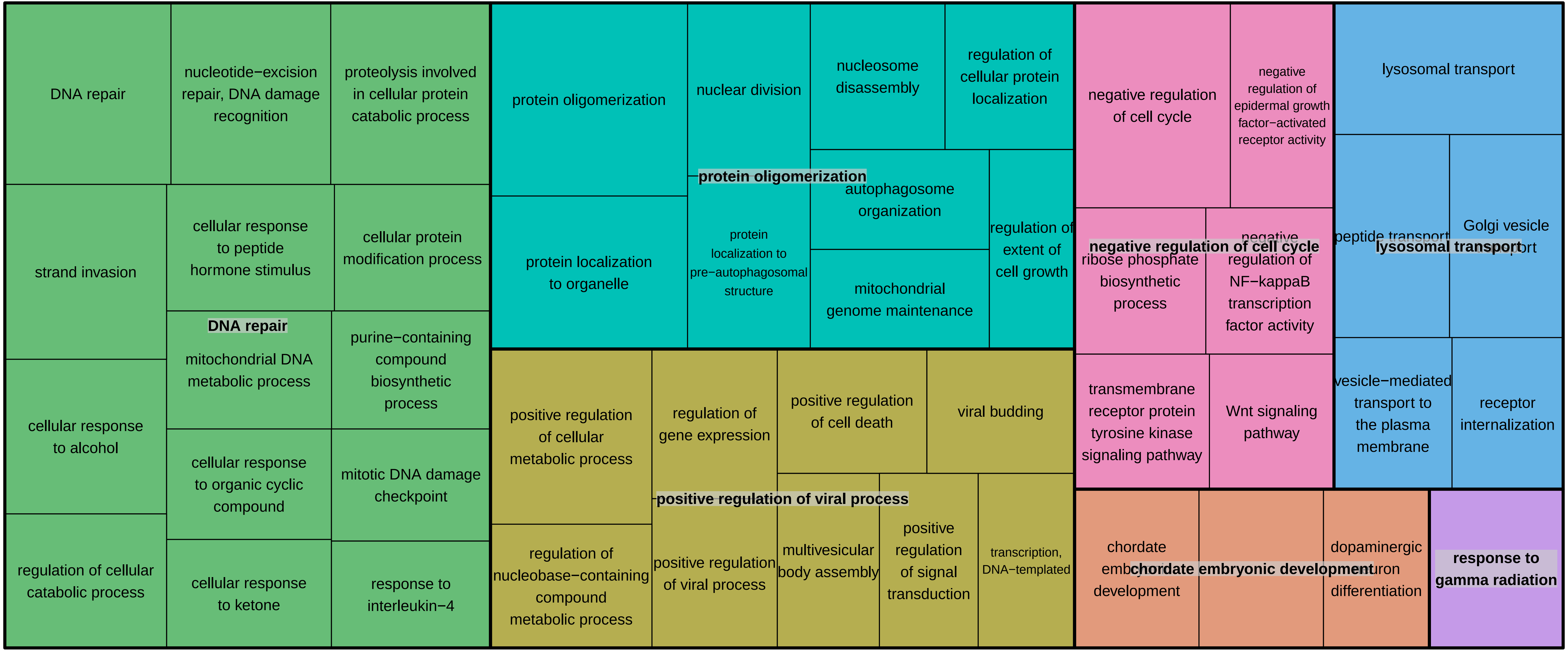

Hypovariable Essential GO Annotations in Frontal Cortex Tissue

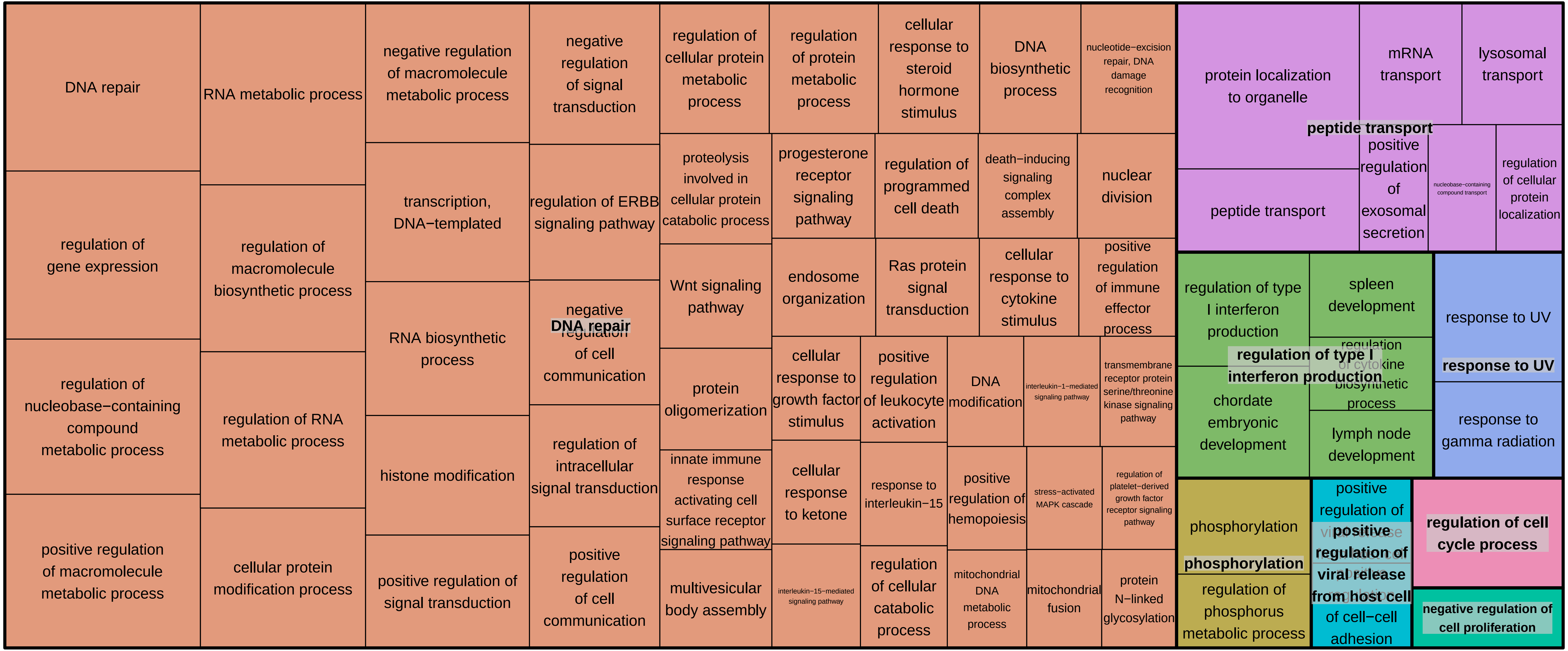

Supplement: Supplementary file 5 — Additional file 5. Complete list of GO term treemaps for essential genes [file 12864_2019_6308_MOESM5_ESM.pdf]
