# Supplementary material for: Human gene expression variability and its dependence on methylation and aging
Source: BMC Genomics. 2019 Dec 7;20:941. doi: 10.1186/s12864-019-6308-7 (PMC6898959; doi:10.1186/s12864-019-6308-7)

Additional File 6. Complete list of GO terms for age−regulated Hyper−variable genes

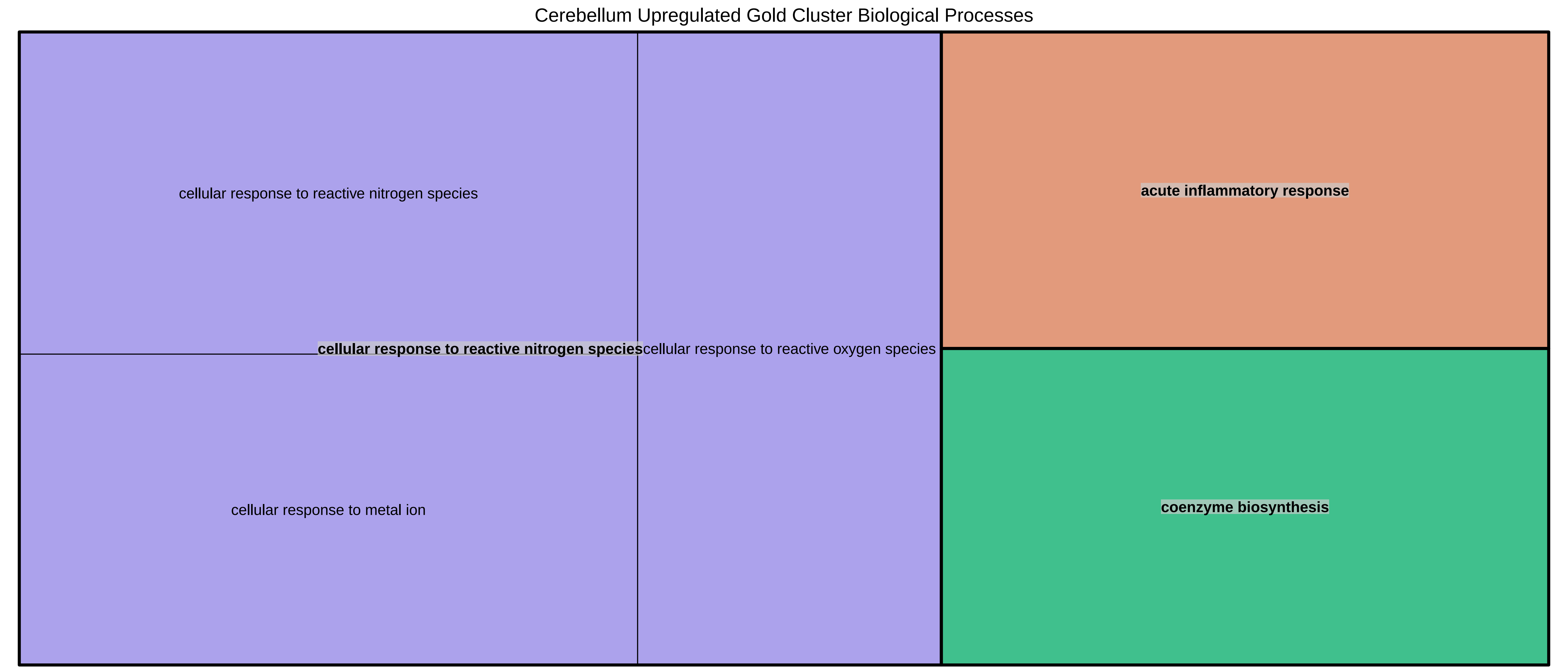
Cerebellum Upregulated Gold Cluster Biological Processes
cellular response to reactive nitrogen species
cellular response to metal ion
cellular response to reactive nitrogen species
cellular response to reactive oxygen species
acute inflammatory response
coenzyme biosynthesis

Cerebellum Upregulated Darkorange Cluster Biological Processes

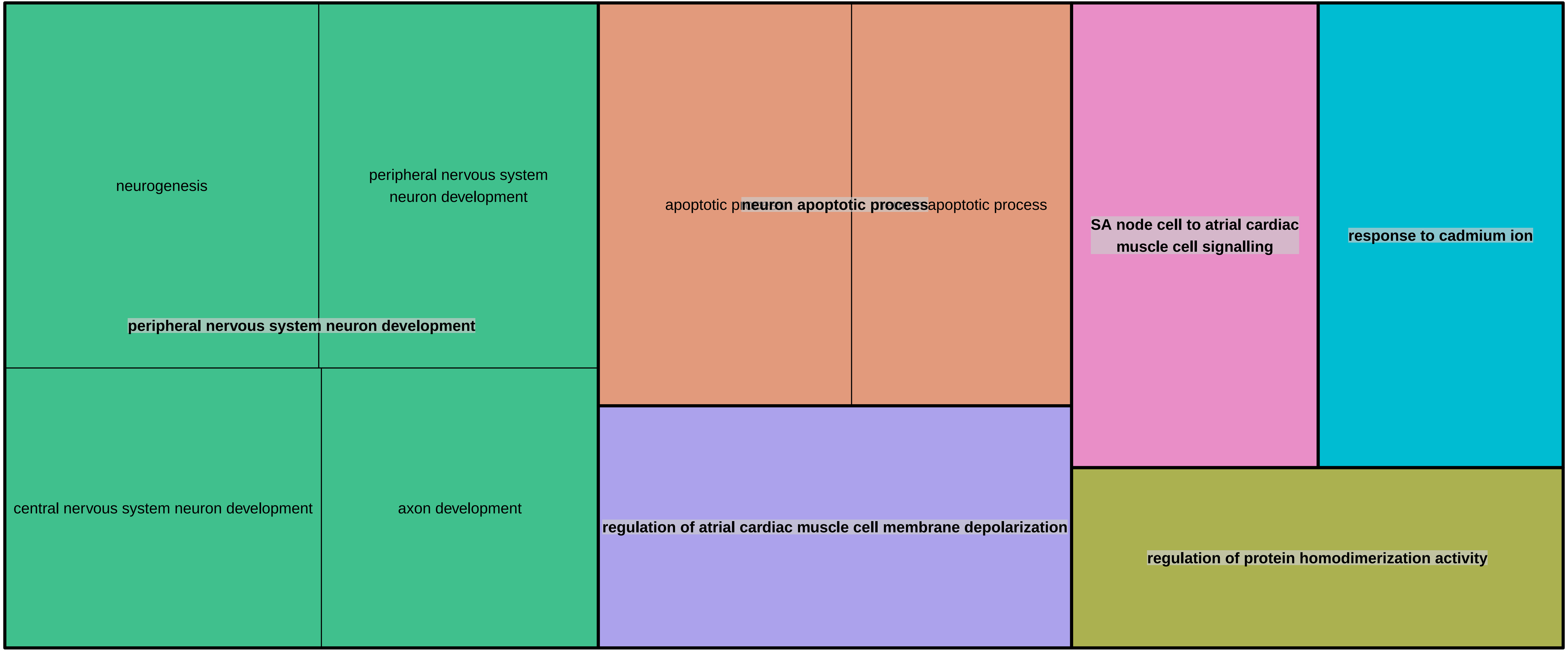

Cerebellum Downregulated Yellow Cluster Biological Processes

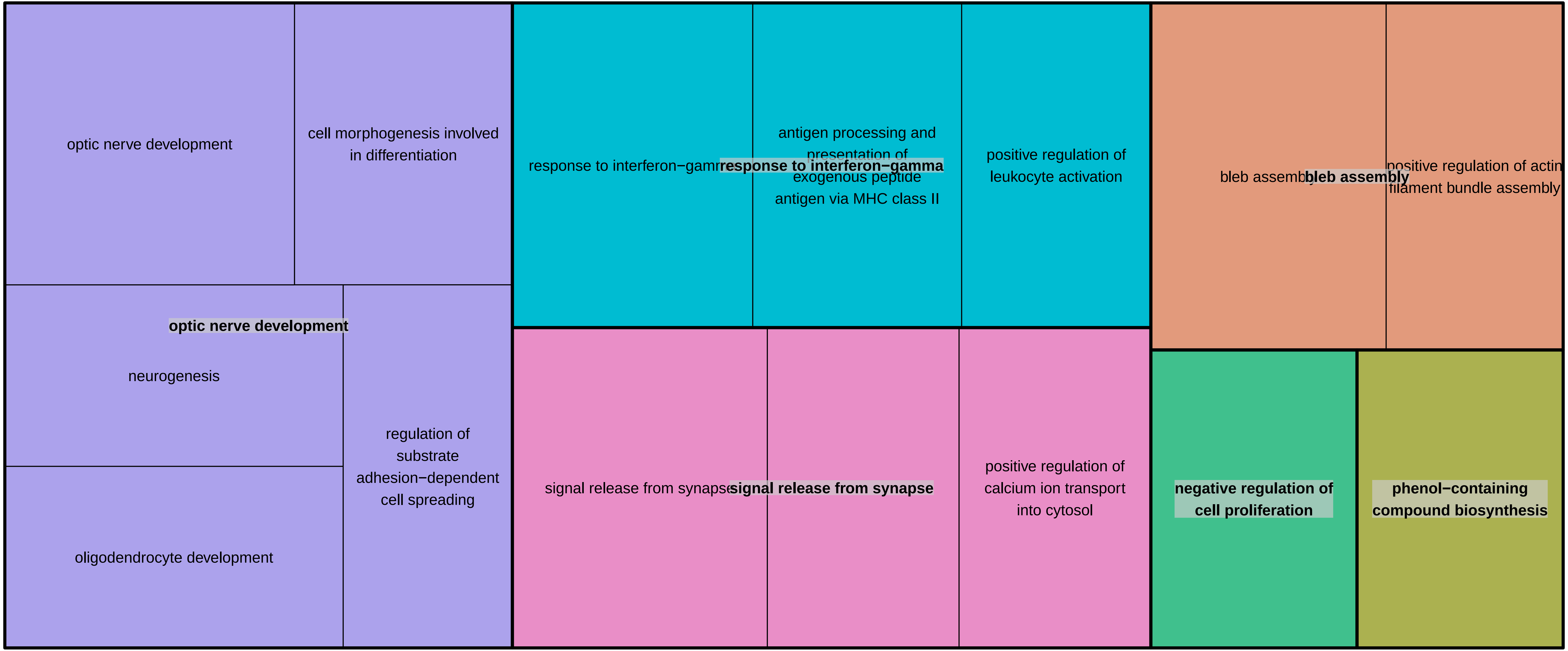

Cerebellum Downregulated Green Cluster Biological Processes

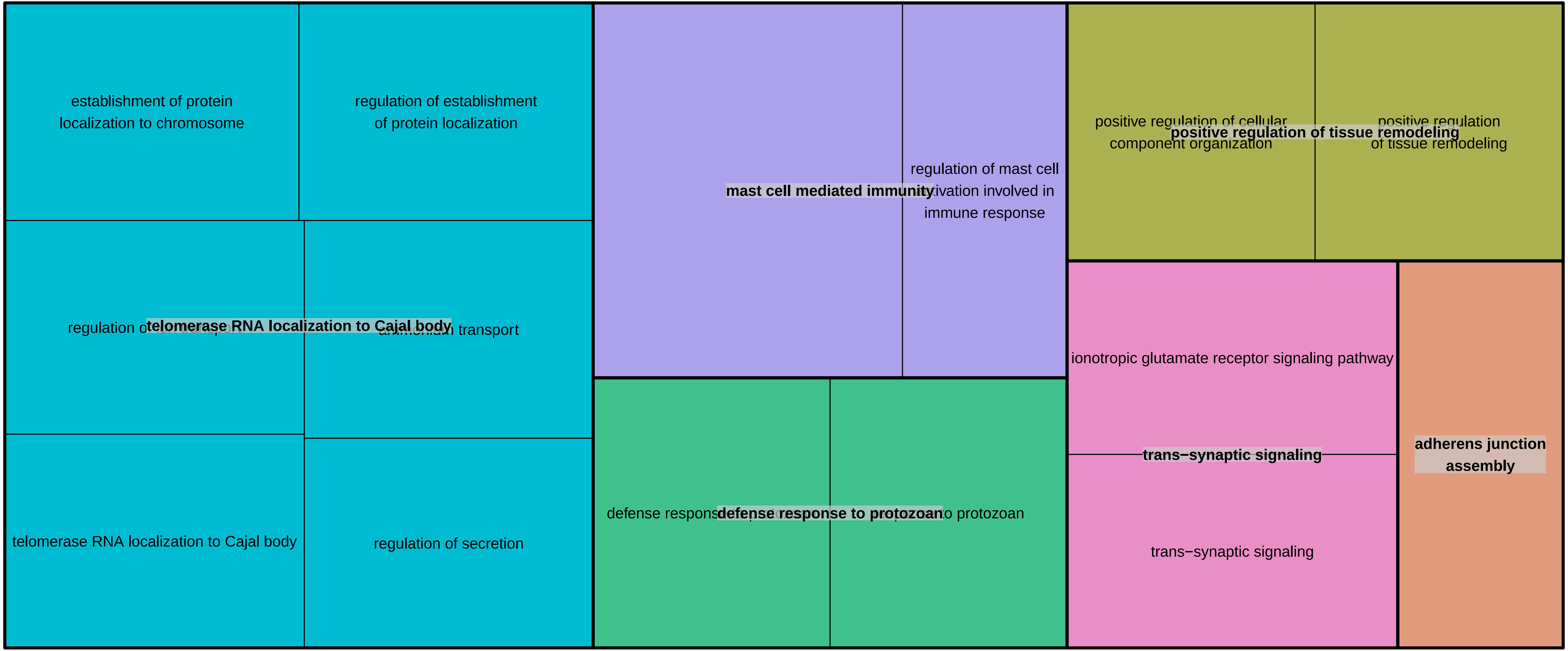

Frontal Cortex Upregulated Gold Cluster Biological Processes

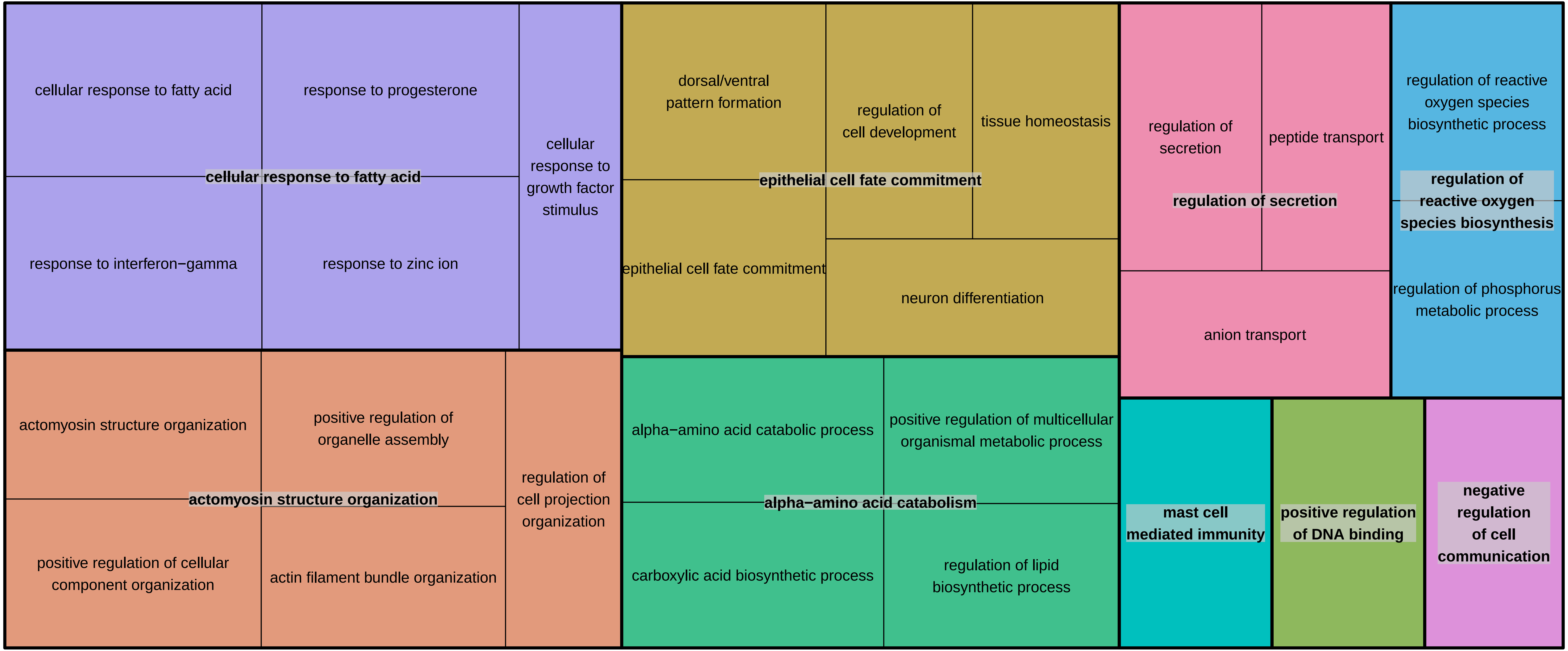

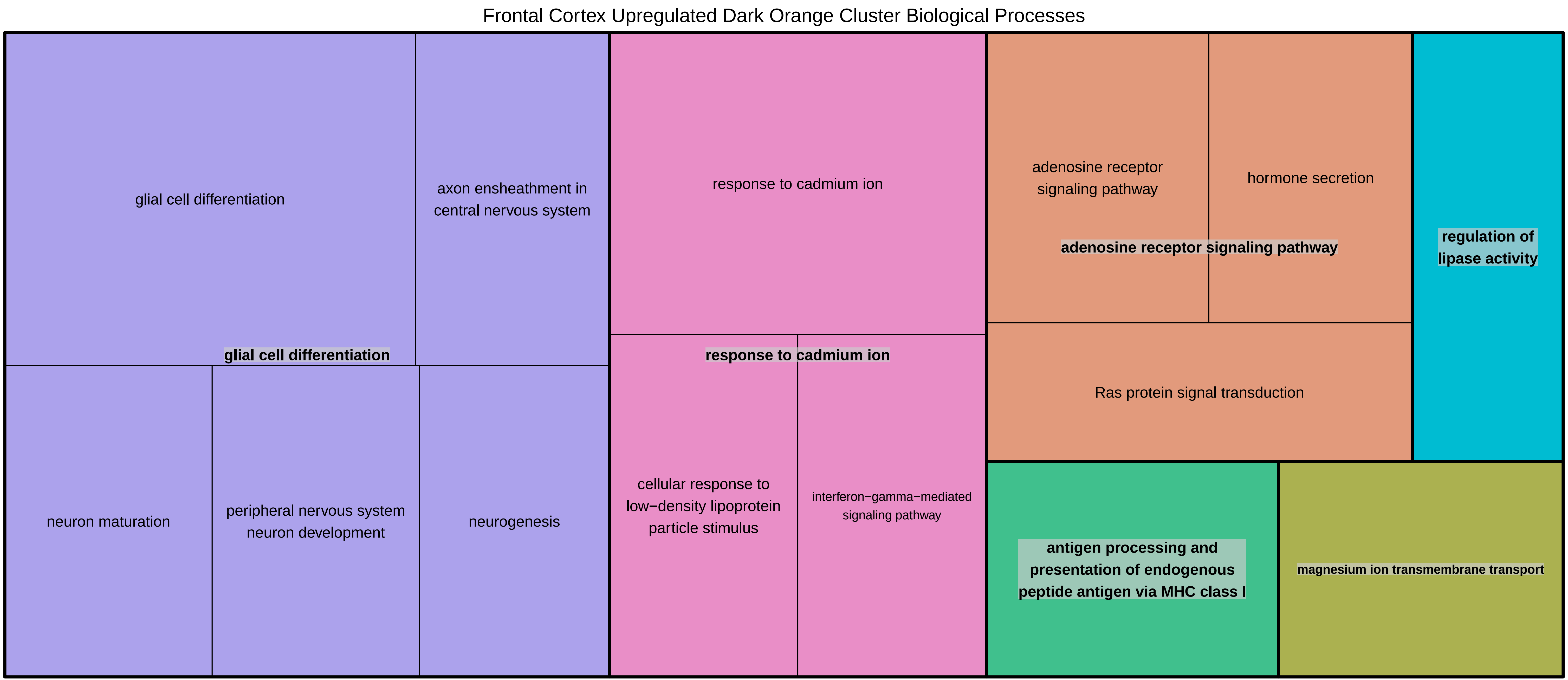
Frontal Cortex Upregulated Dark Orange Cluster Biological Processes
glial cell differentiation
axon ensheathment in central nervous system
glial cell differentiation
neuron maturation
peripheral nervous system neuron development
neurogenesis
response to cadmium ion
response to cadmium ion
cellular response to low−density lipoprotein particle stimulus
interferon−gamma−mediated signaling pathway
adenosine receptor signaling pathway
hormone secretion
adenosine receptor signaling pathway
Ras protein signal transduction
regulation of lipase activity
antigen processing and presentation of endogenous peptide antigen via MHC class I
magnesium ion transmembrane transport

Frontal Cortex Upregulated Yellow Cluster Biological Processes

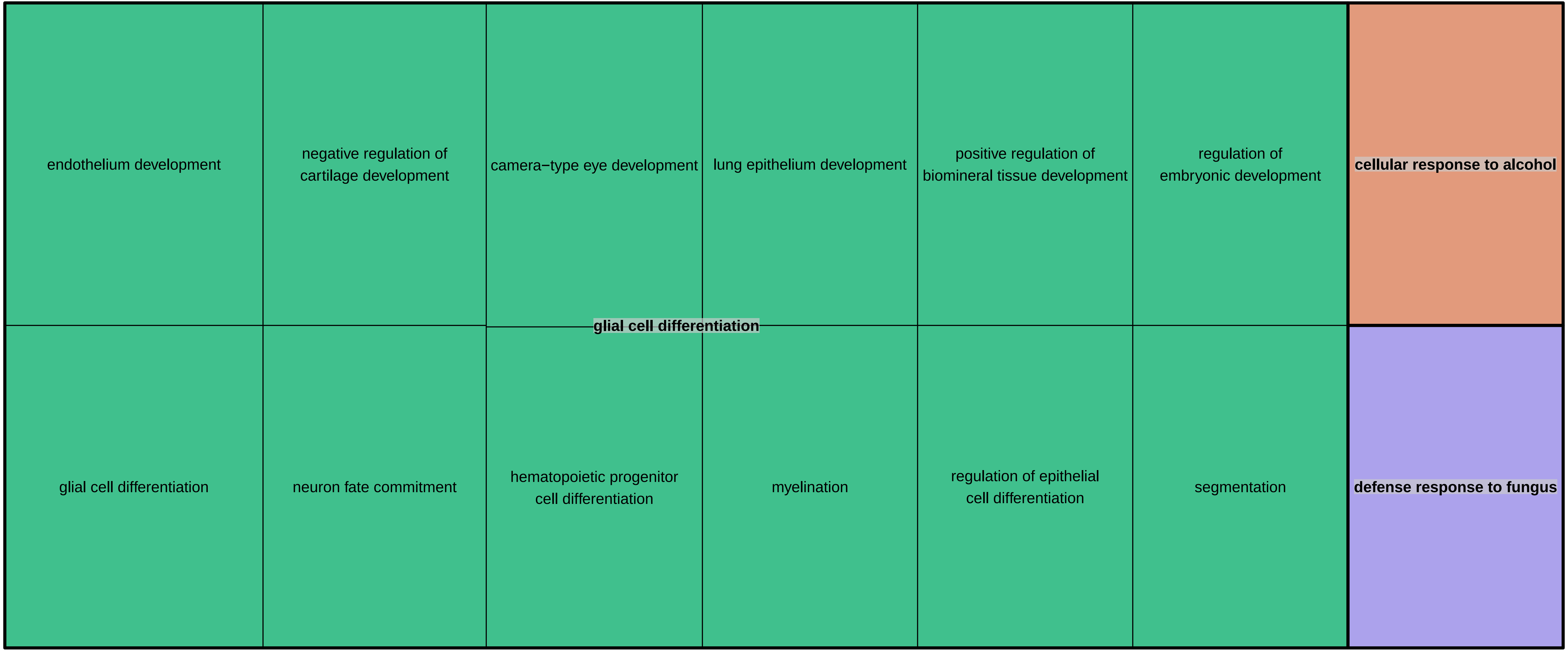

Frontal Cortex Downregulated Red Cluster Biological Processes

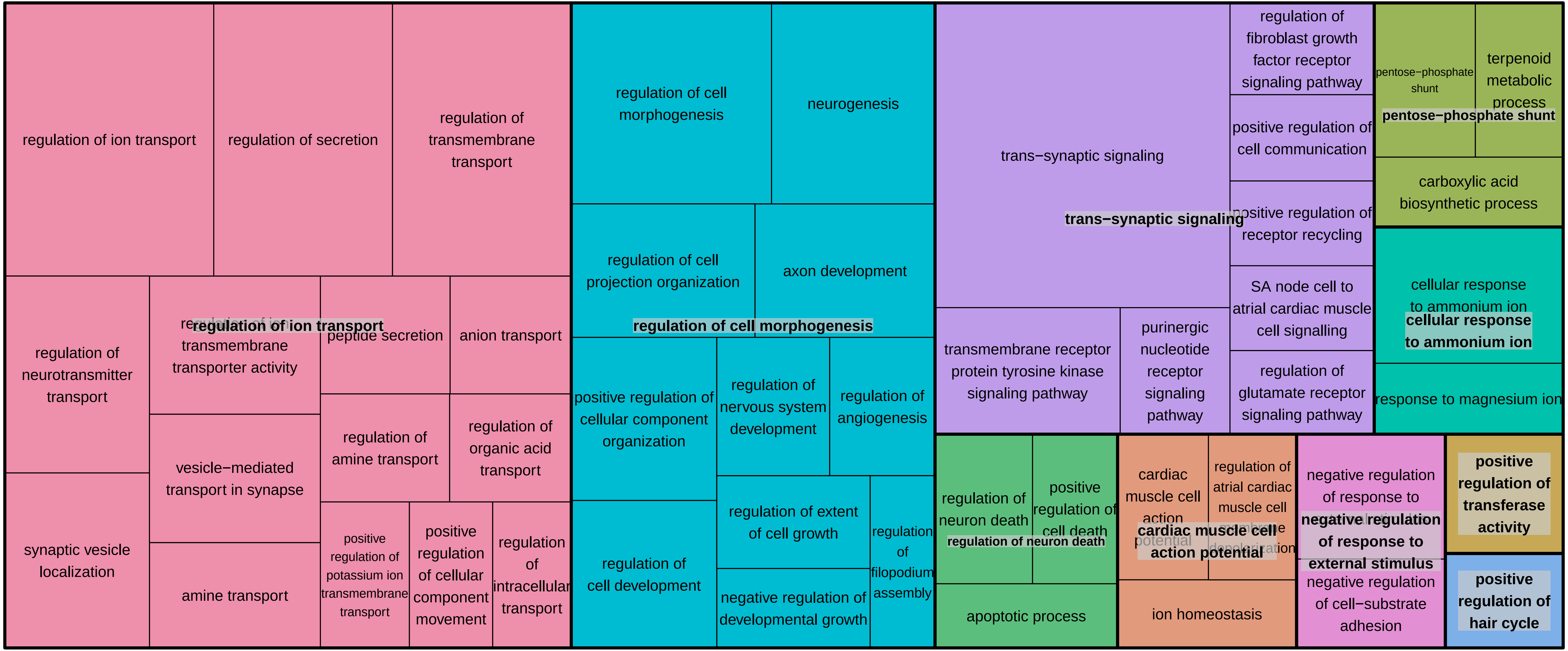

Cerebellum Upregulated Darkorange Cluster Cellular Component

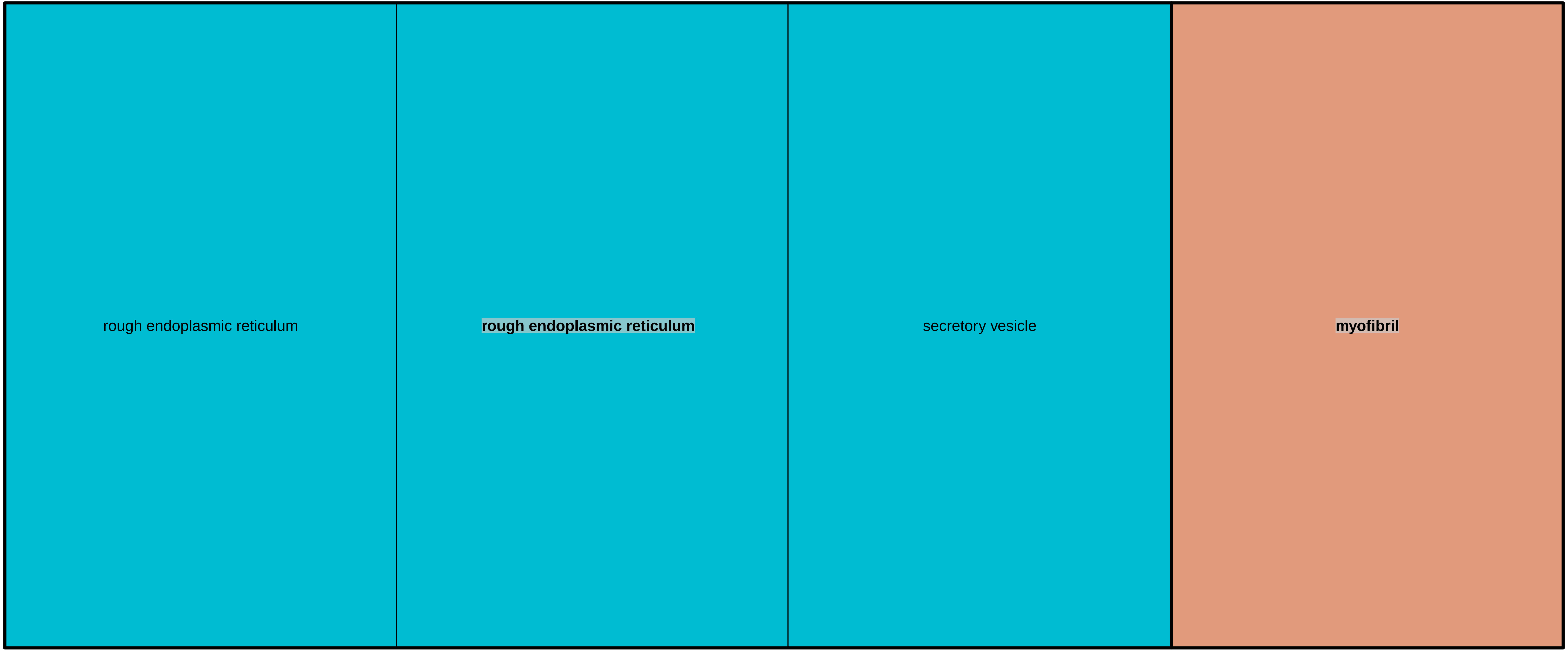

Cerebellum Downregulated Yellow Cluster Cellular Component

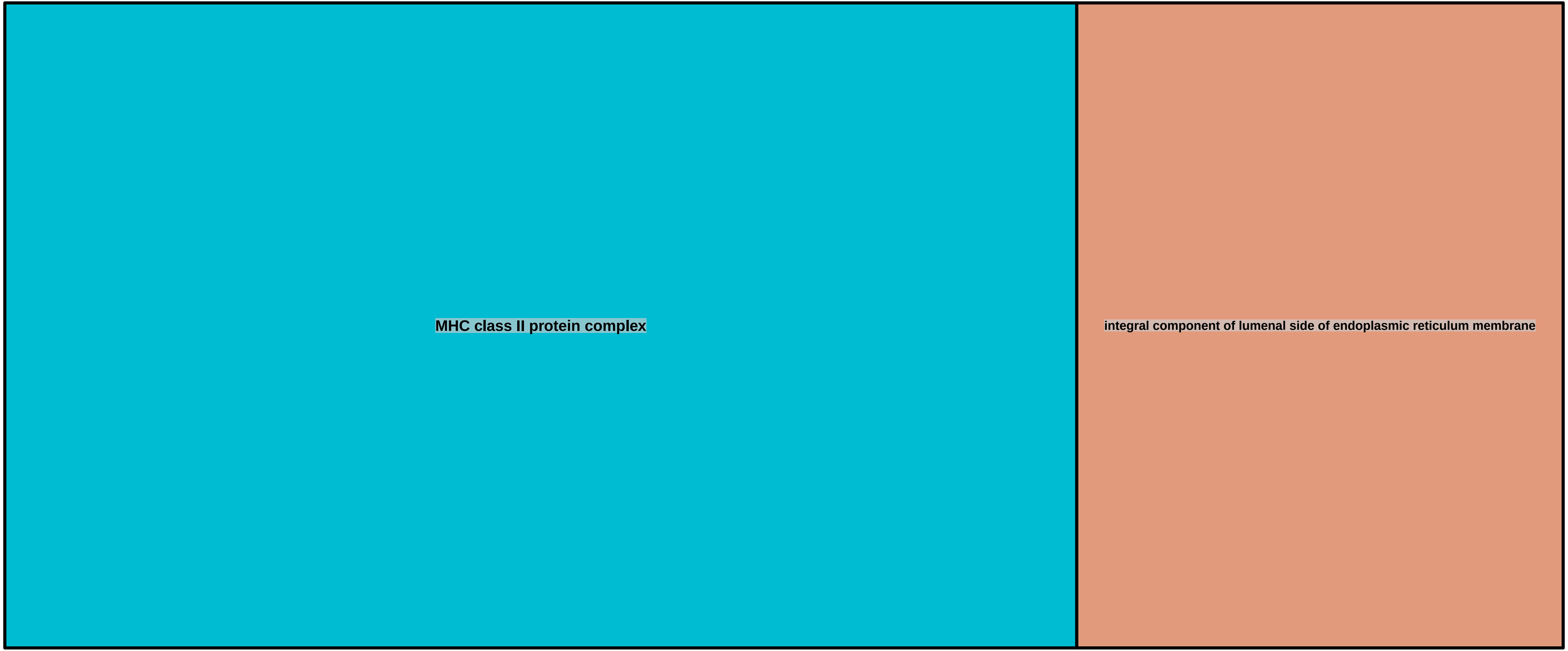

Cerebellum Downregulated Green Cluster Cellular Component

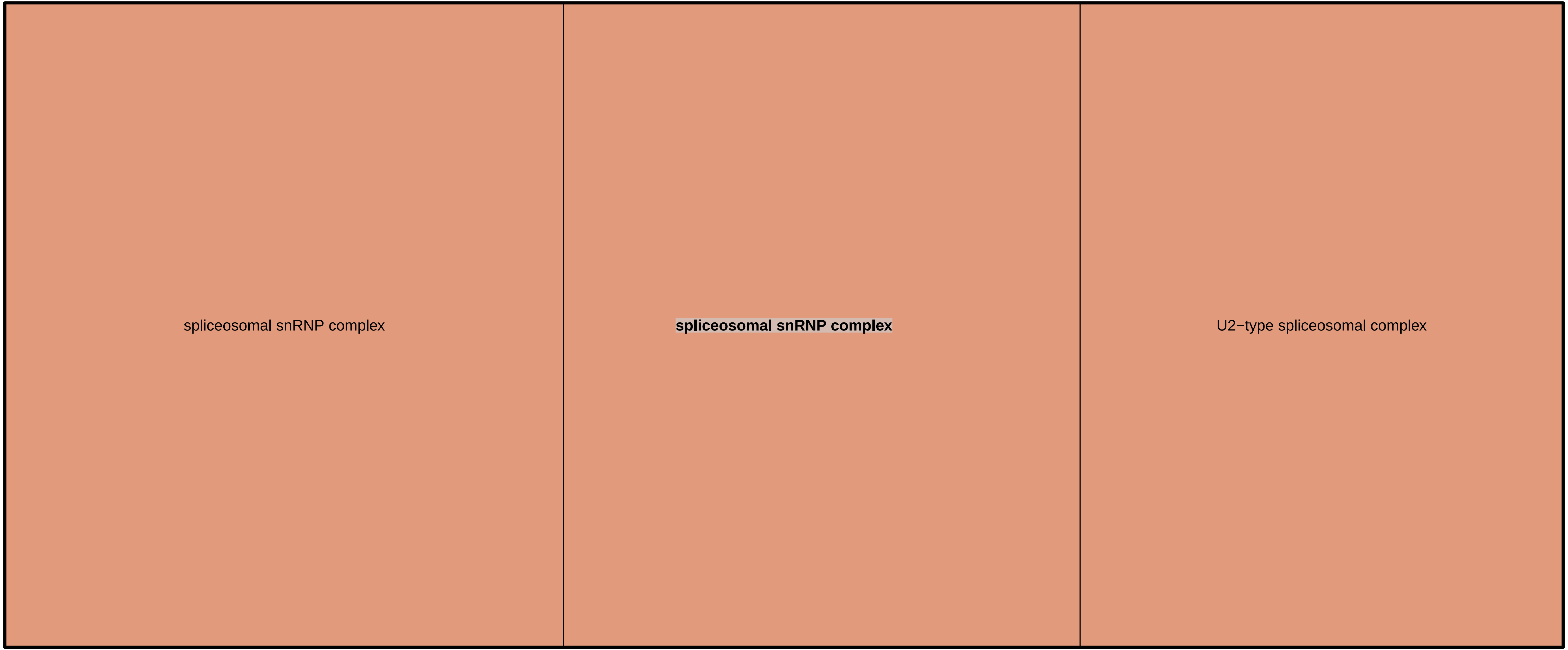

Frontal Cortex Upregulated Gold Cluster Cellular Component

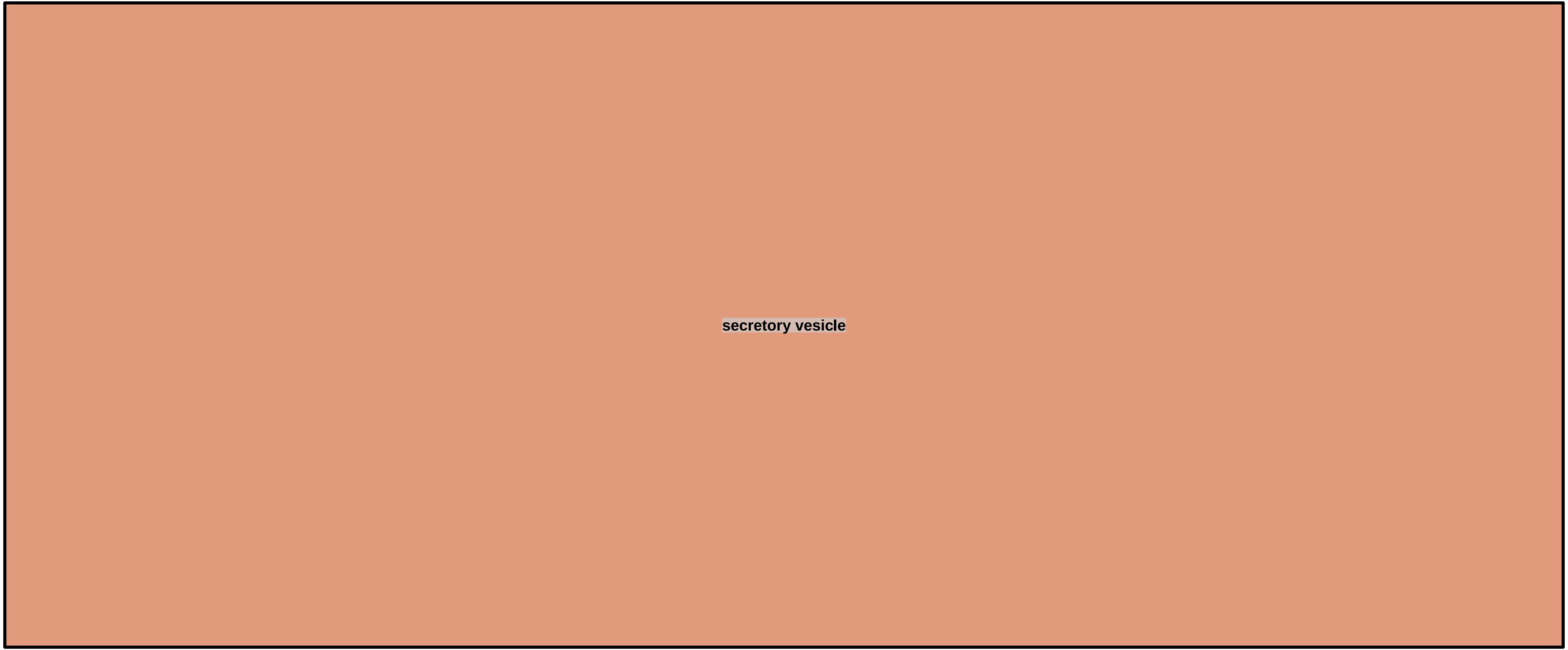

Frontal Cortex Upregulated Darkorange Cluster Cellular Component

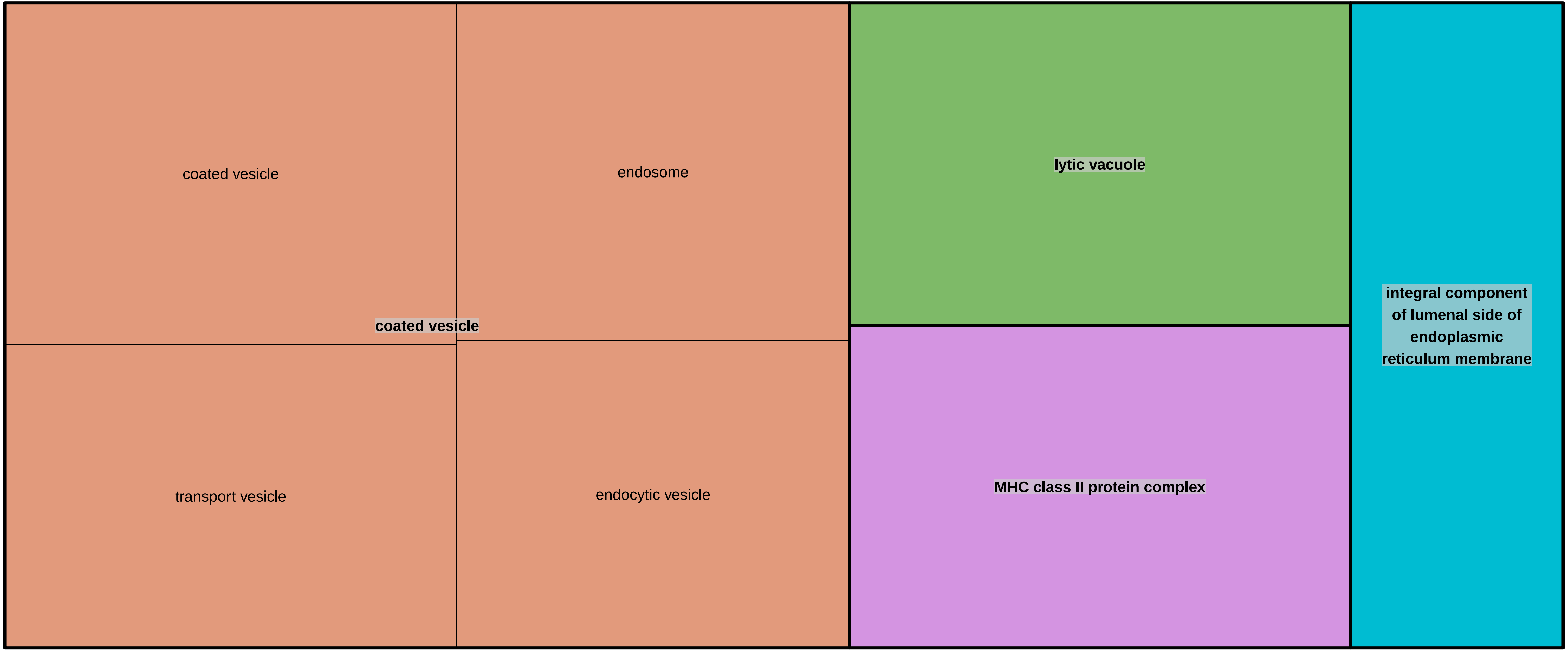

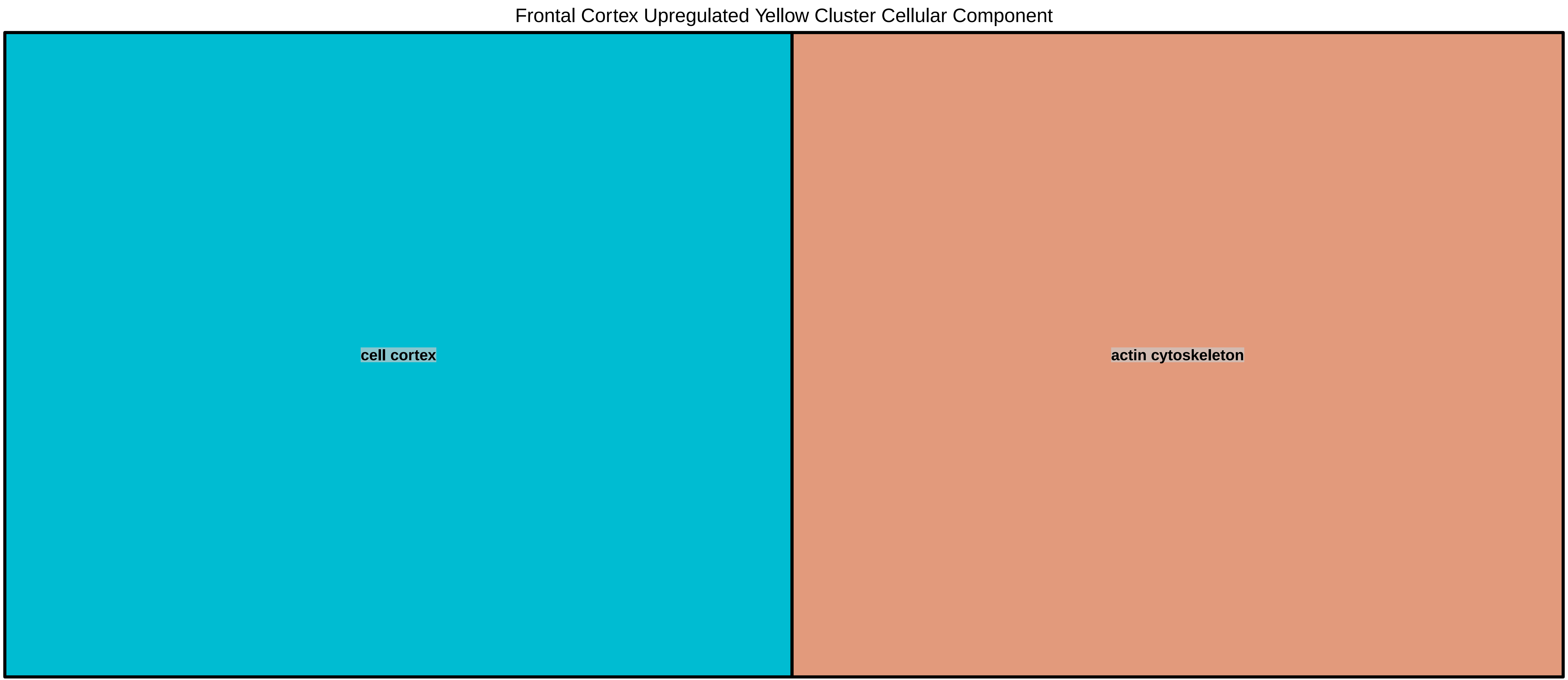
Frontal Cortex Upregulated Yellow Cluster Cellular Component
cell cortex
actin cytoskeleton

Frontal Cortex Downregulated Red Cluster Cellular Component

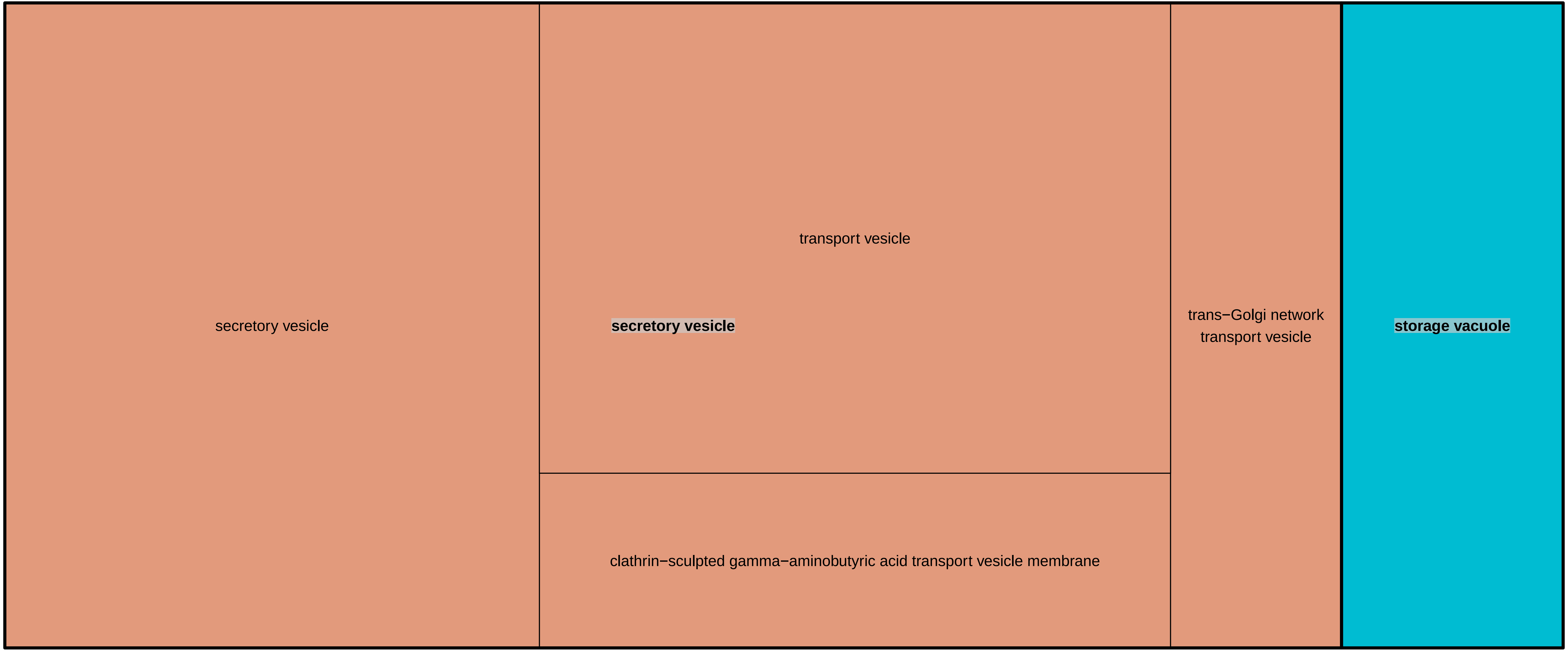

Supplement: Supplementary file 6 — Additional file 6. Complete list of GO term treemaps for age-regulated Hyper-Variable genes [file 12864_2019_6308_MOESM6_ESM.pdf]
