# Supplementary material for: Human gene expression variability and its dependence on methylation and aging
Source: BMC Genomics. 2019 Dec 7;20:941. doi: 10.1186/s12864-019-6308-7 (PMC6898959; doi:10.1186/s12864-019-6308-7)
Supplement: Supplementary file 7 — Additional file 7. Preprocessing of Brain Samples [file 12864_2019_6308_MOESM7_ESM.pdf]

Additional File 7. Preprocessing of Brain Samples

1) K−Means clustering of brain sample age to determine
   the optimal number of age clusters using the within−cluster
   sum of squares elbow method

2) Hierarchical clustering via multiscale bootstrap of
   12 brain sample subset permutations using pvclust package
   12 Groups = Age Category(3) x Sex(2) x Tissue Type(2)

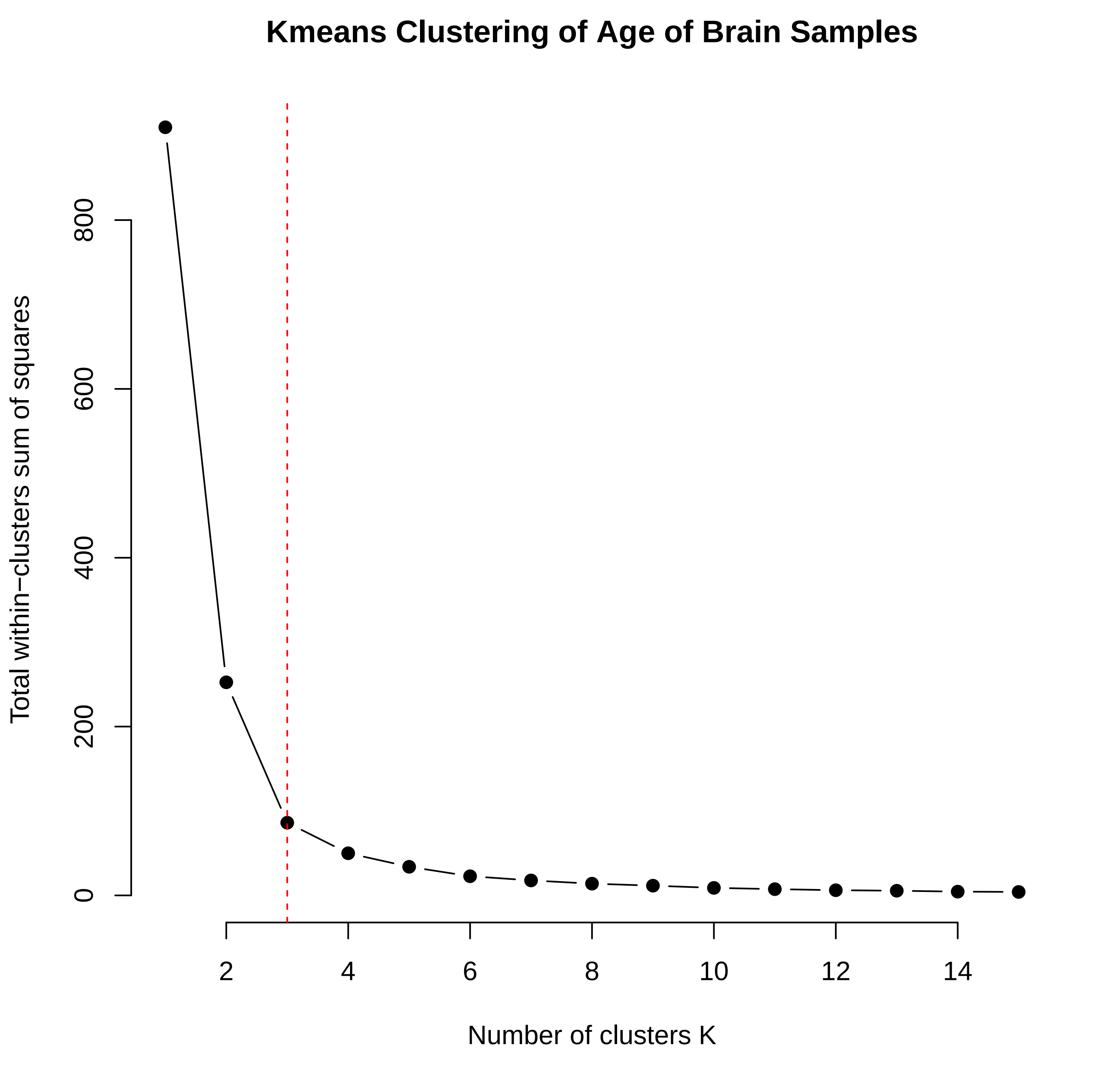

Kmeans Clustering of Age of Brain Samples
Total within−clusters sum of squares
0
200
400
600
800
2
4
6
8
10
12
14
Number of clusters K

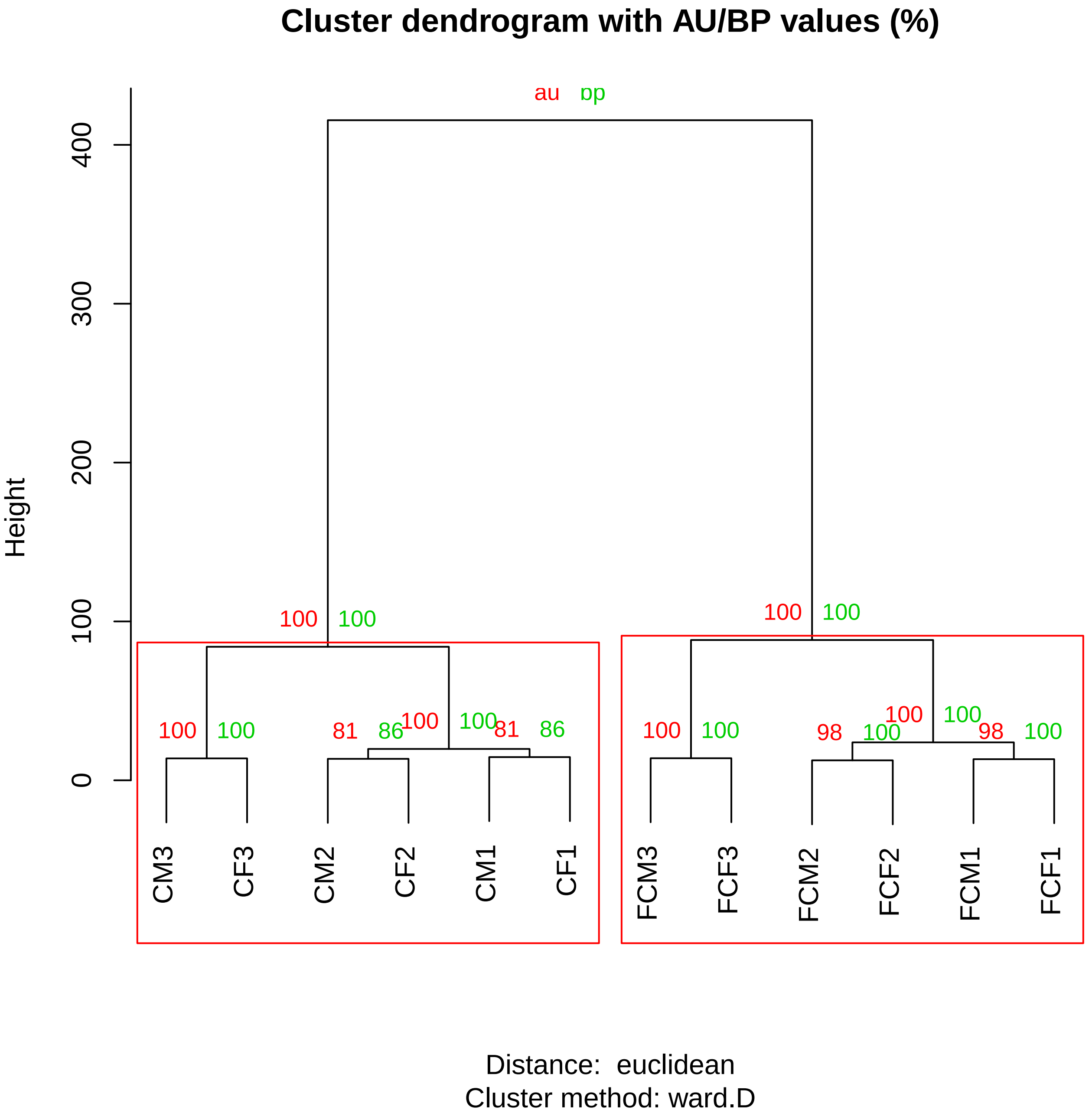

Cluster dendrogram with AU/BP values (%)
au
bp
Height
400
300
200
100
0
100
100
100
100
100
100
81
86
100
100
81
86
100
100
98
100
100
100
98
100
CM3
CF3
CM2
CF2
CM1
CF1
FCM3
FCF3
FCM2
FCF2
FCM1
FCF1
Distance: euclidean
Cluster method: ward.D
