# Supplementary material for: Human gene expression variability and its dependence on methylation and aging
Source: BMC Genomics. 2019 Dec 7;20:941. doi: 10.1186/s12864-019-6308-7 (PMC6898959; doi:10.1186/s12864-019-6308-7)
Supplement: Supplementary file 8 — Additional file 8. Methylation Variability [file 12864_2019_6308_MOESM8_ESM.pdf]

Additional File 8. Methylation Variability

Correlation between EV and MV in Cerebellum Tissue

EV Class	MV Class	Spearman Correlation
Hypervariable	Hyper−MV	−0.004880218
Hypervariable	Hypo−MV	0.064376033
Hypervariable	Non−MV	0.011039197
Hypovariable	Hyper−MV	−0.072231706
Hypovariable	Hypo−MV	−0.011245305
Hypovariable	Non−MV	0.011985907
Non−Variable	Hyper−MV	−0.065201816
Non−Variable	Hypo−MV	−0.030921975
Non−Variable	Non−MV	−0.007002520

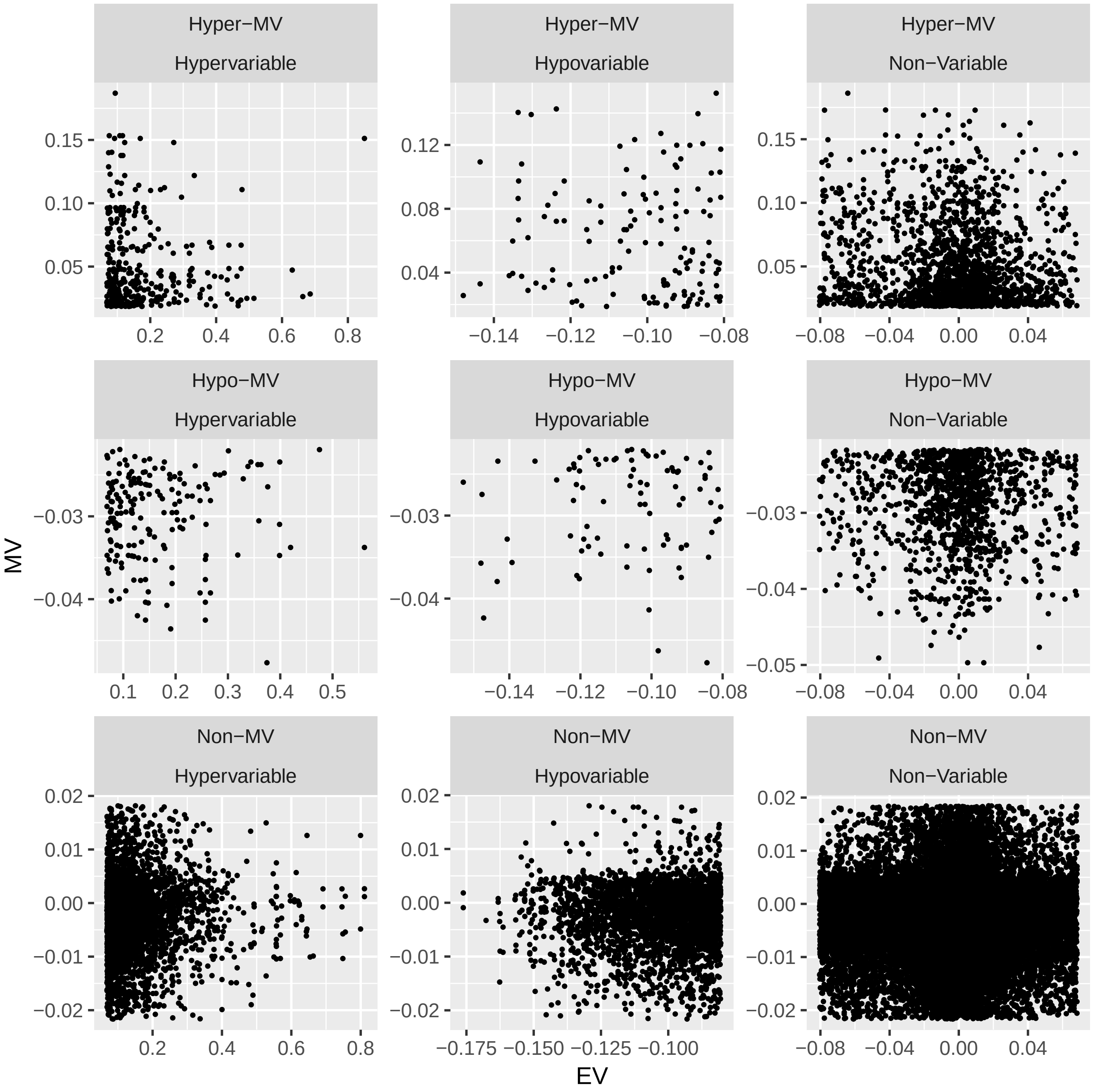
Hyper−MV
Hypervariable
Hyper−MV
Hypovariable
Hyper−MV
Non−Variable
Hypo−MV
Hypervariable
Hypo−MV
Hypovariable
Hypo−MV
Non−Variable
Non−MV
Hypervariable
Non−MV
Hypovariable
Non−MV
Non−Variable
MV
EV

Correlation between EV and MV in Frontal Cortex Tissue

EV Class	MV Class	Spearman Correlation
Hypervariable	Hyper−MV	0.011785475
Hypervariable	Hypo−MV	−0.041177866
Hypervariable	Non−MV	−0.001264391
Hypovariable	Hyper−MV	−0.043752573
Hypovariable	Hypo−MV	−0.034918772
Hypovariable	Non−MV	0.006743781
Non−Variable	Hyper−MV	−0.059431022
Non−Variable	Hypo−MV	0.046902276
Non−Variable	Non−MV	−0.007243346

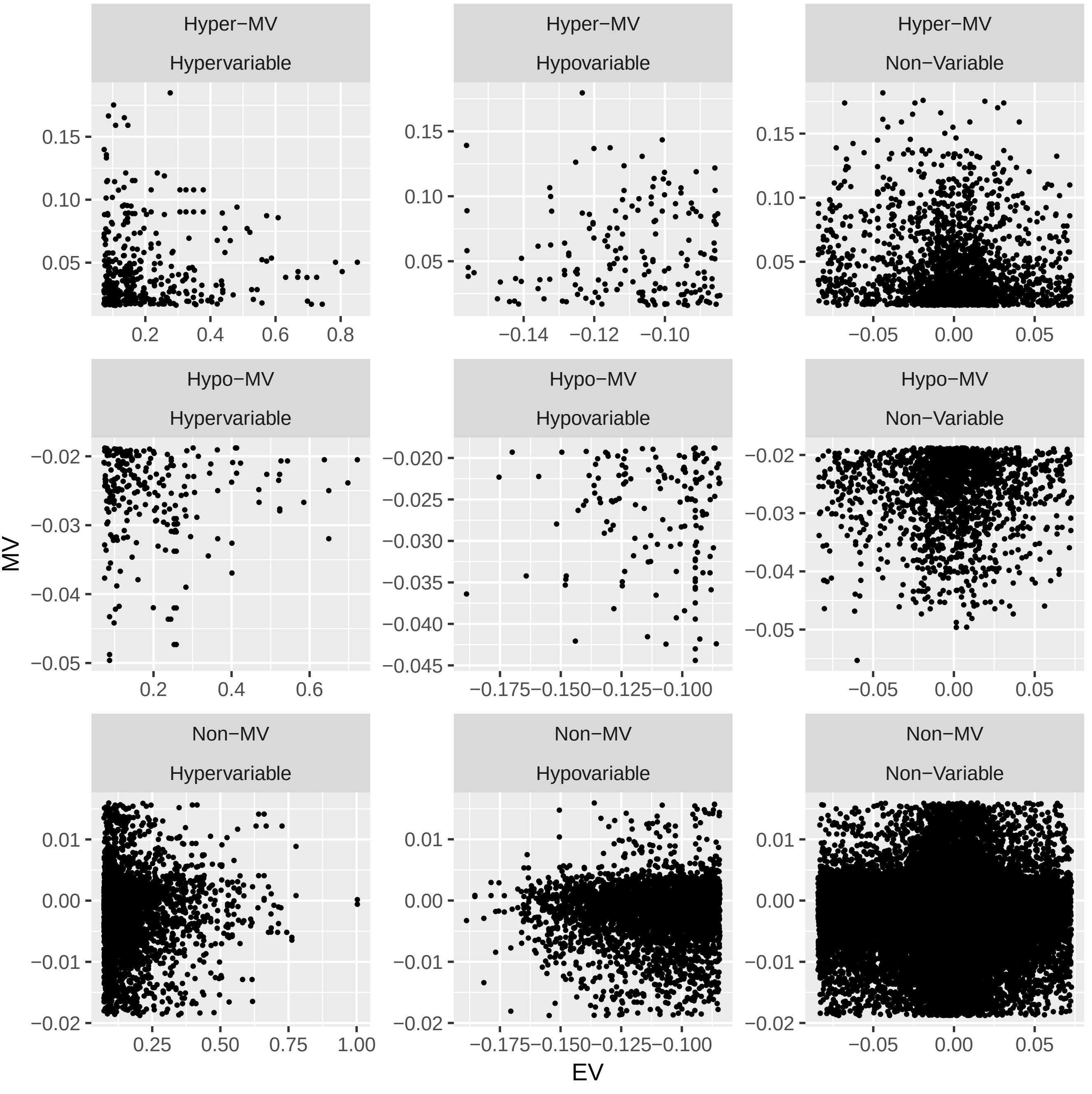

Hyper−MV
Hypervariable
Hyper−MV
Hypovariable
Hyper−MV
Non−Variable
Hypo−MV
Hypervariable
Hypo−MV
Hypovariable
Hypo−MV
Non−Variable
Non−MV
Hypervariable
Non−MV
Hypovariable
Non−MV
Non−Variable
MV
EV
